# Activation of pancreatic stellate cells attenuates intracellular Ca^2+^ signals due to downregulation of TRPA1 and protects against cell death induced by alcohol metabolites

**DOI:** 10.1038/s41419-022-05186-w

**Published:** 2022-08-29

**Authors:** Agnieszka A. Kusiak, Monika A. Jakubowska, Kinga B. Stopa, Xiaoying Zhang, Wei Huang, Julia V. Gerasimenko, Oleg V. Gerasimenko, Robert Sutton, Ole H. Petersen, Pawel E. Ferdek

**Affiliations:** 1 Department of Cell Biology, Faculty of Biochemistry, Biophysics and Biotechnology, Jagiellonian University, Krakow, Poland; 2 Malopolska Centre of Biotechnology, Jagiellonian University, Krakow, Poland; 3 West China Centre of Excellence for Pancreatitis, Institute of Integrated Traditional Chinese Medicine and Western Medicine, West China-Liverpool Biomedical Research Centre, West China Hospital, Sichuan University, Chengdu, China; 4 Liverpool Pancreatitis Research Group, Institute of Systems, Molecular and Integrative Biology, University of Liverpool and Liverpool University Hospitals NHS Foundation Trust, Liverpool, United Kingdom; 5 School of Biosciences, Cardiff University, Cardiff, United Kingdom

**Keywords:** Cell death, Ion channel signalling, Gastrointestinal diseases, Preclinical research, Physiology

## Abstract

Alcohol abuse, an increasing problem in developed societies, is one of the leading causes of acute and chronic pancreatitis. Alcoholic pancreatitis is often associated with fibrosis mediated by activated pancreatic stellate cells (PSCs). Alcohol toxicity predominantly depends on its non-oxidative metabolites, fatty acid ethyl esters, generated from ethanol and fatty acids. Although the role of non-oxidative alcohol metabolites and dysregulated Ca^2+^ signalling in enzyme-storing pancreatic acinar cells is well established as the core mechanism of pancreatitis, signals in PSCs that trigger fibrogenesis are less clear. Here, we investigate real-time Ca^2+^ signalling, changes in mitochondrial potential and cell death induced by ethanol metabolites in quiescent vs TGF-β-activated PSCs, compare the expression of Ca^2+^ channels and pumps between the two phenotypes and the consequences these differences have on the pathogenesis of alcoholic pancreatitis. The extent of PSC activation in the pancreatitis of different aetiologies has been investigated in three animal models. Unlike biliary pancreatitis, alcohol-induced pancreatitis results in the activation of PSCs throughout the entire tissue. Ethanol and palmitoleic acid (POA) or palmitoleic acid ethyl ester (POAEE) act directly on quiescent PSCs, inducing cytosolic Ca^2+^ overload, disrupting mitochondrial functions, and inducing cell death. However, activated PSCs acquire remarkable resistance against ethanol metabolites via enhanced Ca^2+^-handling capacity, predominantly due to the downregulation of the TRPA1 channel. Inhibition or knockdown of TRPA1 reduces EtOH/POA-induced cytosolic Ca^2+^ overload and protects quiescent PSCs from cell death, similarly to the activated phenotype. Our results lead us to review current dogmas on alcoholic pancreatitis. While acinar cells and quiescent PSCs are prone to cell death caused by ethanol metabolites, activated PSCs can withstand noxious signals and, despite ongoing inflammation, deposit extracellular matrix components. Modulation of Ca^2+^ signals in PSCs by TRPA1 agonists/antagonists could become a strategy to shift the balance of tissue PSCs towards quiescent cells, thus limiting pancreatic fibrosis.

## Introduction

Alcohol abuse is one of the major problems of modern societies, contributing to the development of many debilitating diseases—including acute pancreatitis (AP) and chronic pancreatitis (CP) [1, 2]. Although the incidence of pancreatic disorders is increasing globally, thus far, there is no authorised treatment available [3]. It is generally accepted that AP is initiated by toxic cytosolic Ca^2+^ signals in the pancreatic acinar cells (PACs), causing autodigestion due to premature activation of intracellular proteases and failure of mitochondrial ATP production. This results in necrosis of the enzyme-producing PACs [4, 5]. Alcohol-related AP is initiated by the powerful Ca^2+^-releasing effect of fatty acid ethyl esters (FAEEs), generated inside PACs by the non-oxidative combination of ethanol (EtOH) and fatty acids (FAs). The release of Ca^2+^ from intracellular stores, followed by Ca^2+^ release-activated Ca^2+^ entry from the extracellular fluid, causes the toxic global and sustained increase in the cytosolic Ca^2+^ concentration ([Ca^2+^]_i_), triggering alcohol-induced AP [4, 6, 7].

Pancreatic fibrosis, mediated by pancreatic stellate cells (PSCs) [1, 8], is a particularly frequent complication in alcoholic pancreatitis, but is much less common following other aetiologies [9–11]. In health, PSCs exist mainly in their quiescent phenotype, and are known to generate Ca^2+^ signals in response to physiological levels of bradykinin [4, 12, 13]. They become activated by mechanical stress or cytokines, e.g. transforming growth factor-beta (TGF-β) [12, 14, 15]. While activated PSCs play a role in tissue healing, prolonged inflammation can promote the persistent activation of these cells [12, 16, 17]. In such a scenario, PSCs repopulate tissue and deposit excessive amounts of extracellular matrix (ECM) components, resulting in organ dysfunction, diabetes, malnutrition and may contribute to an increased risk of pancreatic cancer [18, 19].

The role of Ca^2+^ in PACs and its relationship to the pathogenesis of AP is well established [4], but the signals that trigger pancreatic fibrosis are less clear. Activated cells are a dominant fraction of PSCs present during inflammation [14, 19], and it has been suggested that alcohol may contribute to PSC activation [20]. However, so far, it is unknown whether EtOH also affects Ca^2+^ homoeostasis in PSCs. Importantly, the progression from AP to fibrosis-associated CP is likely determined by the physiological consequences of PSC activation.

Here, we provide evidence that inducers of alcoholic pancreatitis act directly on PSCs, eliciting large and sustained Ca^2+^ responses in quiescent PSCs that cause cell death. However, as a result of significant alterations in Ca^2+^ homoeostasis, activated PSCs develop remarkable resistance to alcohol metabolites, which prevents them from dying. We also explored the mechanisms and candidates responsible for this phenomenon and discovered that the loss of the transient receptor potential ankyrin 1 channel (TRPA1) in activated PSCs is the main contributing factor. Our results prompt us to review the current dogmas on alcoholic pancreatitis to include the TRPA1 channel and TRPA1-dependent Ca^2+^ signals in PSCs as important players in the pathogenesis of this disease.

## Results

### EtOH and fatty acids induce pathophysiological Ca^2+^ responses in hPSCs

**Fig. 1:**
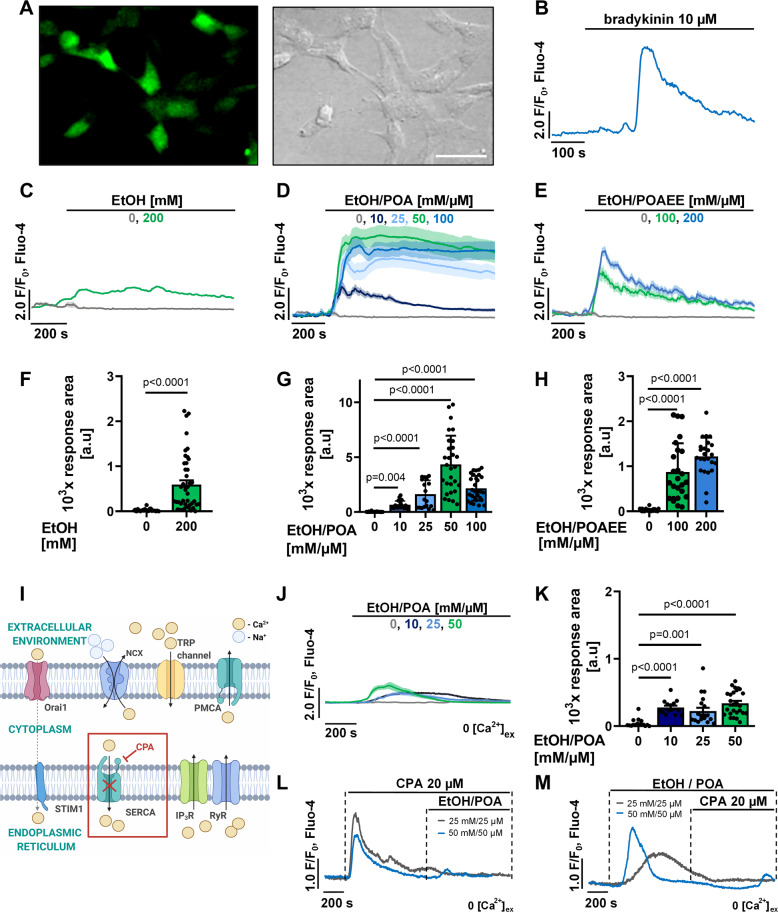
EtOH/POA and EtOH/POAEE induce Ca^2+^ responses and deplete intracellular stores in hPSCs. **A** hPSCs loaded with Fluo-4-AM (Ca^2+^ probe): left—green fluorescence of Fluo-4; right—transmitted light. Scale bar: 40 µm. **B** Sample trace showing cytosolic Ca^2+^ responses in an hPSC to bradykinin (test for the physiological phenotype). **C** Cytosolic Ca^2+^ responses (average traces ± SEM) in hPSCs to 200 mM EtOH (*n* = 42, *N* = 5); control (average traces ± SEM) shows that the application of extracellular solution alone (NaHEPES with 1 mM Ca^2+^, *n* = 30, *N* = 3) does not trigger any Ca^2+^ responses in hPSCs. **D** Cytosolic Ca^2+^ responses (average traces ± SEM) in hPSCs to different concentrations of EtOH/POA [mM/µM]: 0 (Ctrl, same as in C, *n* = 30, *N* = 3), 10 (*n* = 21, *N* = 3), 25 (*n* = 24, *N* = 3), 50 (*n* = 29, *N* = 3) and 100 (*n* = 29, *N* = 3). **E** Cytosolic Ca^2+^ responses (average traces ± SEM) in hPSCs to EtOH/POAEE [mM/µM]: 0 (Ctrl, same as in **C** and **D**, *n* = 30, *N* = 3), 100 (*n* = 27, *N* = 3), 200 (*n* = 25, *N* = 3). **F–H** Bar charts show average response areas (±SEM) as well as individual response areas (black dots), which demonstrate an increase of Ca^2+^ above the baseline levels calculated between 200 and 800 s for all traces averaged in **C**, **D** and **F**, respectively. All data were compared to the same control (perfusion with the extracellular solution alone). **I** Schematic illustration of selected aspects of the cellular Ca^2+^ signalling machinery: cyclopiazonic acid (CPA) inhibits SERCA (sarco/endoplasmic reticulum Ca^2+^-ATPase), which blocks ER refilling and leads to emptying of this Ca^2+^ store. **J** Cytosolic Ca^2+^ responses (average traces ± SEM) induced in hPSCs in the absence of extracellular Ca^2+^ by different concentrations of EtOH/POA [mM/µM]: 0 (Ctrl, *n* = 21, *N* = 3), 10 (*n* = 14, *N* = 3), 25 (*n* = 19, *N* = 3) and 50 (*n* = 26, *N* = 3). **K** Bar chart shows the increase of Ca^2+^ above the baseline levels presented as average response areas (±SEM) and individual response areas (black dots) calculated between 200 and 800 s for all traces averaged in J. **L** Cytosolic Ca^2+^ response (representative trace) to 20 µM cyclopiazonic acid (CPA), an inhibitor of SERCA (as depicted in I), in the absence of extracellular Ca^2+^. After depletion of the ER stores by CPA, a subsequent application of EtOH/POA 25 mM/25 µM (presented in grey; *n* = 34, *N* = 3) or EtOH/POA 50 mM/50 (presented in blue; *n* = 41, *N* = 3) fails to trigger further Ca^2+^ responses in hPSCs. **M** Cytosolic Ca^2+^ response (representative trace) to EtOH/POA 25 mM/25 µM (presented in grey; *n* = 14, *N* = 2) or EtOH/POA 50 mM/50 (presented in blue; *n* = 10, *N* = 3) in the absence of extracellular Ca^2+^; subsequent treatment with CPA does not trigger further Ca^2+^ release from the ER. Statistical significance was calculated with the Mann–Whitney test (for data presented in **F**) and the Kruskal–Wallis test, followed by a post hoc analysis with the Dunn test (for data presented in **G**, **H**, **K**).

Since pathophysiological Ca^2+^ signalling in PACs directly triggers pancreatic pathology, we sought to investigate whether non-oxidative metabolites of alcohol and FAs can also affect Ca^2+^ homoeostasis in hPSCs. To address this, we monitored the real-time changes in the concentration of cytosolic Ca^2+^ ([Ca^2+^]_i_) of Fluo-4-loaded hPSCs (Fig. 1A), treated with EtOH and palmitoleic acid (POA) or palmitoleic acid ethyl ester (POAEE). Our cells responded to bradykinin, as was previously reported for mouse and human PSCs (Fig. 1B) [21–23]. Our new data revealed that EtOH (200 mM) induced only a very modest increase in [Ca^2+^]_i_ of hPSCs (Fig. 1C, F). In contrast to EtOH alone, simultaneous application of EtOH with POA (concentrations of EtOH/POA were: 10 mM/10 µM, 25 mM/25 µM, 50 mM/50 µM and 100 mM/100 µM) caused a large and sustained elevation of [Ca^2+^]_i_ in hPSCs (Fig. 1D). This increase was dose-dependent and most prominent for EtOH/POA 50 mM/50 µM (Fig. 1G). EtOH/POA 100 mM/100 µM induced such a dramatic [Ca^2+^]_i_ overload that it killed a substantial fraction of hPSCs during the experiment, leading to the seemingly decreased response areas (Fig. 1G). Similarly, treatment with EtOH/POAEE 100 mM/100 µM and 200 mM/200 µM resulted in a global and prolonged elevation of [Ca^2+^]_i_ in hPSCs (Fig. 1E) and the higher concentration had a more prominent effect (Fig. 1H).

In order to characterise the above Ca^2+^ signals, we carried out imaging experiments in which we disrupted intracellular Ca^2+^ homoeostasis either by removing extracellular Ca^2+^ or by incubation of cells with cyclopiazonic acid (CPA), a reversible inhibitor of the serco-endoplasmic reticulum Ca^2+^ ATPase (SERCA) (Fig. 1I) [24]. While EtOH/POA (10 mM/10 µM, 25 mM/25 µM and 50 mM/50 µM) induced elevation of [Ca^2+^]_i_ in hPSCs in the absence of extracellular Ca^2+^, these responses were mostly modest and, unlike in the presence of extracellular Ca^2+^, returned to the baseline levels during the time course of the experiment (Fig. 1J). The magnitude of Ca^2+^ responses was very similar for all tested concentrations and did not show a clear concentration dependence (Fig. 1K). Inhibition of SERCA by CPA unmasked passive Ca^2+^ leak from the endoplasmic reticulum (ER) and led to emptying of the ER Ca^2+^ stores. After the application of 20 µM CPA, both EtOH/POA 25 mM/25 µM and 50 mM/50 µM failed to induce further Ca^2+^ signals (Fig. 1L). When the treatment was applied in reverse order, EtOH/POA 25 mM/25 µM or 50 mM/50 µM triggered a temporal [Ca^2+^]_i_ increase, whereas a subsequent addition of 20 µM CPA did not have any effect (Fig. 1M). This suggests that EtOH/POA releases Ca^2+^ primarily from the ER store.

### EtOH and POA(EE) cause activation of hPSC in vivo but not in vitro

**Fig. 2:**
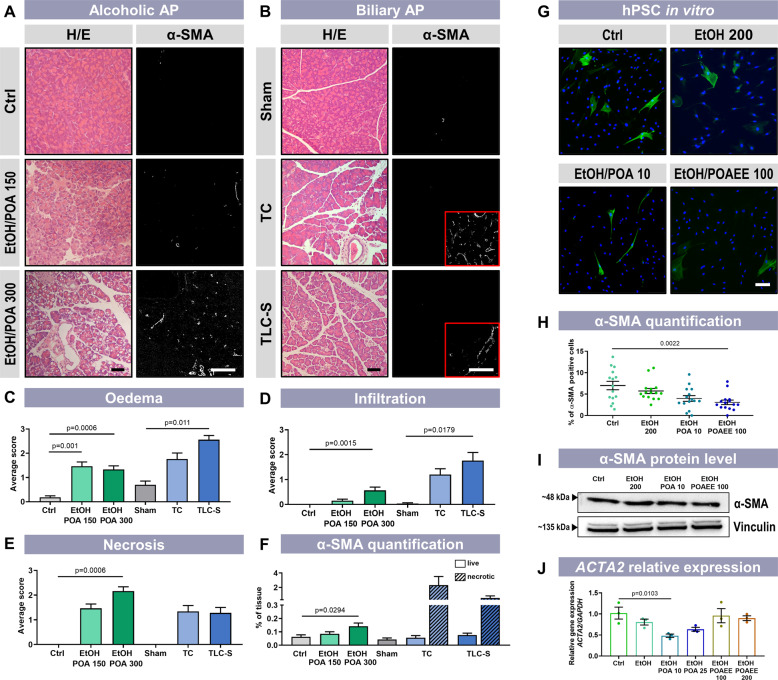
Ethanol metabolites increase α-SMA expression in the alcoholic AP mouse model in vivo but not in vitro in hPSCs. **A** Alcoholic AP was induced by intraperitoneal injection of ethanol (1.5 g/kg) and palmitoleic acid (POA, 150 or 300 mg/kg, *n* = 6 for both) in the presence of PEG 200 (1 g/kg), twice at 1 h intervals. Control mice received saline injections (*n* = 6). H/E staining shows an increasing severity of inflammation with increasing doses of POA (left panels). IHF staining shows a dose-dependent increase in α-SMA expression (presented in white) throughout pancreatic tissue (right panels). Scale bars: 50 µm. **B** Two models of bile acid-induced AP: taurocholate (TC)-elicited acute pancreatitis (TC-AP) was induced by retrograde pancreatic ductal injection with 1% TC (5 µl/min over 10 min by infusion pump, *n* = 5); taurolithocholic acid 3-sulfate (TLC-S)-elicited acute pancreatitis (TLC-S-AP) was induced by retrograde pancreatic ductal injection with 3 mM TLC-S (5 µl/min over 10 min by infusion pump, *n* = 5); for both models, saline injections (sham) were experimental controls (*n* = 3). Although H/E staining confirms severe inflammation, α-SMA expression is limited only to necrotic areas (inserts shown in red frames) but is absent in live tissue. Scale bars: 50 µm. **C–E** Histological scoring of H/E staining was done on ten random fields of view by “blinded” investigators. The severity of each parameter – oedema (**C**), inflammatory cell infiltration (**D**) and acinar cell necrosis (**E**)—was scored using a 4-level grade [28, 51]. Results were presented as mean ± SEM. **F** α-SMA expression was quantified as a ratio of α-SMA-positive area to total tissue area (QuPath Software) [52]. Calculations were carried out in ten random fields of view by “blinded” investigators. Where applicable, live and necrotic areas were identified in transmitted light images. Results were presented as mean ± SEM. Statistical significance was calculated using Welch’s ANOVA with Games-Howell’s post hoc test (alcoholic AP) and Kruskal–Wallis and Dunn’s post hoc test (biliary AP and infiltration in alcoholic AP). **G** Activation of human PSCs was assessed after incubation with EtOH 200 mM, EtOH/POA 10 mM/10 µM or EtOH/POAEE 100 mM/100 µM for 24 h. The expression of α-SMA (green) is an indicator of the activated phenotype. Cell nuclei are shown in blue. Scale bar: 100 µm. **H** Calculated proportion of α-SMA-positive cells (±SEM) in immunostaining presented in G. Cells were counted in five random fields of view for each of three biological replicates. Individual values are presented as dots. **I** Representative Western blot for α-SMA in hPSC after incubation with EtOH 200 mM, EtOH/POA 10 mM/10 µM or EtOH/POAEE 100 mM/100 µM for 24 h. Vinculin was used as a loading control. **J** Relative gene expression of *ACTA2* (±SEM) in hPSC after incubation with given concentrations of ethanol, POA or POAEE (EtOH 200 mM; EtOH/POA 10 mM/10 µM; EtOH/POAEE 100 mM/100 µM) for 24 h. Expression was normalised to glyceraldehyde-3-phosphate dehydrogenase (*GAPDH;* *N* = 3). Statistical significance was calculated using the one-way ANOVA test followed by a post hoc analysis with the Dunnett test.

Unlike bile-induced pancreatitis, chronic alcoholic pancreatitis is often associated with fibrosis [25, 26]. Given the above, we decided to test whether EtOH/POA is capable of activating PSCs in vivo. For this purpose, we used three different mouse models: alcoholic AP induced by EtOH (1.35 g/kg) and POA (150 mg/kg and 300 mg/kg) [27]; and two models of biliary AP, induced by taurolithocholic acid (TC-AP) or taurolithocholic acid 3-sulfate (TLC-S-AP) [28], all recently reviewed [29]. H/E staining (Fig. 2A, B, left panels) and histological scoring of the H/E-stained pancreatic tissue (Fig. 2C–E) confirm that all models demonstrated typical clinical characteristics of AP, such as prominent oedema, infiltration of immune cells in the parenchyma, and patchy or diffused acinar cell necrosis. POA at 300 mg/kg was substantially more harmful compared to POA at 150 mg/kg (Fig. 2A, left panel). Immunofluorescence staining for alpha-smooth muscle actin (α-SMA) was used to assess the extent of PSC activation in vivo and, therefore, the potential of the tissue to develop fibrotic complications. A scattered pattern of α-SMA staining was present in the whole tissue in the alcoholic model (Fig. 2A, right panels). In the biliary models, the staining was restricted to necrotic regions of the tissue damaged by the infusion of bile salts and was completely absent in the healthy regions (Fig. 2B, right panels). Quantification of the fluorescence signal showed a clear, statistically significant increase of α-SMA expression in the group treated with EtOH and POA 300 mg/kg (Fig. 2F). However, in vitro experiments revealed that EtOH and FAs do not cause upregulation of α-SMA in PSCs directly, as shown by immunofluorescence staining of hPSCs fixed after 24 h treatment with EtOH/POA(EE) (Fig. 2G, H), Western blot for α-SMA (Fig. 2I and Supplementary Fig. 1) and comparison of *ACTA2* transcript levels (Fig. 2J). The above results indicate that the activation of PSCs in vivo, as evidenced by the expression pattern of α-SMA, was not due to the direct effect of EtOH/POA(EE) on these cells.

### Activation of hPSCs has a profound effect on pathophysiological Ca^2+^ responses

**Fig. 3:**
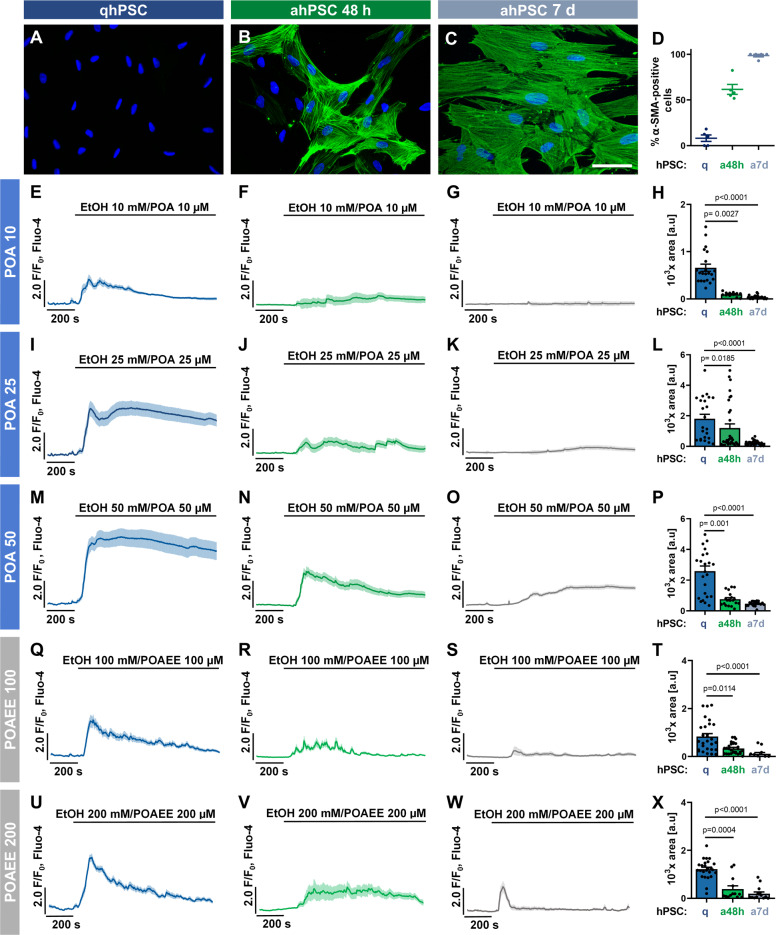
Ca^2+^ responses induced by ethanol metabolites are reduced in activated human PSCs. **A**–**C** Activation of hPSCs was induced by incubation with TGF-β (5 ng/ml) for 48 h or 7 days. The expression of α-SMA (green) is an indicator of the activated myofibroblast-like phenotype. Cell nuclei are shown in blue. Scale bar: 30 µm. **D** Calculated proportion of α-SMA-positive cells (±SEM) in immunostaining presented in **A**–**C**. Cells were counted in five random fields of view for each staining. Individual values are presented as dots. **E–G** The average traces (±SEM) show cytosolic Ca^2+^ responses to 10 mM EtOH and 10 µM POA in quiescent hPSCs (*n* = 21; **E**), TGF-β-activated hPSCs for 48 h (*n* = 12; **F**) and TGF-β-activated hPSCs for 7 days (*n* = 30; **G**). **H** The bar graph shows the average response amplitude (±SEM) calculated between 200 and 800 s for the traces averaged in **E**–**G**. Individual values are presented as dots. **I**–**K** The average traces (±SEM) show cytosolic Ca^2+^ responses to 25 mM EtOH and 25 µM POA in quiescent hPSCs (*n* = 24; **I**), TGF-β-activated hPSCs for 48 h (*n* = 23; **J**), and TGF-β-activated hPSCs for 7 days (*n* = 27; **K**). **L** The bar graph shows the average response amplitude (±SEM) calculated between 200 and 800 s for the traces averaged in I–K. Individual values are presented as dots. **M**–**O** The average traces (±SEM) show cytosolic Ca^2+^ responses to 50 mM EtOH and 50 µM POA in quiescent hPSCs (*n* = 29; **M** presented previously in Fig. 1D), TGF-β-activated hPSCs for 48 h (*n* = 23; **N**), and TGF-β-activated hPSCs for 7 days (*n* = 20; **O**). **P** The bar graph shows the average response amplitude (±SEM) calculated between 200 and 800 s for the traces averaged in **M**–**O**. Individual values are presented as dots. **Q**–**S** The average traces (±SEM) show cytosolic Ca^2+^ responses to 100 mM EtOH and 100 µM POAEE in quiescent hPSCs (*n* = 27; **Q**), TGF-β-activated hPSCs for 48 h (n = 28; **R**) and TGF-β-activated hPSCs for 7 days (n = 13; **S**). **T** The bar graph shows the average response amplitude (±SEM) calculated between 200 and 800 s for the traces averaged in **Q**–**S**. Individual values are presented as dots. **U**–**W** The average traces (±SEM) show cytosolic Ca^2+^ responses to 200 mM EtOH and 200 µM POAEE in quiescent hPSCs (*n* = 25; **U**), TGF-β-activated hPSCs for 48 h (*n* = 13; **V**) and TGF-β-activated hPSCs for 7 days (*n* = 15; **W**). **X** The bar graph shows the average response amplitude (±SEM) calculated between 200 and 800 s for the traces averaged in **U**–**W**. Individual values are presented as dots. Statistical significance was calculated with the Kruskal–Wallis test, followed by a post hoc analysis with Dunn's test.

Since EtOH/POA(EE) induce substantial [Ca^2+^]_i_ responses in hPSCs in vitro, and alcoholic AP is associated with the activation of PSCs in vivo, we next examined whether the phenotype transition could affect Ca^2+^ homoeostasis and contribute to the development of the disease and its complications. To achieve this, we applied an in vitro model of hPSCs activated by TGF-β (5 ng/ml) either for 48 h (ahPSC 48 h) or 7 days (ahPSC 7 days). The phenotype of hPSCs was confirmed by immunofluorescence staining for α-SMA (Fig. 3A–C). After 48 h incubation, ~60% of cells were positive for α-SMA; and after 7 days, essentially all hPSCs were activated (Fig. 3D). Ca^2+^ elevation induced by EtOH/POA 10 mM/10 µM was almost completely abolished in ahPSCs 48 h and 7 d compared to qhPSCs (Fig. 3E–H). EtOH/POA 25 mM/25 µM induced substantially reduced Ca^2+^ responses in ahPSCs (Fig. 3I–L); and a similar reduction was observed in ahPSCs treated with EtOH/POA 50 mM/50 µM (Fig. 3M–P). Very notable attenuation of Ca^2+^ responses also occurred in ahPSCs 48 h and 7 d treated with EtOH/POAEE 100 mM/100 µM (Fig. 3Q–T) and 200 mM/200 µM (Fig. 3U–X). For most of the above treatments, Ca^2+^ signals in ahPSCs were not only reduced, but also more transient, with a tendency to return to the basal levels in the time course of the experiment.

### Expression of Ca^2+^ channels and transporters changes in ahPSCs

**Fig. 4:**
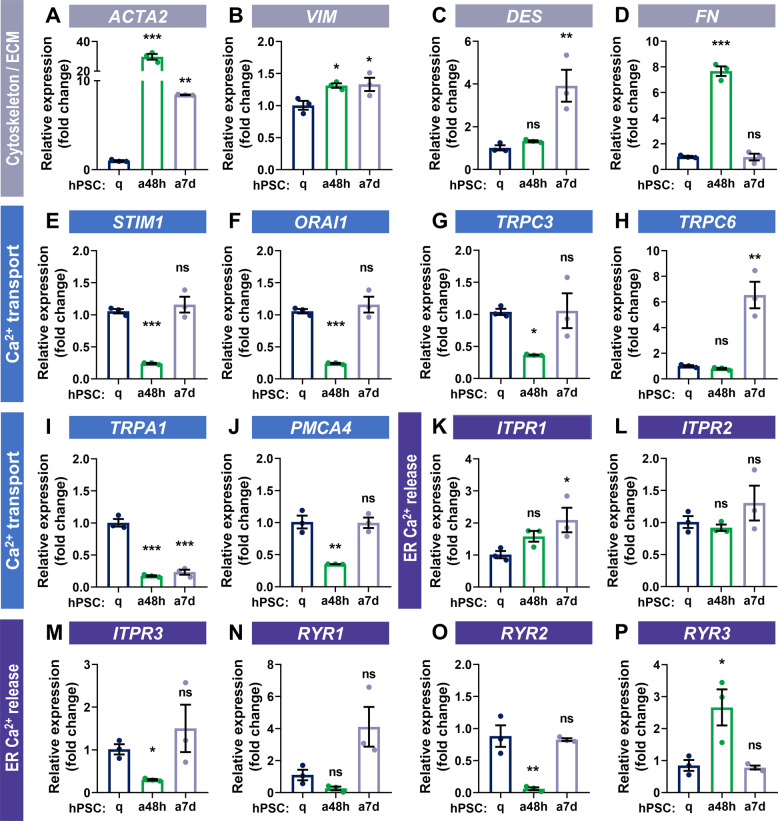
Expression of selected genes in quiescent and TGF-β-activated human PSCs. Relative mRNA levels (qPCRs) of selected targets (±SEM) in quiescent hPSC (blue) hPSCs activated with TGF-β (5 ng/ml) for 48 h (green) and for 7 days (grey): **A** *actin alpha 2, smooth muscle* (*ACTA2*); **B** *vimentin* (*VIM*); **C** *desmin* (*DES*); **D** *fibronectin* (*FN*); **E** *stromal interaction molecule 1* (*STIM1*); **F** *calcium release-activated calcium channel protein 1* (*ORAI1*); **G** *transient receptor potential cation channel, subfamily C, member 3* (*TRPC3*); **H** *transient receptor potential cation channel, subfamily C, member 6* (*TRPC6*); **I** *transient receptor potential ankyrin 1* *channel* (*TRPA1*); **J** *plasma membrane calcium ATPase, isoform 4* (*PMCA4*); **K** *inositol 1,4,5-trisphosphate receptor type 1* (*ITPR1*); **L** *inositol 1,4,5-trisphosphate receptor type 2* (*ITPR2*); **M** *inositol 1,4,5-trisphosphate receptor type 3* (*ITPR3*); **N** *ryanodine receptor type 1* (*RYR1*); **O** *ryanodine receptor type 2* (*RYR2*); **P** *ryanodine receptor type 3* (*RYR3*). Expression was normalised to glyceraldehyde-3-phosphate dehydrogenase (*GAPDH)* (*n* = 2, *N* = 3). Statistical significance was calculated using the one-way ANOVA test followed by a post hoc analysis with the Dunnett test. ns non-significant difference, *p < 0.05, **p < 0.01; ***p < 0.001.

Since ahPSCs show a different pattern of Ca^2+^ responses compared to qhPSCs, it is likely that not only the expression of cytoskeletal proteins changes upon PSC activation, but also proteins involved in Ca^2+^ homoeostasis become up/down-regulated in ahPSCs. To test this hypothesis, we compared the expression of selected mRNA targets encoding cytoskeletal or ECM proteins as well as proteins engaged in Ca^2+^ transport and release between qhPSCs and ahPSCs (48 h and 7 days). Activation of hPSCs was evidenced by upregulated expression of the *ACTA2* gene, encoding α-SMA (Fig. 4A). After the initial burst of *ACTA2* expression in 48 h post-activation with TGF-β, the expression of *ACTA2* stabilised at a somewhat lower level after 7 days (Fig. 4A). *VIM* (encoding Vimentin) increased marginally both in ahPSCs 48 h and 7 days (Fig. 4B). *DES* (Desmin) was elevated only after 7 days of incubation with TGF-β (Fig. 4C); in contrast, *FN* (Fibronectin) showed an increase in ahPSCs 48 h, but not in ahPSCs 7 days (Fig. 4D). The expression of several tested Ca^2+^ handling proteins initially decreased at 48 h, only to return to baseline levels after a 7-day incubation: *STIM1* (Fig. 4E), *ORAI1* (Fig. 4F), *TRPC3* (Fig. 4G), *PMCA4* (Fig. 4J), *ITPR3* (Fig. 4M) and *RYR2* (Fig. 4O). This was inversely correlated with a particularly high expression of *ACTA2* in ahPSCs 48 h (Fig. 4A). *TRPC6* was only elevated after 7 days after activation (Fig. 4H); *ITPR1* (Fig. 4K), *ITPR2* (Fig. 4L) and *RYR1* (Fig. 4N) did not change much in ahPSCs; and *RYR3* (Fig. 4P) was only slightly increased after 48 h. However, only one target of the tested mRNAs was consistently decreased in both ahPSCs 48 h and 7 d: *TRPA1* (Fig. 4I).

### EtOH/POA-induced Ca^2+^ responses and cell death in hPSCs are regulated by TRPA1

**Fig. 5:**
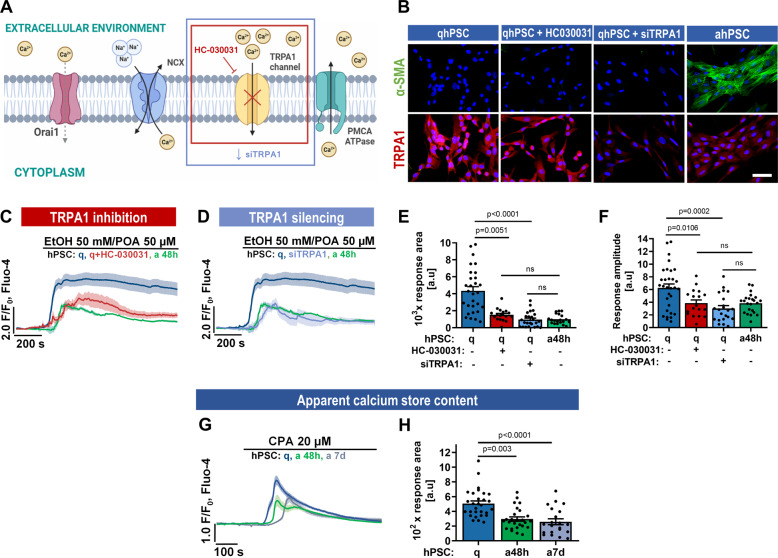
Ca^2+^ responses to EtOH/POA are predominantly dependent on TRPA1. **A** Schematic illustration of the inhibition experiment: TRPA1 is a Ca^2+^ channel inhibited by HC-030031. **B** The expression of the activation marker α-SMA (upper panel, green) and TRPA1 (lower panel, red) was evaluated in quiescent hPSCs, quiescent hPSCs treated with TRPA1 inhibitor for 48 h, and quiescent hPSCs with TRPA1 knockdown and TGF-β-activated hPSCs for 48 h. Cell nuclei are shown in blue. Scale bar: 50 µm. **C** The average traces (±SEM) show cytosolic Ca^2+^ responses to EtOH/POA 50 mM/50 µM in quiescent hPSCs (*n* = 29, *N* = 3; navy; presented previously in Fig. 1D), TGF-β-activated hPSCs for 48 h (*n* = 23, *N* = 3; green; presented previously in Fig. 3N) and quiescent hPSCs incubated with HC-030031 for 5 min (*n* = 15, *N* = 3; red). **D** Average traces (±SEM) show cytosolic Ca^2+^ responses to EtOH/POA 50 mM/50 µM in quiescent hPSCs (*n* = 29, *N* = 3; navy; presented previously in Figs. 1D, 5C), TGF-β-activated hPSCs for 48 h (*n* = 23, *N* = 3; green; presented previously in Figs. 3N, 5C) and quiescent hPSCs with silenced expression of *TRPA1* (*n* = 23, *N* = 3; blue). **E** The bar graph shows the average response area (±SEM) calculated between 200 and 1200 s for the traces averaged in **C** and **D**. **F** The bar graph shows the average response amplitude (±SEM) between 200 and 1200 s for the traces averaged in **C** and **D**. **G** The average traces ± SEM show cytosolic Ca^2+^ responses to the application of cyclopiazonic acid (CPA), an inhibitor of the SERCA pump, in quiescent hPSCs (*n* = 29, *N* = 3; q; navy), TGF-β-activated PSCs for 48 h (*n* = 28, *N* = 3; a48 h; green) TGF-β-activated PSCs for 7 d (*n* = 14, *N* = 3; a7d; grey). **H** Bar chart shows the average response area (±SEM) calculated between 200 and 800 s for the traces averaged in G. Statistical significance was calculated with the Kruskal–Wallis test, followed by a post hoc analysis with Dunn's test. The ROUT test was used to identify outliers in **F**.

TRPA1 caught our interest since its expression in qhPSCs could contribute to [Ca^2+^]_i_ overload in these cells but not in ahPSCs. To further investigate this, we inhibited TRPA1 with HC-030031 or silenced *TRPA1* expression with selective siRNA in qhPSCs (Fig. 5A). siRNA decreased levels of the TRPA1 channel, as shown by immunofluorescence staining for α-SMA and TRPA1 (Fig. 5B). This downregulation was very similar to what occurred upon activation of hPSCs. Importantly, neither pharmacological inhibition nor TRPA1 suppression caused upregulation of α-SMA on its own (Fig. 5B). However, both pharmacological inhibition and silencing of TRPA1 resulted in a very significant decrease in EtOH/POA-induced [Ca^2+^]_i_ elevation, almost perfectly mirroring the responses recorded in ahPSCs 48 h (Fig. 5C, D, respectively). The average area and amplitude of these responses were not significantly different from the respective values for ahPSC 48 h (Fig. 5E, F), suggesting that TRPA1 is a major contributor to the Ca^2+^ overload caused by EtOH/POA in qhPSCs. Finally, we wanted to test whether the reduced Ca^2+^ influx that results from decreased expression of TRPA1 could affect the stored Ca^2+^ content in ahPSCs compared to qhPSCs. In fact, CPA-induced [Ca^2+^]_i_ elevation was significantly lower in ahPSCs 48 h and 7 days compared to qhPSCs (Fig. 5G), as also shown by the analysis of the response areas (Fig. 5H), suggesting that the releasable pool of ER Ca^2+^ is likely to decrease in ahPSCs.

### Activated hPSCs are more resistant to cell death and this effect can be attained in quiescent hPSCs by inhibition of TRPA1

**Fig. 6:**
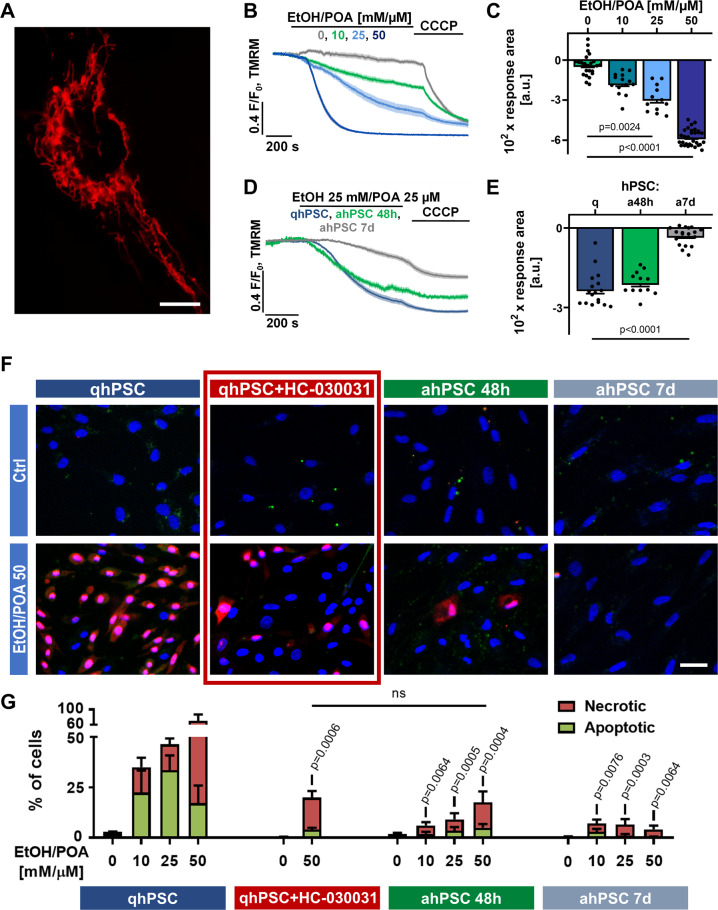
Activated hPSCs are resistant to EtOH/POA-induced loss of mitochondrial potential (Δψ) as well as cell death. **A** Image shows mitochondrial localisation of TMRM in hPSCs. Scale bar: 10 μm. **B** The average traces (±SEM) show a decrease in TMRM fluorescence recorded in hPSCs in response to different concentrations of EtOH/POA: 0 (Ctrl, *n* = 24, *N* = 3), 10 (*n* = 14, *N* = 3), 25 (*n* = 12, *N* = 3) and 50 (*n* = 30, *N* = 3) mM/µM, respectively. CCCP (1 μM) was applied at the end of each experiment to attain the maximal decrease of Δψ. **C** The bar graph shows the average decrease (±SEM) below the baseline levels calculated between 200 and 800 s for the traces averaged in B. **D** Average traces (±SEM) show a decrease in TMRM fluorescence recorded in response to 25 mM EtOH and 25 µM POA in quiescent hPSCs (blue; *n* = 16, *N* = 3), TGF-β-activated PSCs for 48 h (green; *n* = 11, *N* = 3) and TGF-β-activated PSCs for 7 days (grey; *n* = 17, *N* = 3). CCCP (1 and 10 μM) was applied at the end of each experiment to attain the maximal decrease of Δψ. **E** The bar graph shows the average decrease (±SEM) below the baseline levels calculated between 200 and 800 s for the traces averaged in D. **F** Representative images of the staining of hPSCs with annexin V-FITC and propidium iodide after 30 min incubation with 50 mM EtOH and 50 µM POA: quiescent hPSCs (first column), quiescent hPSCs incubated with TRPA1 inhibitor HC-030031 (second column), TGF-β-activated hPSCs for 48 h (third column) and TGF-β-activated PSCs for 7 days (fourth column). Scale bar: 10 μm. **G** The bar charts show the proportion of apoptotic (green) and necrotic (red) cells ±SEM (for all groups *N* = 3) calculated from the staining presented in **F**. Statistical significance was calculated for the combined dead cells (apoptotic + necrotic) for a given concentration of EtOH/POA between qhPSC vs qhPSC + HC-030031, ahPSC 48 h and ahPSC 7 days, using one-way ANOVA followed by a post hoc analysis with the Dunnett’s T3 test. The significance between qhPSC + HC-030031 and ahPSC 48 h was calculated using the *t*-test.

The activity of a number of Ca^2+^ pumps (e.g. PMCA and SERCA), transporters (e.g. NCX) or channels (e.g. STIM1-Orai1) is directly or indirectly dependent on cellular ATP levels. Prolonged elevation of [Ca^2+^]_i_ could substantially affect mitochondrial functions, deplete ATP and lead to induction of cell death [30]. To test whether the differences in Ca^2+^ responses between qhPSCs and ahPSCs (48 h and 7 days) are reflected in and by the condition of mitochondria, we applied tetramethylrhodamine methyl ester (TMRM), a fluorescent probe that accumulates in healthy mitochondria with intact membrane potential (Fig. 6A). The application of EtOH/POA disrupted the mitochondrial potential in qhPSCs (Fig. 6B, C). This effect was relatively modest in response to low concentrations of EtOH/POA (10 mM/10 µM, no statistical significance), but EtOH/POA 25 mM/25 µM and 50 mM/50 µM caused very substantial declines in mitochondrial potential (Fig. 6C). In contrast, the effect of EtOH/POA on mitochondria was much less pronounced in ahPSCs: while EtOH/POA 25 mM/25 µM caused only marginally lower decrease in the mitochondrial potential of ahPSCs 48 h, in ahPSCs 7 d the loss of mitochondrial potential was markedly inhibited compared to qhPSCs (Fig. 6D, E). Given that both Ca^2+^ signalling and the condition of mitochondria control cell fate, we compared the extent of EtOH/POA-induced cell death in qhPSCs and ahPSCs using annexin V-FITC, propidium iodide (PI) and Hoechst 33258. Incubation with EtOH/POA at different concentrations for 30 min caused a dose-dependent increase in cell death of qhPSCs: apoptosis was the most prevalent at lower concentrations (10 mM/10 µM and 25 mM/25 µM), whereas necrosis was dominant in the group treated with EtOH/POA 50 mM/50 µM (Fig. 6F, G). However, in ahPSCs 48 h, there was a substantial reduction in total cell death (apoptosis and necrosis) in favour of living cells for all tested concentrations of EtOH/POA (Fig. 6F, G). This effect was even more pronounced in ahPSCs 7 days and was no longer dose-dependent in the range of EtOH/POA concentrations used (Fig. 6F, G). Importantly, pharmacological inhibition of TRPA1 protected qhPSCs from cell death induced by EtOH/POA 50 mM/50 µM, reducing apoptosis and necrosis to the levels seen in ahPSCs 48 h (Fig. 6F, G).

## Discussion

EtOH-induced damage to the pancreas occurs via products of non-oxidative alcohol metabolism: FAEEs generated from EtOH and FAs, such as POA [4, 7]. FAEEs are present in human plasma after EtOH ingestion and were found in particularly high amounts in the pancreata of subjects intoxicated with EtOH at the time of death [31, 32]. Recently, FAEEs have been suggested as candidate biomarkers of pancreatitis [33]. FAEEs are formed by esterification of FAs with EtOH catalysed by FAEE synthases, or by transesterification of EtOH to acyl-coenzyme A by acyl-coenzyme A:ethanol O-acyltransferase [34]. Since the pancreatic tissue is characterised by high FAEE synthase activity and FAEE hydrolase activity, comparable to that of the liver [34, 35], treatment with EtOH and POA will generate POAEE intracellularly, and vice versa. FAEEs can accumulate at the inner mitochondrial membrane [36], where they become broken down to FAs and ethanol by carboxylesterase [34].

The toxicity of alcohol metabolites is generally attributed to the generation of excessive cytosolic Ca^2+^ signals in PACs. This causes premature intracellular activation of digestive enzymes and failure of mitochondrial ATP production, leading to necrosis, which in turn leads to a generation of Ca^2+^ signals in PSCs and pancreatic immune cells [4, 7]. Although this clarifies the core mechanism of pancreatic autodigestion, inflammation, and tissue necrosis, it does not explain why PSC-mediated fibrosis is a particularly common complication of alcoholic pancreatitis. Since Ca^2+^ signals play a major role in the mechanism of EtOH-induced toxicity in PACs, in this study, we have focused on the Ca^2+^ effects of alcohol metabolites on PSCs. EtOH alone applied at high concentration only elicited a modest elevation of baseline [Ca^2+^]_i_ in qhPSCs (Fig. 1C), which is similar to previous observations made in PACs [4, 37]. Importantly, both EtOH/POA and EtOH/POAEE consistently induced global and sustained elevations of [Ca^2+^]_i_ in qhPSCs, in a dose-dependent manner (Fig. 1D, E, G, H), suggesting that alcohol metabolites are detrimental, not only to PACs but also to PSCs.

While the influx of extracellular Ca^2+^ is a significant part of EtOH/POA(EE)-induced responses, it is the release of Ca^2+^ from intracellular stores that is the initiating signal. This is illustrated by the fact that although the responses to EtOH/POA in qhPSCs were substantially diminished in the absence of extracellular Ca^2+^, they were not completely abolished (Fig. 1J, K). In PACs, the ER is the primary source of toxic [Ca^2+^]_i_ elevation induced by alcohol metabolites and sustained incubation of PACs with POAEE or POA in the absence of external Ca^2+^ depletes the ER of Ca^2+^ [38]. However, Ca^2+^ entry from the extracellular space through Ca^2+^ release-activated Ca^2+^ channels is an absolute requirement for acinar necrosis to occur [4].

In qhPSCs, emptying the ER with CPA prevented EtOH/POA-driven Ca^2+^ release from intracellular stores (Fig. 1L) and CPA failed to induce any Ca^2+^ responses when it was applied after EtOH/POA, suggesting that the ER stores had already been depleted (Fig. 1M). While FAEEs in PACs cause toxic elevation of [Ca^2+^]_i_ predominantly through Ca^2+^ release from the ER via IP_3_Rs [4], FAs act on mitochondria and disrupt ATP production, which results in Ca^2+^ pump failure (SERCA, PMCA) [38]. Our results show that EtOH/POA induces diminished and transient Ca^2+^ signals in qhPSCs in the absence of extracellular Ca^2+^ (Fig. 1J), resembling the elevation of [Ca^2+^]_i_ triggered by the inhibition of SERCA by CPA (Fig. 1M). This similarity is consistent with the failure of Ca^2+^ ATPases due to the lack of ATP contributing to Ca^2+^ overload in qhPSCs exposed to EtOH/POA or POAEE in the presence of extracellular Ca^2+^. This notion is strengthened by the fact that EtOH/POA induces a loss of mitochondrial potential (Fig. 6B, C), which is inevitably associated with a substantial drop in cellular ATP levels. Indeed, FAs were previously shown to disrupt mitochondrial metabolism and inhibit respiration by uncoupling oxidative phosphorylation and opening the mitochondrial permeability transition pore [38, 39].

In this study, we have tested the capacity of different inducers of pancreatic pathology, i.e. bile acids *vs* ethanol metabolites, to activate PSCs in three mouse models of AP. Our new data demonstrate a marked tissue-wide elevation in α-SMA expression in the alcoholic model (Fig. 2A, B). However, this activation is clearly not due to a direct action of alcohol metabolites on PSCs, since EtOH/POA and EtOH/POAEE failed to activate qhPSCs in vitro (Fig. 2G–J). Previously, it has been suggested that rat PSCs could be activated by EtOH in vitro [20], but this does not seem to be the case for human PSCs. This issue requires further investigation as results from cultured cells may also differ from those in acutely isolated cells.

Experimental alcoholic AP shows a different pattern from experimental biliary AP, with inflammation present in the entire tissue in the former but not latter. Although the extent of this difference in human AP is not known, the perpetuation of excess ethanol consumption will continue to drive the activation of PSCs, contributing to chronicity. The presence of cytokines and inflammatory mediators likely leads to the widespread activation of PSCs manifested as increased α-SMA expression. When neighbouring PACs are damaged and undergo necrosis, they release kallikrein and trypsin [40]. Kallikrein can induce the generation of bradykinin from its precursor, which then affects Ca^2+^ homoeostasis in PSCs (Fig. 1B) [41]. Furthermore, Ca^2+^ signals in PSCs may further perpetuate necrosis and ongoing inflammation in the tissue via mechanisms that involve the production of nitric oxide [42].

Our new results for the first time show that activated PSCs develop remarkable resistance against alcohol metabolites, evidenced by significantly reduced Ca^2+^ responses to EtOH/POA (Fig. 3E–P) and EtOH/POAEE (Fig. 3Q–X) as well as by a marked decrease in cell death caused by EtOH/POA in ahPSCs compared to qhPSCs (Fig. 6F, G). This is particularly important for the pathogenesis of alcoholic pancreatitis. Activated PSCs will no longer be sensitive to alcohol metabolites present in the tissue and may continue to proliferate, activate the remaining quiescent cells, and deposit ECM components.

The mechanism behind the resistance of ahPSCs to alcohol metabolites lies in the enhanced Ca^2+^ handling capacity, compared to qhPSCs, which depends on the expression of Ca^2+^ channels and transporters (Fig. 4). In this study, the most important Ca^2+^-handling proteins were selected for analyses, including generic proteins (such as STIM1, Orai1, IP3Rs and RyRs), and those highlighted in the existing literature related to PSCs and other stellate cells/tissue fibroblasts (such as TRPA1, see below). Our results show that TRPA1 expression drops to very low levels in ahPSCs 48 h and 7 d (Fig. 4I). Pharmacological inhibition as well as silencing of TRPA1 in qhPSCs results in almost identical Ca^2+^ responses to EtOH/POA to those recorded in ahPSCs 48 h (Fig. 5C–F). Even more notably, the TRPA1 blocker protected against EtOH/POA-induced cell death in qhPSCs (Fig. 6F, G). Since altered Ca^2+^ fluxes across the plasma membrane could have an impact on the intracellular Ca^2+^ store content [43], downregulation of TRPA1 could contribute to the apparent decrease in the resting [Ca^2+^]_ER_ of ahPSCs, unmasked by inhibition of SERCA by CPA (Fig. 5G, H). TRPA1 has previously been suggested to be protective against fibrosis in intestinal myofibroblasts, both in vitro and in a mouse model of chronic colitis [44]. Very recently, downregulation of TRPA1 by TGF-β was implicated in the resistance of lung myofibroblasts to cell death [45], although the detailed mechanism has not been investigated. Here, we have identified a remarkable downregulation of TRPA1 in ahPSCs and analysed its pathophysiological consequences in relation to pancreatitis. Given the results of this and previous studies, TRPA1 could play a central role in the regulation of cell death induced by toxic stimuli in a large spectrum of tissue (myo)fibroblasts.

**Fig. 7:**
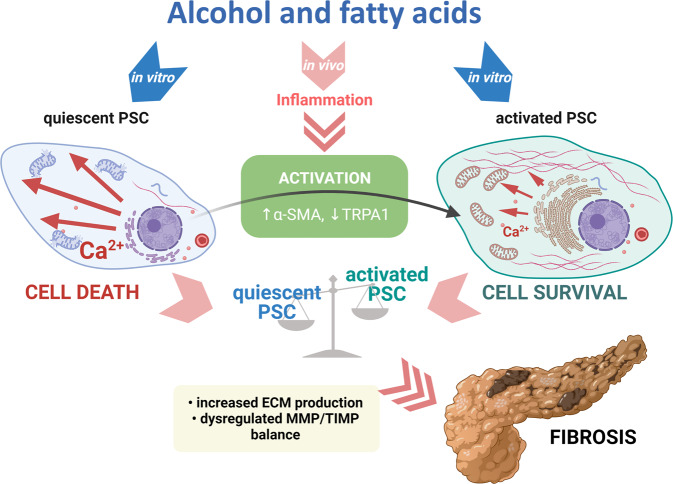
Schematic illustration of PSC activation and its consequences for alcohol metabolite-induced pathology of the pancreas. Full description in the text (created with BioRender.com).

In conclusion, this study sheds new light on the mechanisms underlying alcohol metabolite-induced pancreatic pathology, which involves signalling in PSCs (Fig. 7). Alcohol toxicity is mediated by EtOH, FAs, and FAEEs, which induce damage to PACs. Alcohol metabolites also act on quiescent PSCs inducing [Ca^2+^]_i_ overload, disrupting the mitochondrial potential, and inducing cell death. Alcoholic pancreatitis results in the activation of PSCs throughout the pancreatic tissue. Activated PSCs become resistant to alcohol metabolites owing to their enhanced Ca^2+^ handling capacity compared to the quiescent cells: activated PSCs are protected against excessive [Ca^2+^]_i_ overload by downregulation of TRPA1. Significant resistance of activated PSCs to noxious stimuli (such as alcohol metabolites) might explain why pancreatic fibrosis is a frequent complication of alcoholic pancreatitis. While PACs and quiescent PSCs are prone to cell death, activated PSCs can more easily withstand the ongoing inflammation, repopulate the tissue, and deposit ECM components.

Our study also, for the first time, shows that EtOH in combination with FAs directly elicits Ca^2+^ signals in PSCs and that these signals can be sustained in the presence of extracellular Ca^2+^ (Fig. 1D, E, G, H). Recent work has highlighted the key role of PSCs in inflammation by showing that the SARS-Cov-2 spike protein primarily elicits Ca^2+^ signals in PSCs in situ in the mouse pancreas and that these Ca^2+^ signals subsequently generate Ca^2+^ signals in pancreatic macrophages, most likely via interleukins released from the PSCs [46]. The Ca^2+^ signals shown in the present work to be generated in PSCs by EtOH in combination with FAs could well turn out to be an important element in the initiation of the inflammatory response, which is such a critical part of AP.

## Materials and methods

### Cell culture

Human pancreatic stellate cells (hPSCs, cat. #3830) and dedicated stellate cell complete medium (SteCM) were purchased from ScienCell, Carlsbad, CA, USA. hPSCs were cultured in Stem in T25 flasks at 37 °C, 5% CO_2_ and split once a week as previously described [23]. Immediately after the first passage, multiple frozen hPSC stocks were prepared to be used later for culture revival (approximately every 5–6 weeks). The cells are regularly tested for mycoplasma contamination (by PCR). Activated hPSCs were obtained by culturing hPSCs in the presence of 5 ng/ml TGF-β (Corning, New York, NY, USA) [47] in an “incomplete” SteCM medium i.e. deprived of foetal bovine serum (FBS) and cell growth supplements (CGS) for a set period of time (48 h or 7 days).

### Animal models

Animal experiments were ethically reviewed and carried out in accordance with the Animals (Scientific Procedures) Act 1986 (UK); relevant project licence number: 30/2956. C57BL/6 J mice (males, 6–12 weeks old, 23 ± 3 g weight) were supplied by Charles River Laboratories, UK and housed in institutional animal units (Cardiff University and University of Liverpool, UK) under the following conditions: 12 h light cycle, standard rodent chow diet, free access to water. Minimisation was used as a randomisation strategy to allocate animals to different groups to balance body weight that could influence the results of the experiments [48]. The sample size was estimated and checked based on *Charan and Kantharia, 2013* (E = 15 for alcoholic AP and E = 10 for biliary AP, which was considered adequate) [49]. All animals were included in the analyses. Alcoholic AP was induced by two intraperitoneal injections (1 h apart) of EtOH (1.5 g/kg) and palmitoleic acid (POA, Merck, Darmstadt, Germany; 150 or 300 mg/kg, *n* = 6 for both) in the presence of polyethylene glycol (PEG) 200 (1 g/kg; Merck) [27]. Control mice received saline injections (*n* = 6). Biliary AP was induced by retrograde pancreatic duct infusion of sodium taurocholate (NaTC 1%; NaTC-AP) and taurolithocholic acid 3-sulfate (TLC-S 3 mM; TLC-S-AP), respectively, at 5 µl/min over 10 min by infusion pump [28]; saline injections (Sham) were used as experimental controls (*n* = 3) for both models. Mice were sacrificed according to Schedule 1 of Animals (Scientific Procedures) Act 1986, dissected and the pancreas was removed. Pancreatic tissue was fixed in 10% formalin (for 48 h), dehydrated by subsequent incubations in solutions with increasing EtOH content: 50, 70, 96 and 100% and then in xylene and liquid paraffine. The processed samples were embedded in paraffin and cut into 5 µm thick sections for further experimental procedures.

### Cytosolic Ca^2+^ and mitochondrial potential imaging

For calcium imaging, hPSCs cultured on Ø32 mm round coverslips were loaded with 2 μM Fluo-4 AM (Thermo Fisher Scientific, Waltham, MA, USA) for 30 min at 37 °C; whereas for mitochondrial potential measurements, cells were loaded with 25/100 nM tetramethylrhodamine methyl ester (TMRM, Thermo Fisher Scientific). Fluo-4 AM loading and all real-time imagining experiments were carried out in NaHEPES buffer (140 mM NaCl, 4.7 mM KCl, 10 mM HEPES, 1 mM MgCl_2_, 10 mM glucose; pH 7.2; supplemented with 1 mM CaCl_2_) [23]. After loading, cells were washed with fresh NaHEPES buffer and used for experiments at room temperature (RT) in a flow chamber perfused with NaHEPES-based extracellular solution. All measurements of Ca^2+^/mitochondrial potential were performed on the ZEISS LSM 880 confocal microscope (Carl Zeiss AG, Oberkochen, Germany), 40× oil objective. Fluo-4-loaded cells were excited with 488 nm laser light, and collected fluorescence was set to 593–630 nm. For the cells loaded with TMRM, the excitation wavelength was 561 nm and the collected emission was set in the range of 579–721 nm. Time series were recorded at 256 × 256 pixel resolution, two consecutive frames were averaged, and the interval between images was 2 s. Fluorescence signals were plotted as F/F_0_, where F_0_ was an averaged value calculated from the initial ten baseline images. The area under the curve was calculated for each trace. The average values obtained for each experimental group were presented as bar charts (mean ± SEM) with all individual points shown. For TRPA1 inhibition experiments, a 5-min preincubation with HC-030031 (Merck) was applied immediately before calcium recording. The inhibitor was present throughout the entire experiment. For all Ca^2+^ experiments, a pre-defined exclusion criterion was used: spontaneous activity higher than 3×(baseline values) before the application of any stimulus.

### RNA isolation, reverse transcription and real-time PCR

RNA was isolated from cells cultured on 6-well plates using the Total RNA Mini Kit (A&A Biotechnology, Gdynia, Poland) according to the manufacturer’s protocol. RNA concentration was measured with an ND-1000 spectrophotometer (Thermo Fisher Scientific). Equal amounts of total RNA (1 µg) were taken for cDNA synthesis using the High Capacity cDNA Reverse Transcription Kit (Thermo Fisher Scientific) and following the manufacturer’s protocol. For real-time PCR assays, SYBR Green PCR Mix (Thermo Fisher Scientific) was used. Specific gene primer pairs (Genomed, Warsaw, Poland) are listed in Table 1. The real-time PCR reaction was performed using a 7500 Fast Real-Time PCR thermocycler (Applied Biosystems, Waltham, MA, USA). Reaction conditions were as follows: 50 °C (20 sec), 95 °C (5 min); then 40 cycles of 95 °C (15 sec) and 60 °C (1 min) each. Relative gene expression was calculated as the cycle threshold value (Ct) normalised to glyceraldehyde-3-phosphate dehydrogenase (*GAPDH*) using the 2(–∆∆Ct) method [50].

**Table 1:** List of primer sequences used in real-time PCR experiments.

Target gene	Forward primer	Reverse primer
*ACTA2*		
*(actin alpha 2, smooth muscle)*	ACTGCCTTGGTGTGTGACAA	CACCATCACCCCCTGATGTC
*VIM*		
*(vimentin)*	AAATGGCTCGTCACCTTCGT	AGAAATCCTGCTCTCCTCGC
*DES*		
*(desmin)*	CCAACAAGAACAACGACGCC	ATCAGGGAATCGTTAGTGCCC
*FN*		
*(fibronectin)*	TGTGGTTGCCTTGCACGAT	GCTTGTGGGTGTGACCTGAGT
*STIM1*		
*(stromal interaction molecule 1)*	GCAGCAGAGTTTTGCCGAAT	TGTGGATGTTACGGACTGCC
*ORAI1*		
*(calcium release-activated calcium channel protein 1)*	GCCCTTCGGCCTGATCTTTA	TCCTGTAAGCGGGCAAACTC
*TRPC3*		
*(transient receptor potential cation channel, subfamily C, member 3)*	CTCAGACAGGTTCGAAGGCA	ATCATTCCAAGAACCCAGACCA
*TRPC6*		
*(transient receptor potential cation channel, subfamily C, member 6)*	ATGGCGGTCAAGTTCCTTGT	ATCTTCCCCATCTTGCTGCAT
*TRPA1*		
*(transient receptor potential cation channel, subfamily A, member 1)*	TAATGGGAAAGCCACCCCTC	TTCCCTTCTCCACTGGGTCTA
*PMCA4*		
*(transient receptor potential cation channel, subfamily C, member 3)*	GGGATGCACTGACCCAGATT	CCAGACAGACCTTCCACAGG
*ITPR1*		
*(inositol 1,4,5-trisphosphate receptor type 1)*	GGAGACAGCGTGGTCATAGG	CCTCATTGCAGCCTGGGTTA
*ITPR2*		
*(inositol 1,4,5-trisphosphate receptor type 2)*	TTCATCATGACCCATGCCGT	TCAGGATTAAGCTCTGCAGCTA
*ITPR3*		
*(inositol 1,4,5-trisphosphate receptor type 3)*	AGCAATTACGAGCTCAGCGA	ACGTCTCCCCCTTTCAACAC
*RYR1*		
*(ryanodine receptor type 1)*	ATGCCACTCAAGCTCCTCAC	ATGCCCCAGAAGAGTTTCCG
*RYR2*		
*(ryanodine receptor type 2)*	GCATAGACCGTTTGCACGTC	AATTAGAGCCGCCAGCAACT
*RYR3*		
*(ryanodine receptor type 3)*	GAGGAGAGATGCCCCACAAC	ATGCTGTCATACTGCCTCCG
*GAPDH*		
*(glyceraldehyde-3-phosphate dehydrogenase)*	GAAGGTGAAGGTCGGAGT	GAAGATGGTGATGGGATTTC

### Protein isolation and Western blotting

PSCs were lysed in RIPA buffer (Merck) supplemented with a protein inhibitor cocktail (Sigma) and the protein concentration was measured using a bicinchoninic acid assay (Sigma). 20 µg protein samples were separated by SDS-PAGE and then transferred onto polyvinylidene fluoride membranes (wet transfer, 1 h, 30 V) in an XCell SureLock^®^ Blot Module (Thermo Fisher Scientific). To prevent non-specific binding, membranes were incubated in 5% (w/v) skimmed milk in TBST (0.05% (v/v) Tween 20 in Tris-buffered saline). The membranes were then incubated overnight at 4 °C with primary antibodies (A2547, V9131, Merck) in antibody buffer containing 1% (w/v) milk in TBST, washed with TBST three times and incubated with peroxidase-labelled secondary antibody (PI-2000, Vector Laboratories) in the same antibody buffer. Detection was performed with the chemiluminescence HRP substrate (Merck) in the Bio-Rad ChemiDoc imager.

### Histopathology and histological scoring

Haematoxylin/eosin (H/E) staining was carried out as previously described [28, 51]. Briefly, formalin-fixed paraffin-embedded tissue sections (5 μm) were deparaffinised by heating in a dry oven (15 min, 65 °C) followed by two consecutive 10 min incubations in xylene. Rehydration was achieved by subsequent washes in EtOH solutions with increasing content of deionized H_2_O (dH_2_O): twice in 100% EtOH, then once in 96, 70 and 50% EtOH, and finally in dH_2_O. Sections were stained in Mayer’s haematoxylin solution (Merck) for 12 min, and then washed in running tap water (15 min). The sections were then incubated in 0.5% eosin Y solution with phloxine (Merck) (90 s). Subsequently, the sections were dehydrated in solutions with an increasing EtOH content: 70, 96 and 100% followed by washing in xylene. The slides were sealed with Histofluid mounting medium (Paul Marienfeld, Lauda-Königshofen, Germany). Microscopic evaluation of H/E samples was carried out by assessing 10 random fields of view in a ‘blind’ manner. The severity of each parameter—oedema, inflammatory cell infiltration, and acinar cell necrosis—was scored using a four-level grade [28].

### Immunocytofluorescence

hPSCs were seeded on Ø13 mm round glass coverslips and incubated for 24 h in an 'incomplete' SteCM with ethanol (Chempur, Piekary Slaskie, Poland) and POA or POEE. After washing with phosphate-buffered saline (PBS), cells were fixed in methanol (Avantor Performance Materials, Gliwice, Poland) for 20 min at −20 °C. Methanol was removed by three washes in PBS (5 min each) and blocking of non-specific binding sites was achieved by incubation in 2% BSA in PBS for 30 min (shaker, RT). Primary mouse antibody anti-αSMA (ab7817, Abcam, Cambridge, UK) and/or rabbit anti-TRPA1 (NB110-40763, Novus Biologicals, Biotechne) were applied in 1:300 dilution in 1% BSA for 1 h at RT. The fixed cells were then washed three times in PBS (5 min each) and incubated with the secondary goat anti-mouse antibody Alexa Fluor 488 and/or goat anti-rabbit antibody Alexa Fluor 635 (A11001, A31576, Thermo Fisher Scientific) (1:500 dilution, 30 min, RT). After washing in PBS (four times, 5 min each) and rinsing in dH_2_O, the slides were sealed with ProLong Diamond Antifade Mountant with DAPI (Thermo Fisher Scientific). Imaging was done with a ZEISS LSM 880 confocal microscope.

### Immunohistofluorescence

For immunohistofluorescence (IHF), a previously established protocol was used with modifications [23]. Before the staining, formalin-fixed paraffin-embedded tissue sections (5 μm) were deparaffinised by heating in a dry oven (15 min, 65 °C) followed by two consecutive 10 min incubations in xylene. The sections were rehydrated by subsequent washes in ethanol solutions as described above for H/E staining. The sections were then incubated in 50 mM NH_4_Cl (Merck) in dH_2_O (30 min). Subsequently, heat-induced antigen retrieval was carried out in TAE buffer (Merck) (pH 8.1) in an autoclave (20 min, 120 °C) and then the sections were allowed to cool at RT for 30 min before permeabilisation in 0.4% Triton X-100 (Avantor Performance Materials) in dH_2_O (10 min). Later, the sections were washed three times (5 min each) in 0.1% Tween 20 in dH_2_O ('washing solution'). In order to quench autofluorescence of the pancreas, the sections were incubated in 0.2% Sudan Black B (Merck) in 70% EtOH for 20 min and washed four times (5 min each) in a washing solution. Blocking of non-specific binding sites was achieved by 1 h incubation in 1% BSA in PBS with 0.1% Tween 20. The sections were then incubated with anti-α SMA antibody (ab7817, Abcam, Cambridge, UK) in 1:300 dilution in the blocking buffer overnight at 4 °C in a humidity chamber. The following day, the sections were washed four times (5 min each) in the washing solution and incubated for 1 h at RT with the secondary goat anti-mouse antibody Alexa Fluor 488 (A11001, Thermo Fisher Scientific) (1:500 dilution in blocking buffer, 30 min, RT). After incubation, the sections were washed four times (5 min each) in the washing solution, embedded in ProLong Diamond Antifade Mountant with DAPI and imaged immediately using the ZEISS LSM 880 confocal microscope. The slides were then stored at 4 °C. QuPath software was used for quantitative analysis of the staining [52].

### Cell death assay

hPSCs were plated on Ø32 mm glass coverslips and grown for 24 h in SteCM or activated with TGF-beta (5 ng/ml) in an incomplete medium (SteCM without FBS/CGS) at 37 °C. The medium was then replaced with NaHEPES buffer containing EtOH and POA of a given concentration; and the hPSCs were incubated for 30 min at 37 °C. 15 min before the end of the incubation, annexin V-FITC (1 µg/mL), propidium iodide (PI, 1 μg/mL) and Hoechst-33342 (5 μg/mL) (all from Thermo Fisher Scientific) were added. Green annexin V-FITC staining was used to detect apoptotic cells, PI stained necrotic cells, and Hoechst stained nuclei. Multiple random images (15) per treatment group were taken using the ZEISS LSM 880 confocal microscope. Apoptotic, necrotic, and live cells were counted in images of each treatment group, and the results were presented as a percentage of all cells.

### RNA interference-mediated gene silencing

hPSCs were cultured up to 50% confluence on glass coverslips. The cells were then transfected with 20 nM siRNA targeted against human *TRPA1* (Thermo Fisher Scientific) pre-mixed with RNAiMAX Lipofectamine (*Silencer*^TM^ Select, Thermo Fisher Scientific), according to the manufacturer’s protocol. The siRNA-lipid complex was prepared in Opti-MEM medium (Gibco), incubated for 5 min, and added to the cells. After 72 h of transfection, cells were used for cytosolic Ca^2+^ imaging and immunocytofluorescence staining. As a negative control, mock siRNA was used (*Silencer*^TM^ Select Negative Control No. 1 siRNA, Thermo Fisher Scientific).

### Statistical analysis

For cell death assays, three independent experiments were carried out for each treatment group. The average values and standard errors of the mean were calculated and the results were presented as bar charts. For quantitative analysis of Ca^2+^ responses or measurements of the mitochondrial potential, areas under individual traces (over baseline) were calculated and then averaged and presented as bar charts with standard errors of the mean and individual data points. Statistical analysis was performed with GraphPad software. For cell-based experiments, no statistical tools were applied to pre-estimate sample size. In animal models, group size was estimated based on Charan and Kantharia, 2013 [49]. Normality of the data was assessed with the Shapiro–Wilk test and homogeneity of variances with the Levene test. In cases when the date did not pass the normality test, a non-parametric statistical test was applied. The significance threshold was set at the *p* value = 0.05 and, when applicable, was adjusted for multiple comparisons. Statistical tests used for data analysis are indicated in each figure legend. Where applicable, *N* indicates the number of independent experimental replicates, while *n* indicates individual cells.

## Supplementary information

Supplementary Figure 1

Checklist

Original Data File

## Data Availability

All relevant data are included in the article. Raw data from individual experiments will be made available upon request.
