# Supplementary material for: Activation of pancreatic stellate cells attenuates intracellular Ca2+ signals due to downregulation of TRPA1 and protects against cell death induced by alcohol metabolites
Source: Cell Death Dis. 2022 Aug 29;13(8):744. doi: 10.1038/s41419-022-05186-w (PMC9421659; doi:10.1038/s41419-022-05186-w)
Supplement: Supplementary file 1 — Supplementary Figure 1 [file 41419_2022_5186_MOESM1_ESM.docx]

**Supplementary Figure 1.**

**Uncropped images of Western blots for α-SMA and vinculin.**

Membrane was cut prior to incubation with primary antibodies at 75 kDa mark.

**(A)** Western blot for α-SMA merged with colorimetric image of the protein marker.

**(B)** Western blot for vinculin (loading control) merged with colorimetric image of the protein marker. Selected molecular weights were marked with arrows.
