# Supplementary figures and images for: Activation of pancreatic stellate cells attenuates intracellular Ca2+ signals due to downregulation of TRPA1 and protects against cell death induced by alcohol metabolites
Source: Cell Death Dis. 2022 Aug 29;13(8):744. doi: 10.1038/s41419-022-05186-w (PMC9421659; doi:10.1038/s41419-022-05186-w)

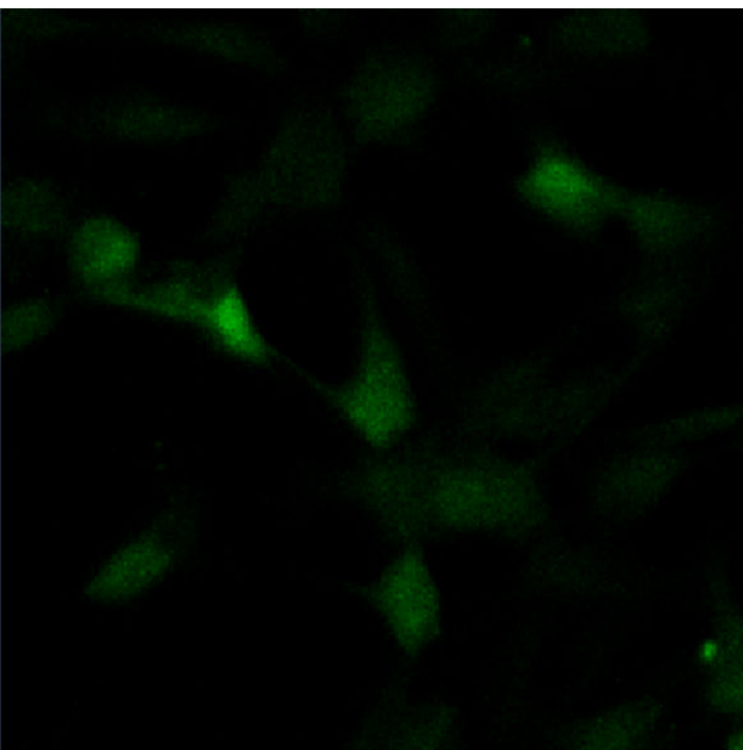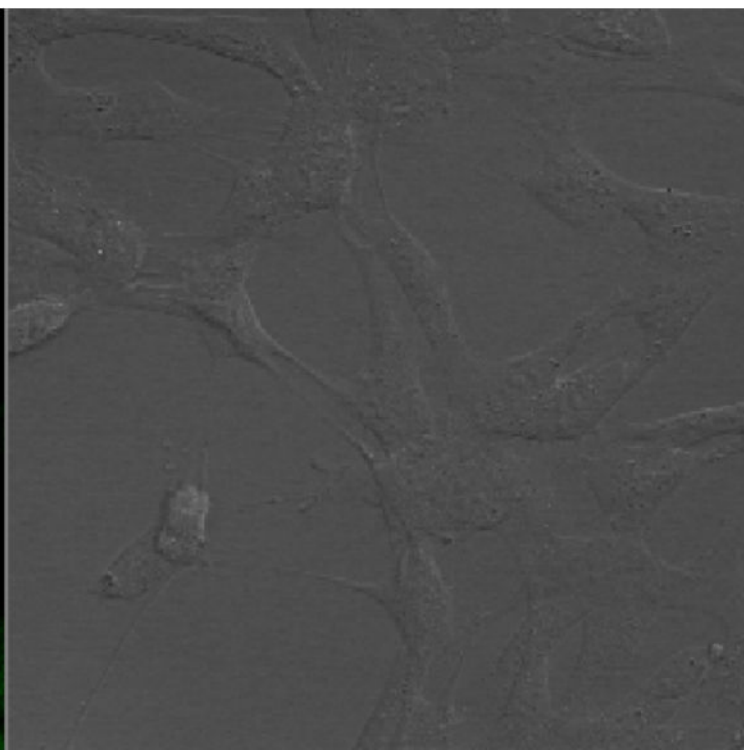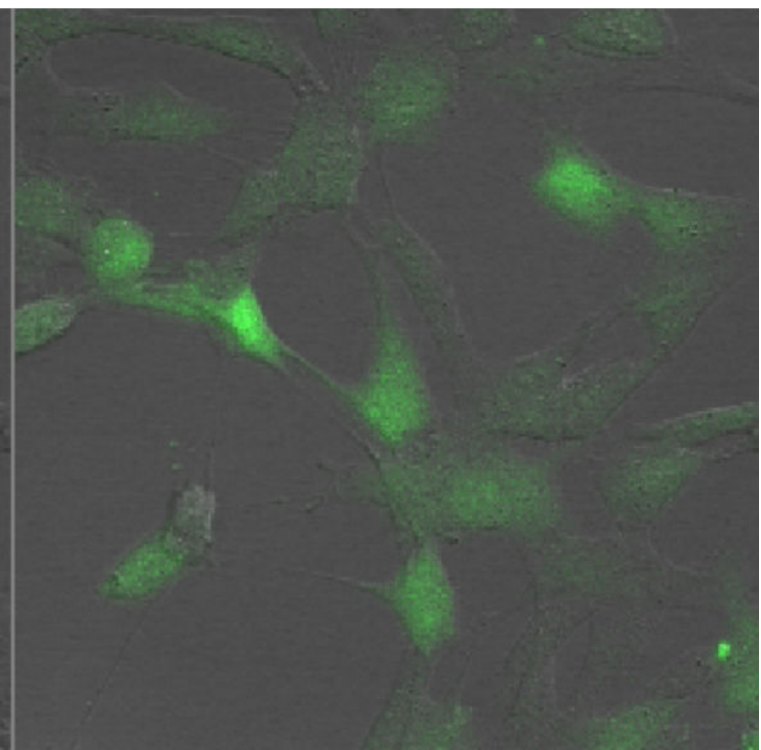

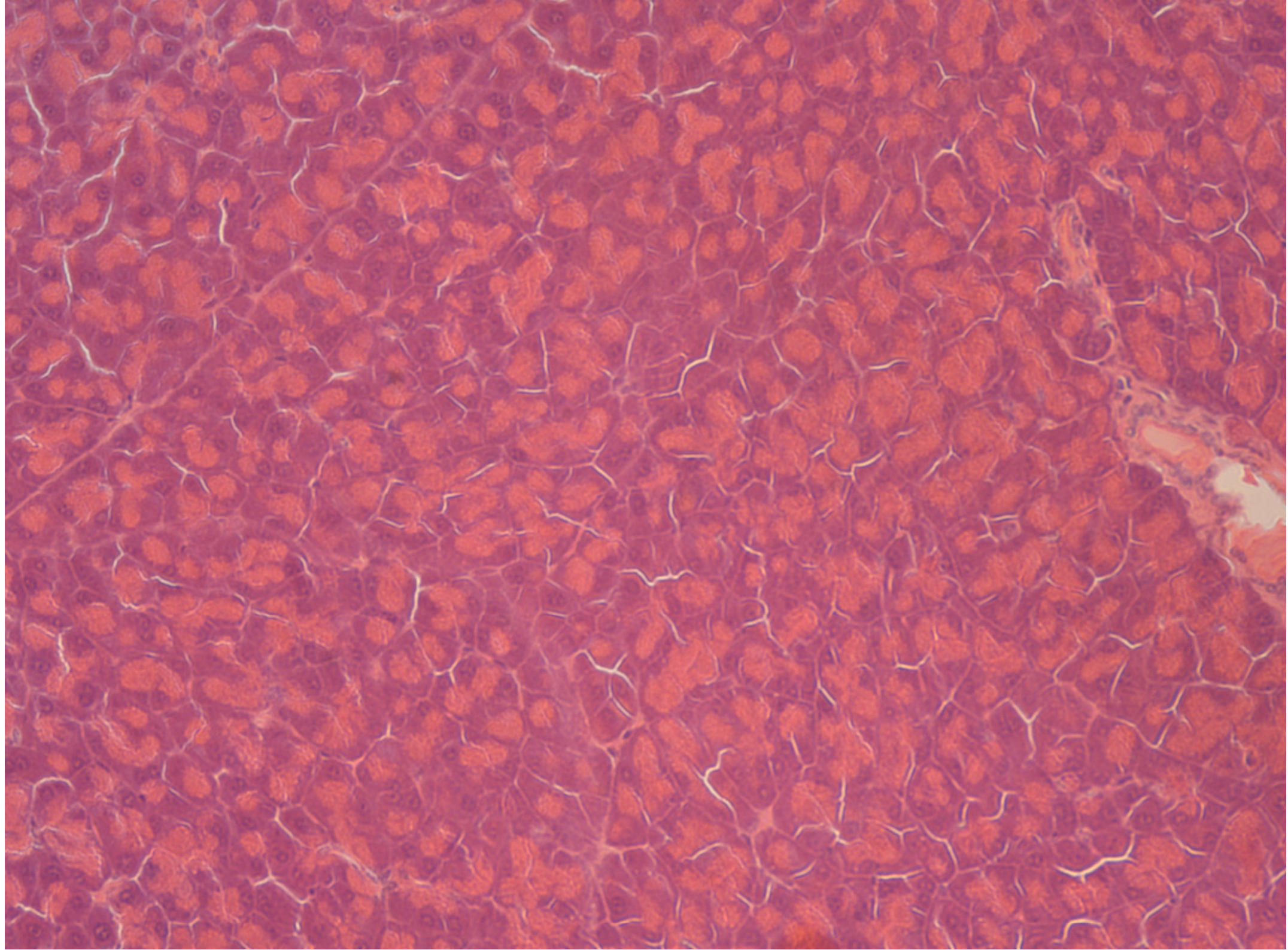

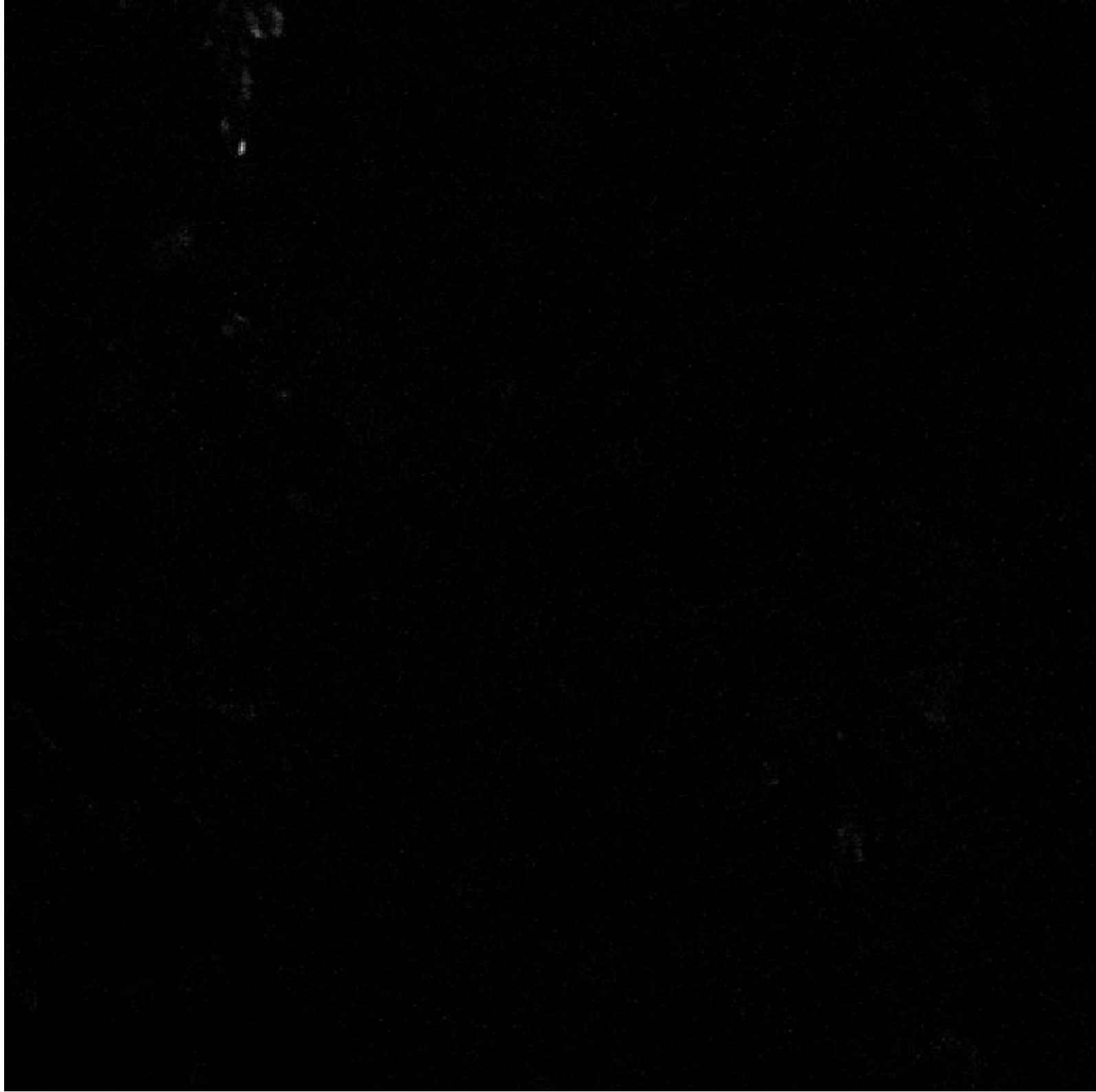

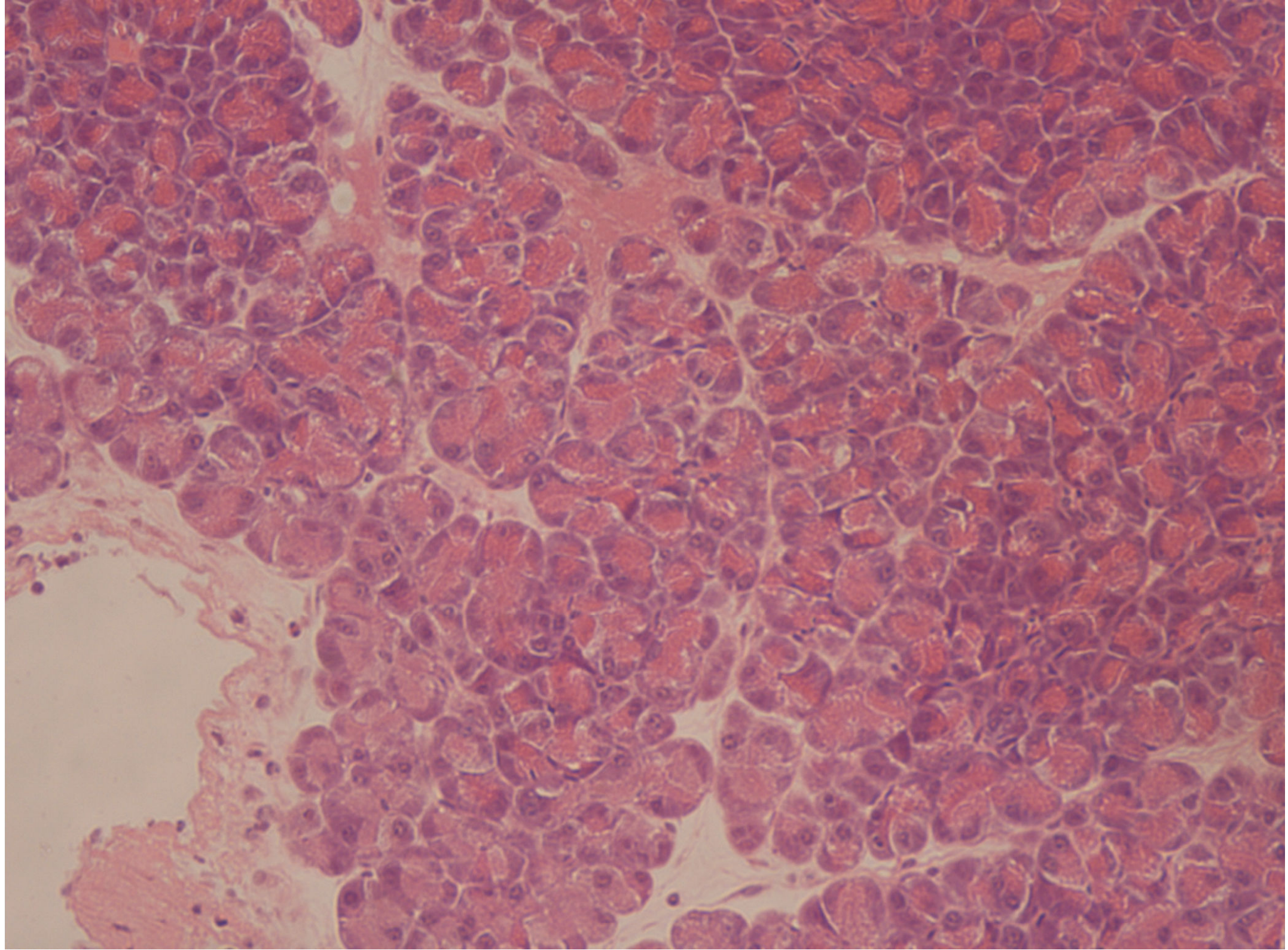

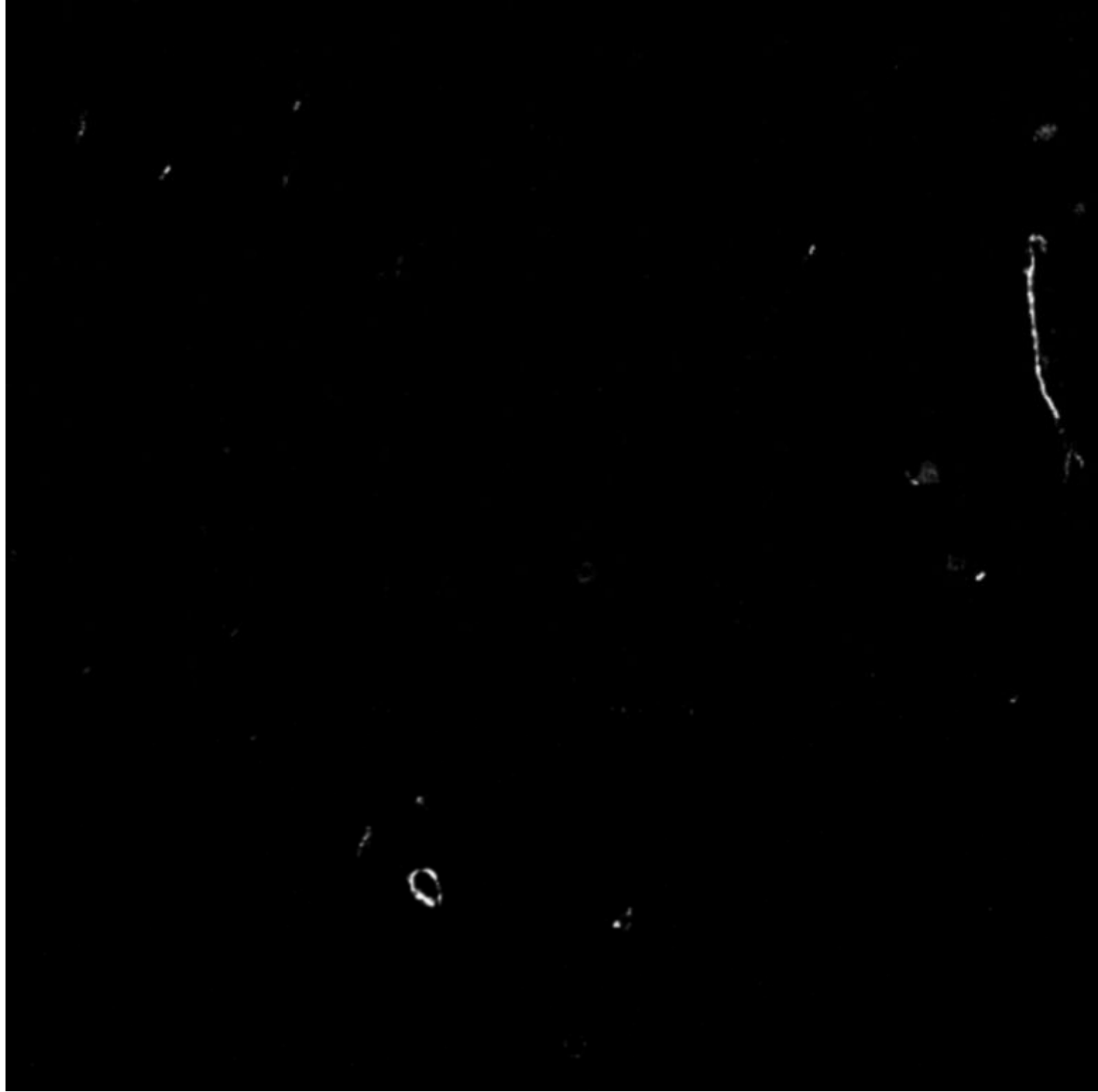

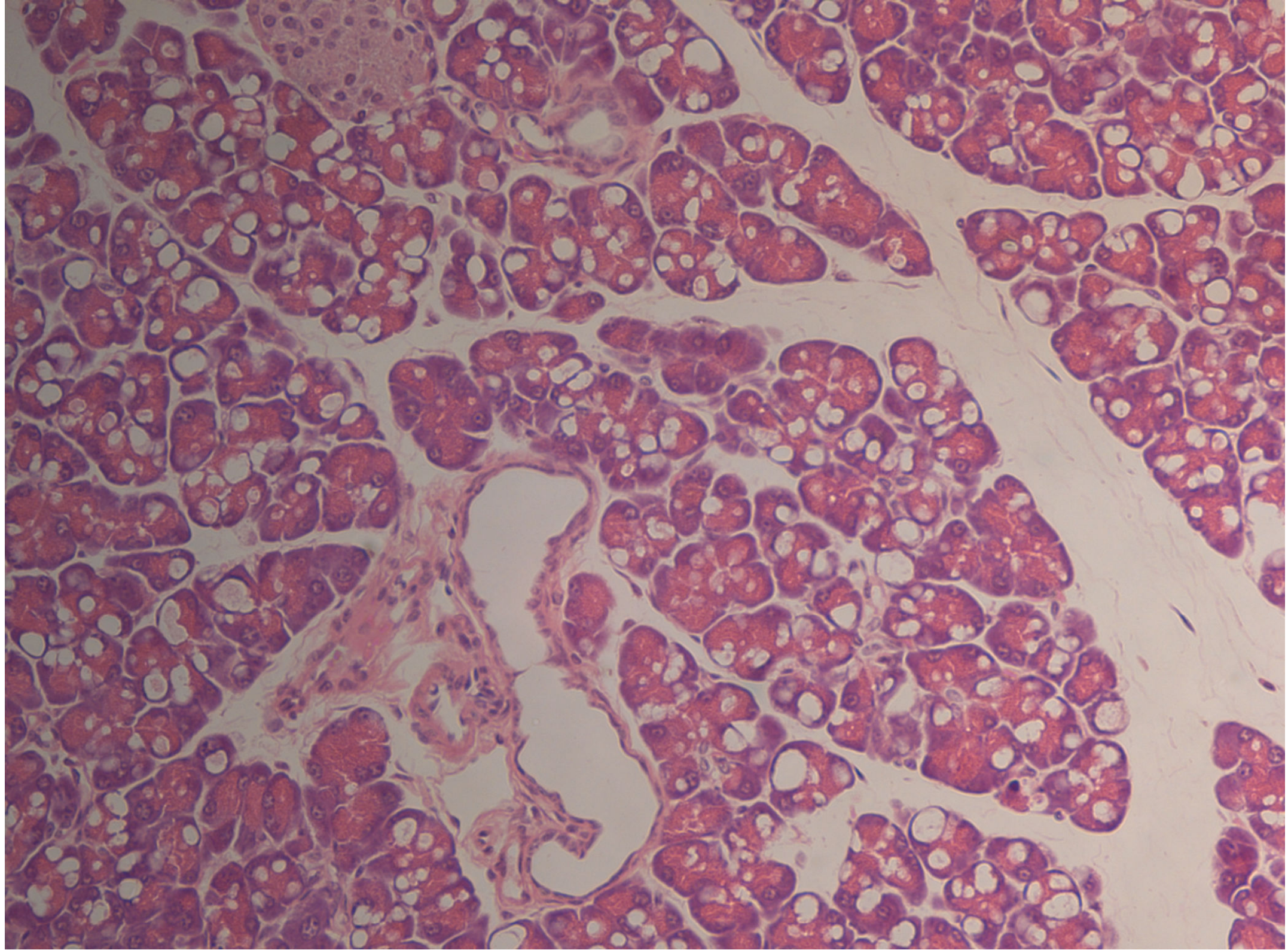

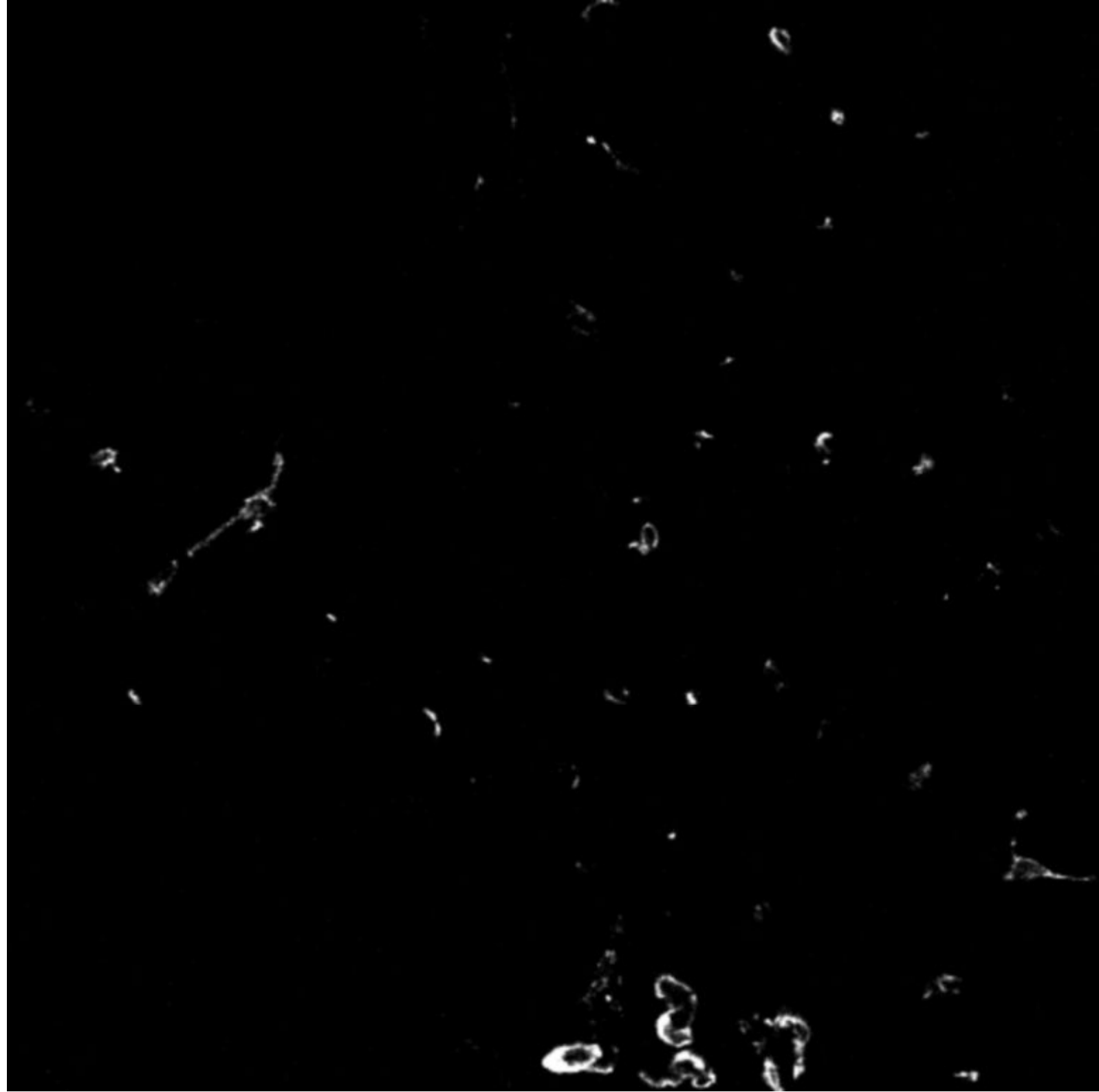

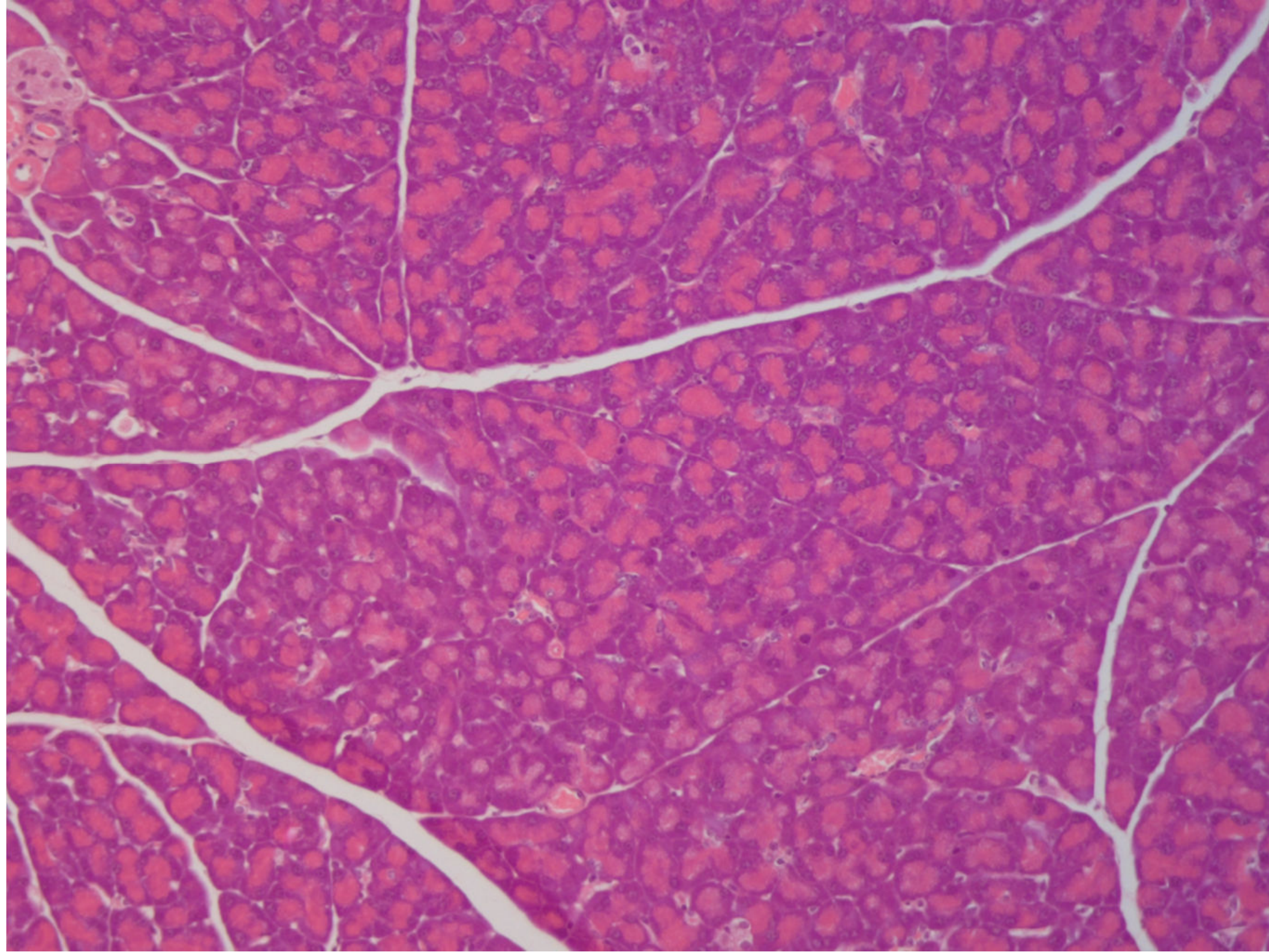

2

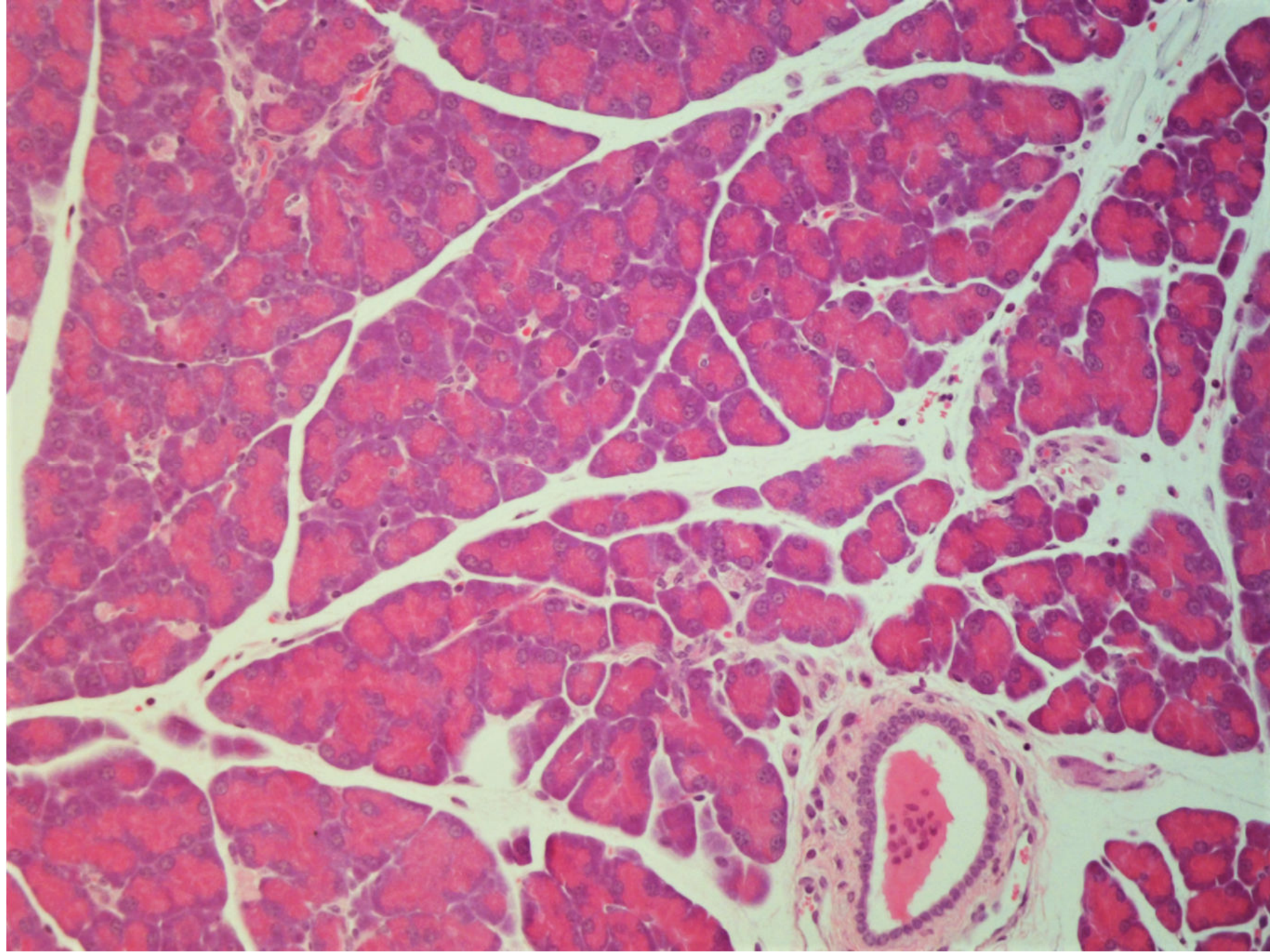

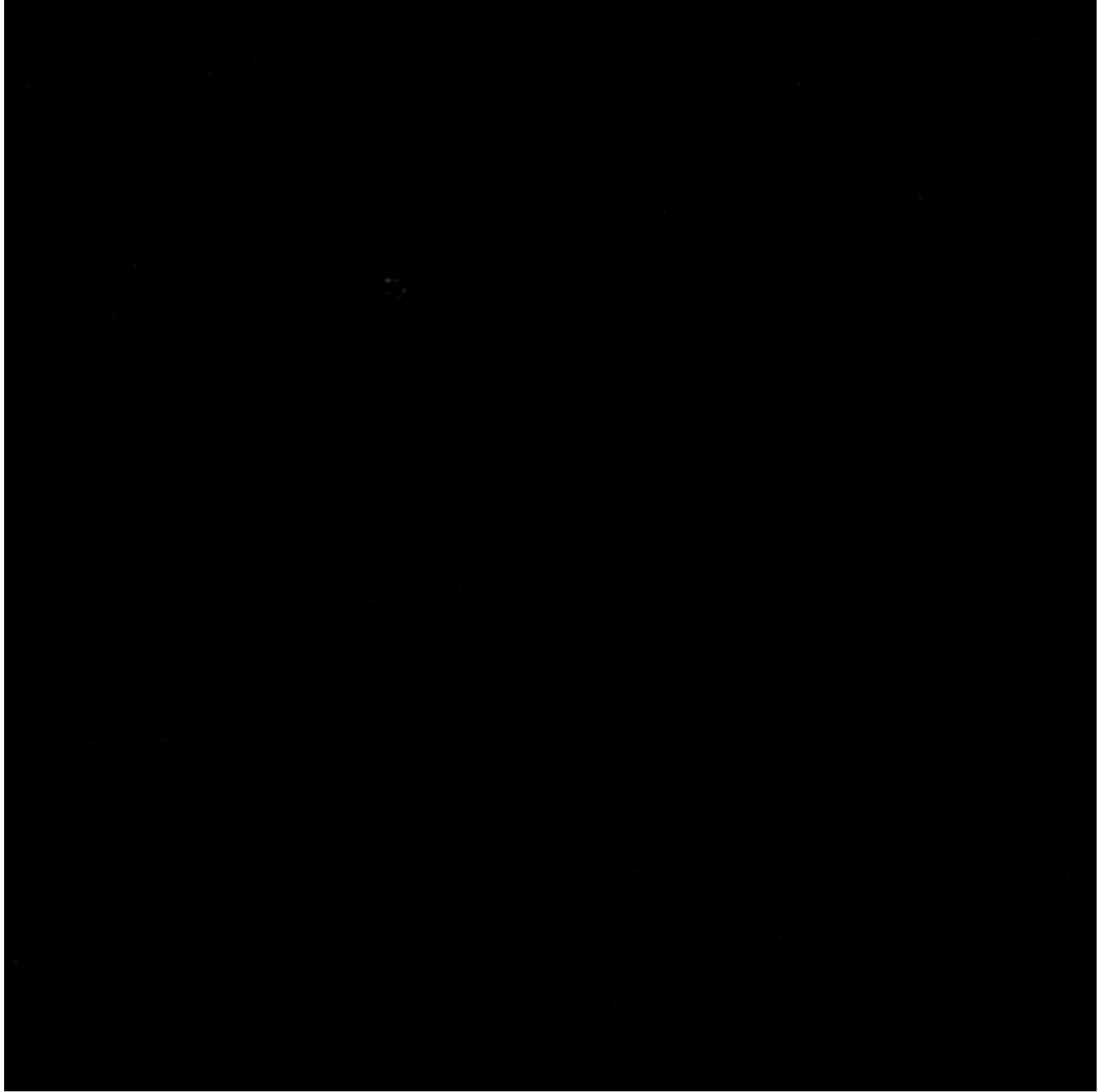

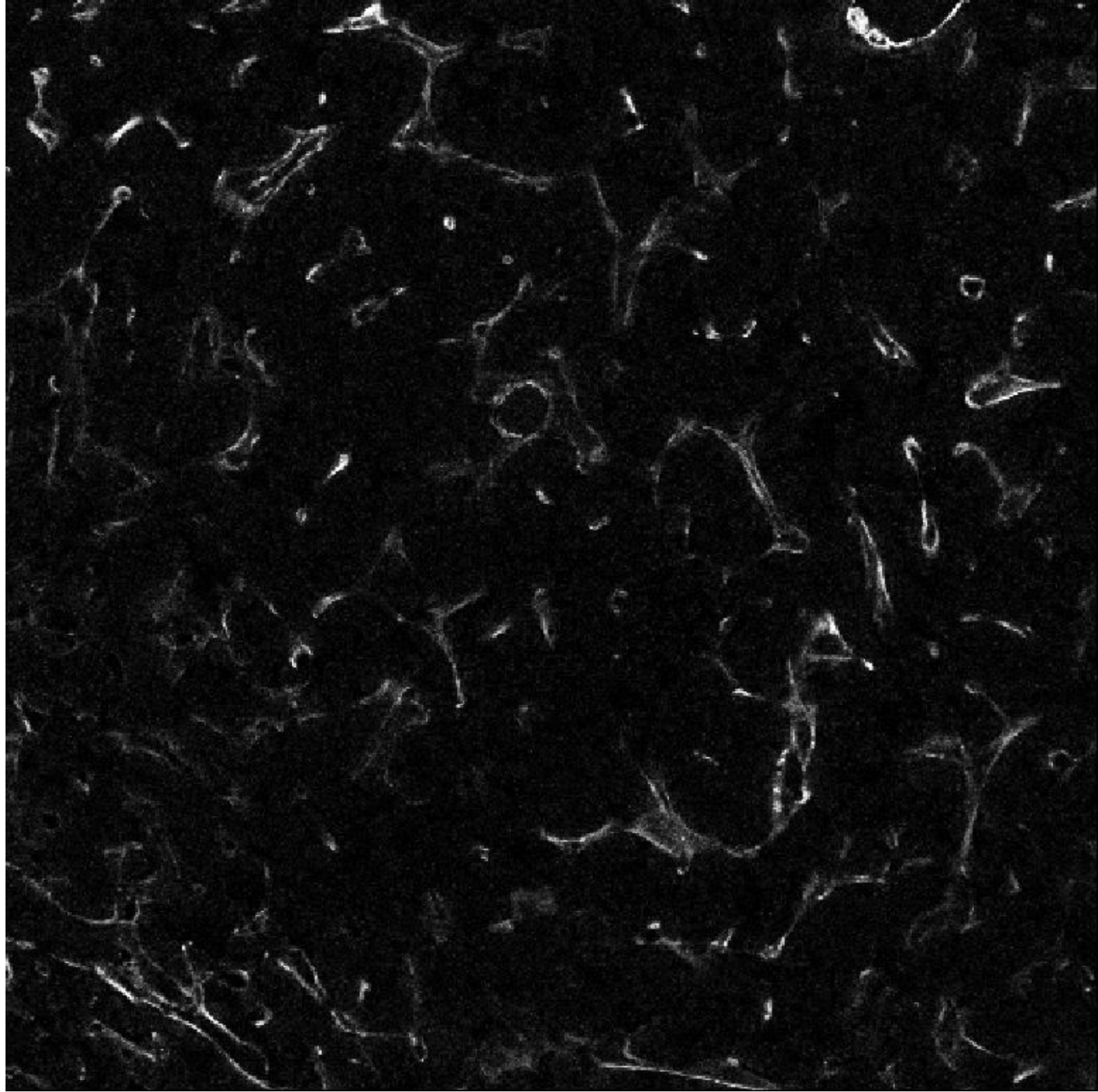

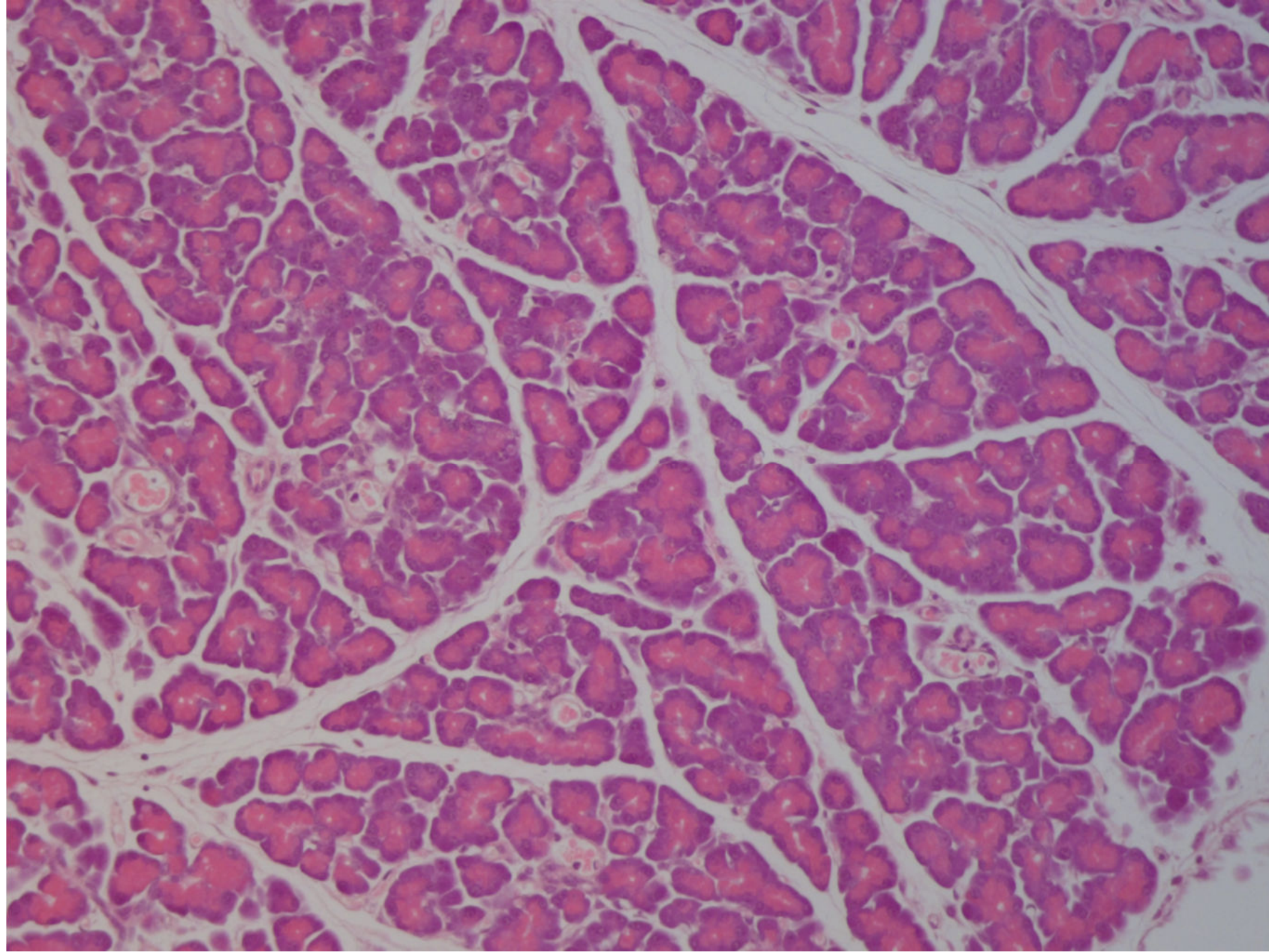

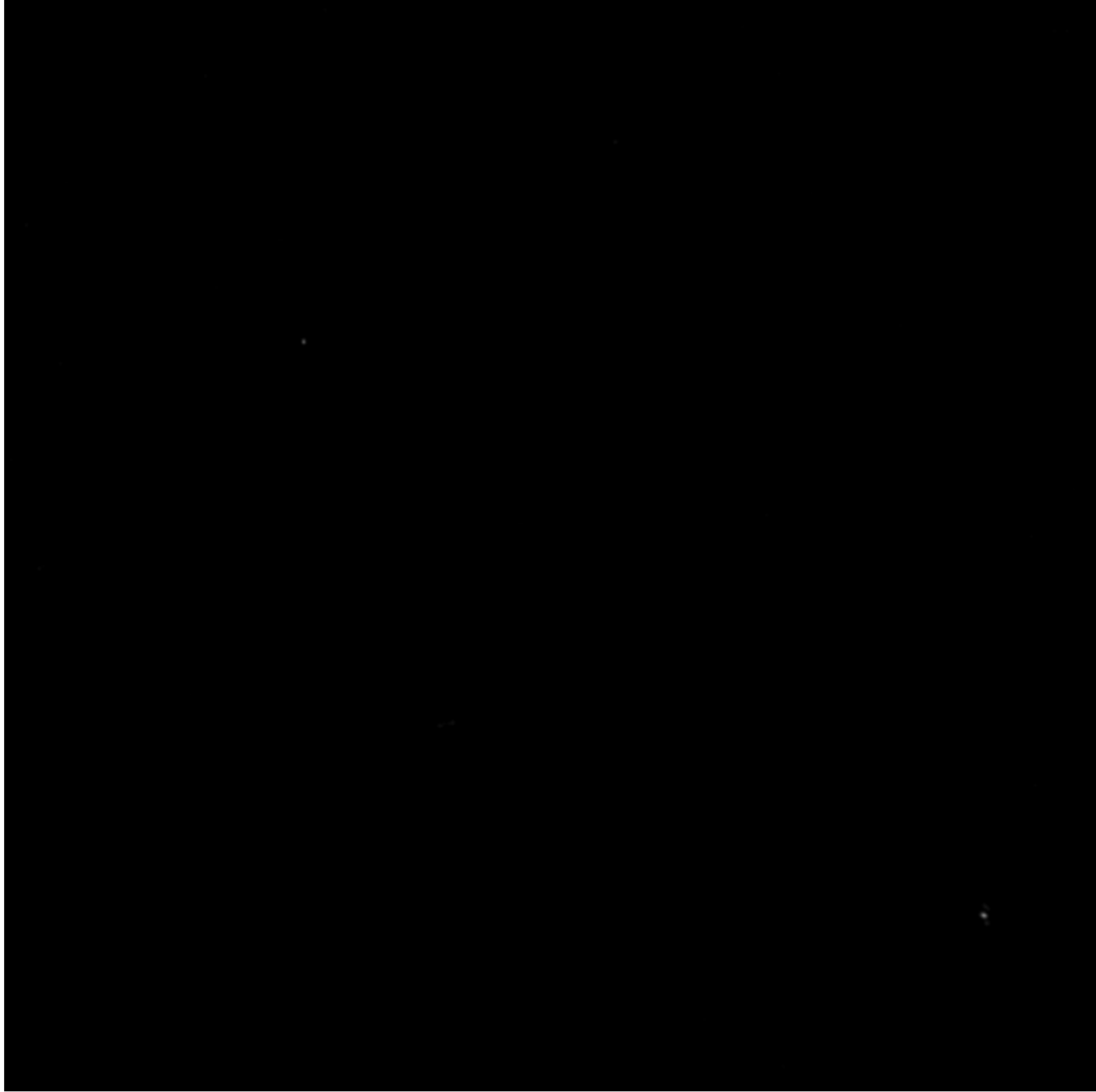

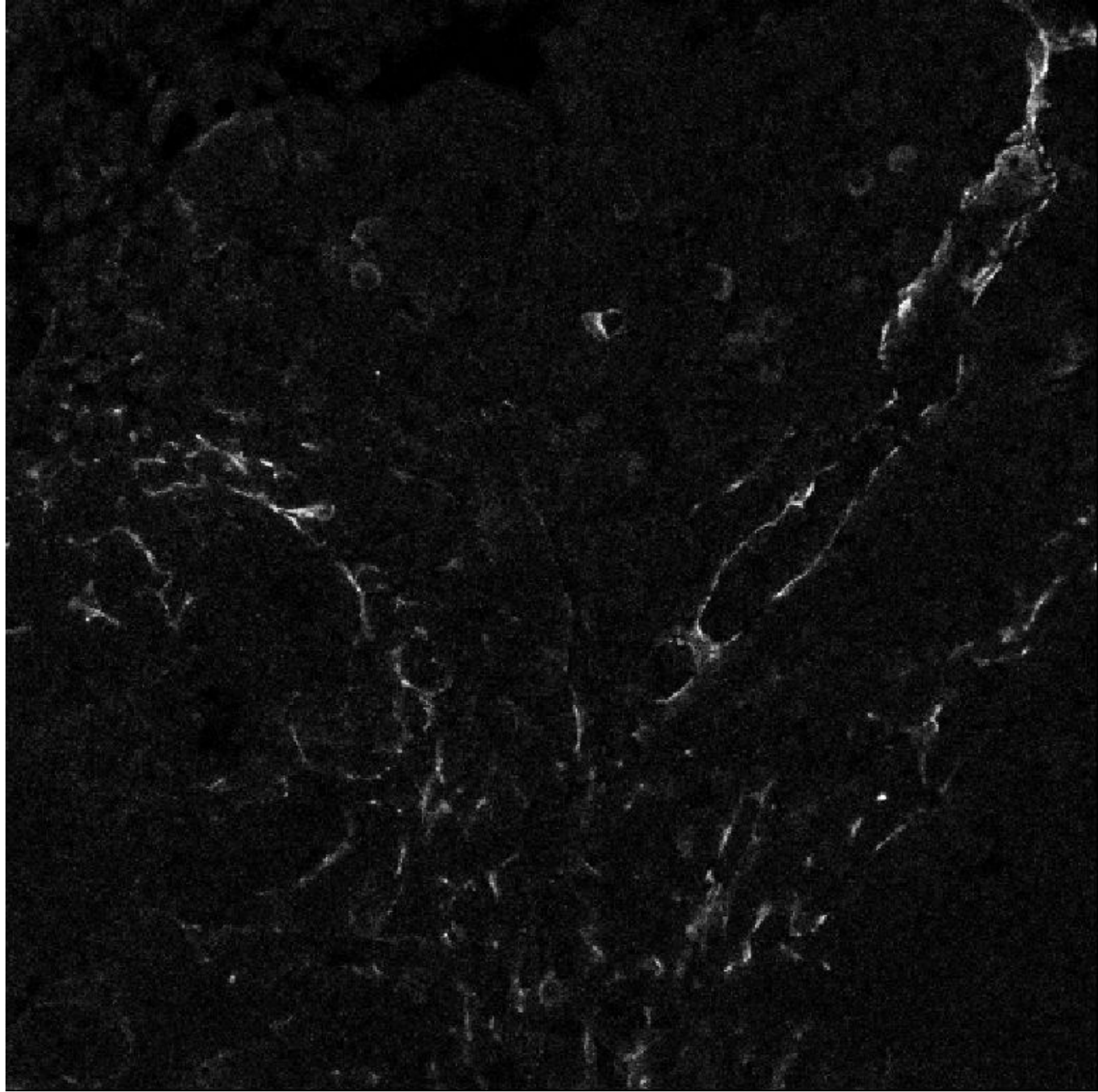

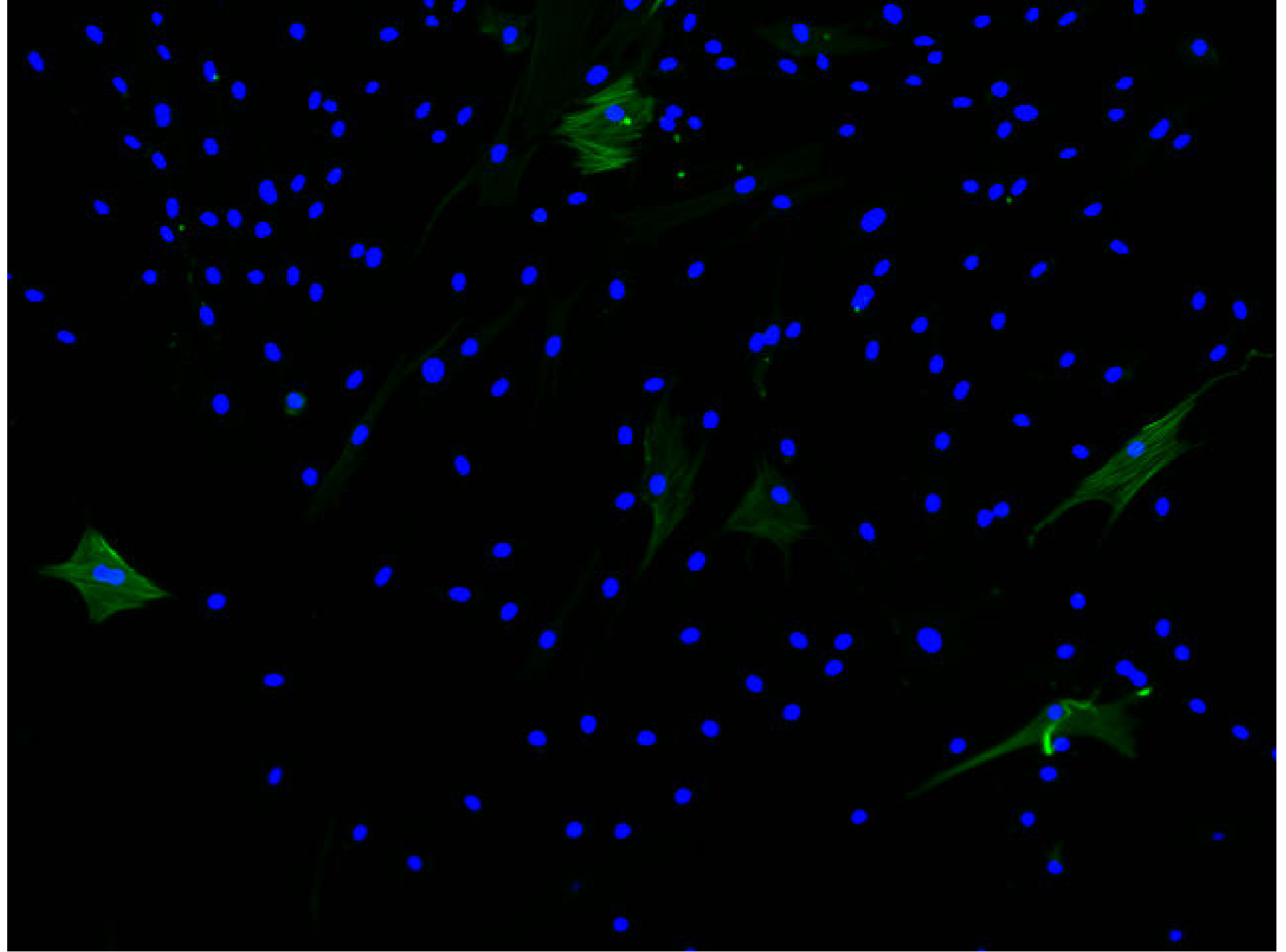

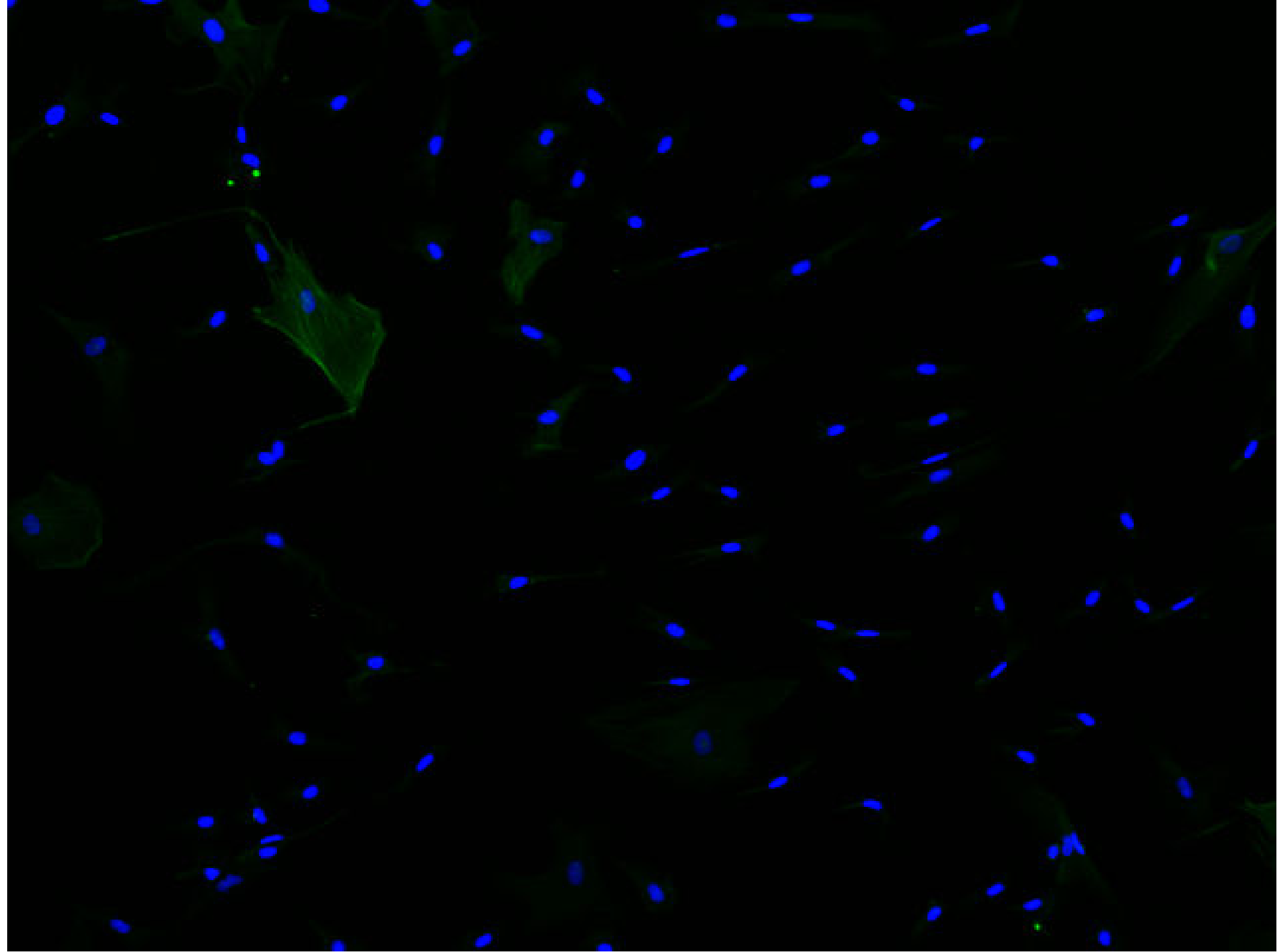

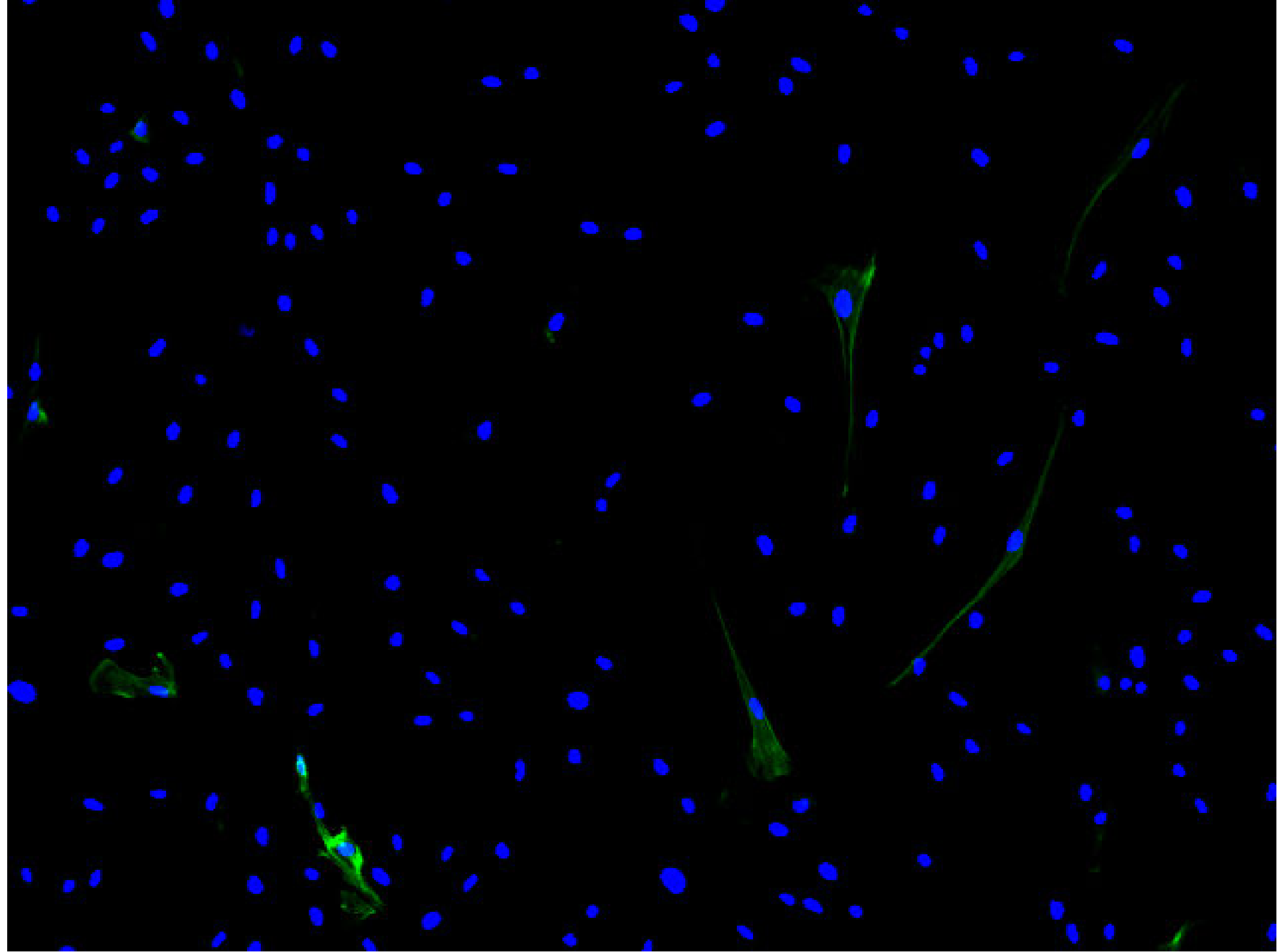

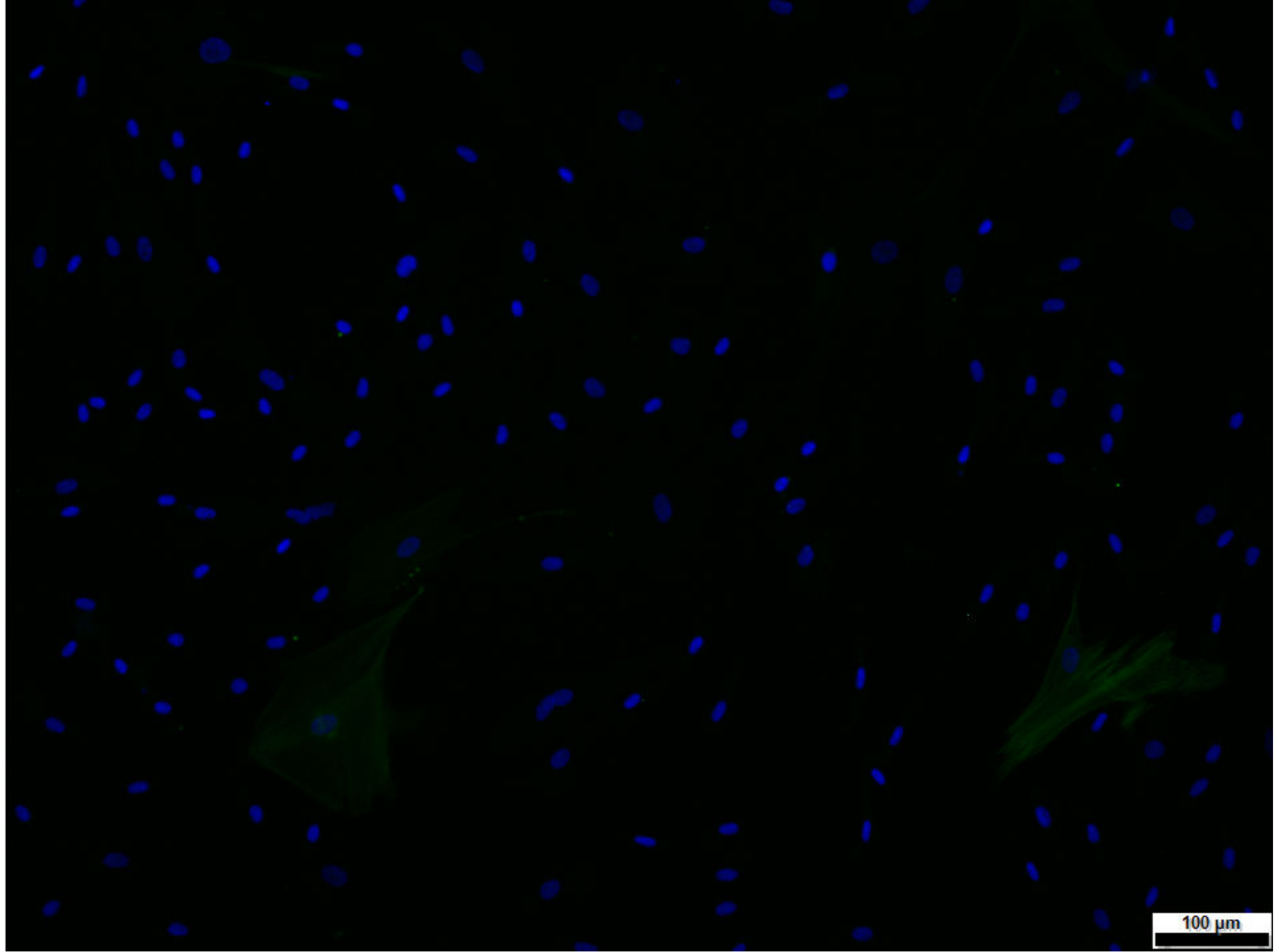

100  $\mu\text{m}$

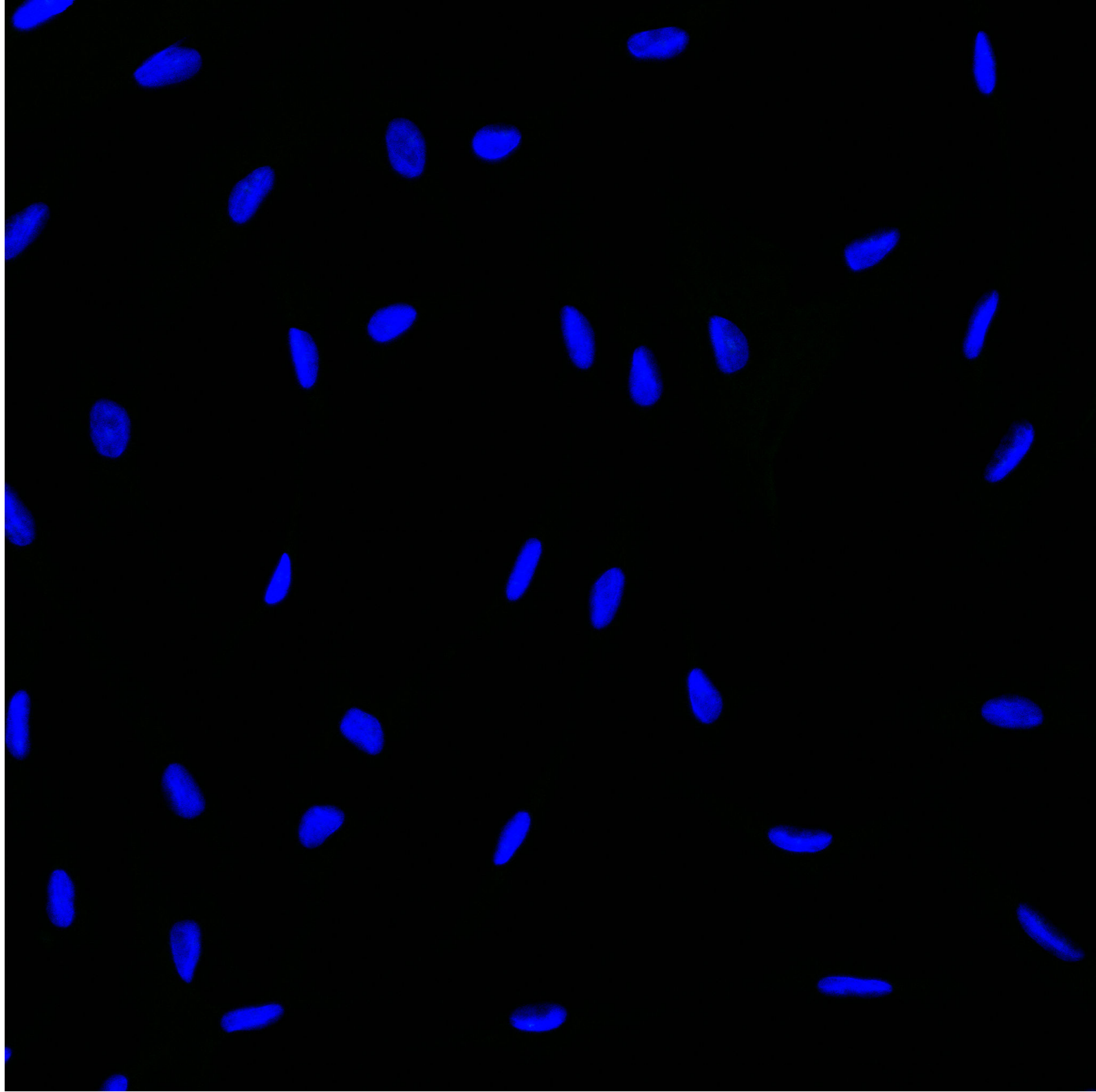

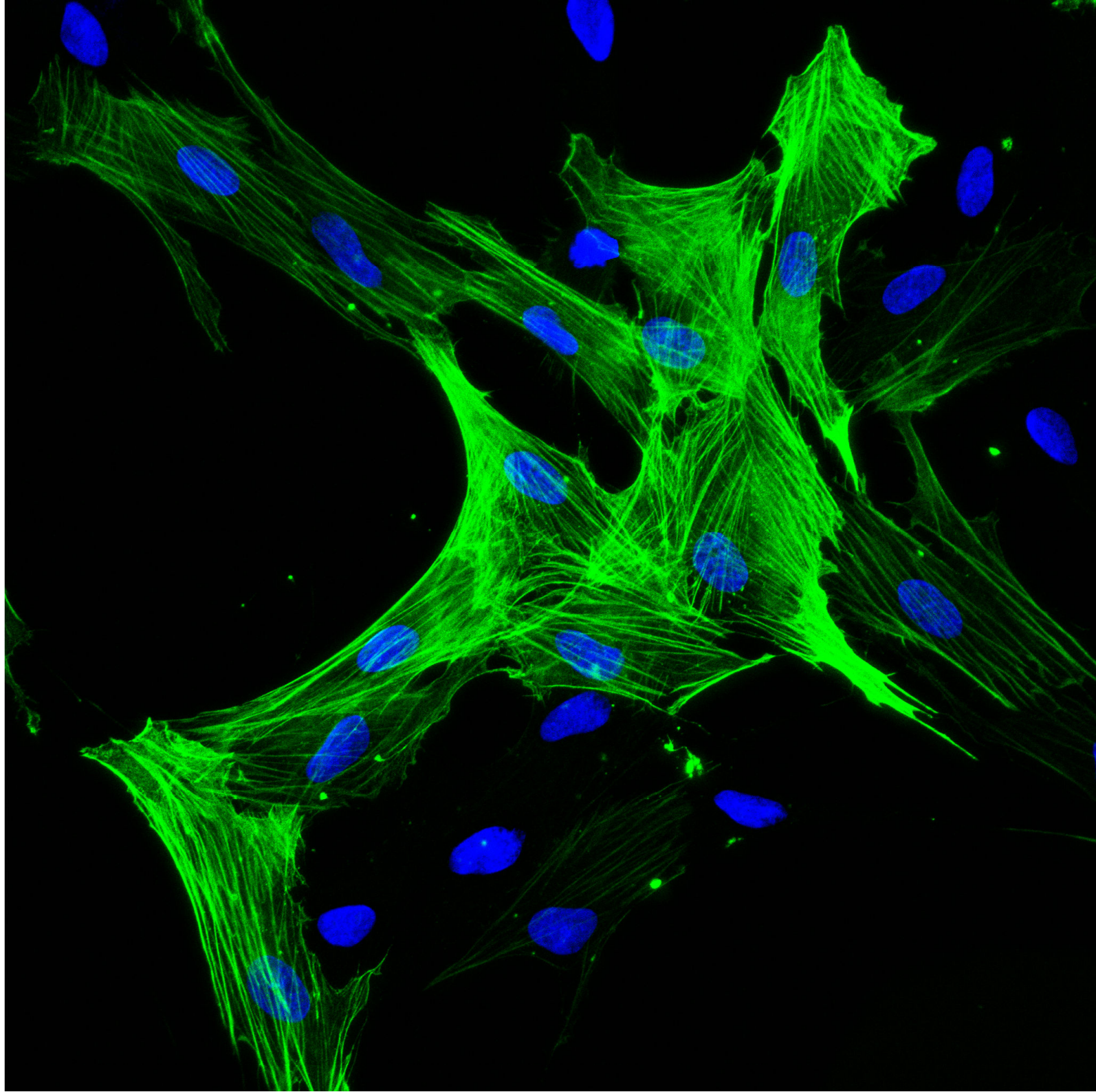

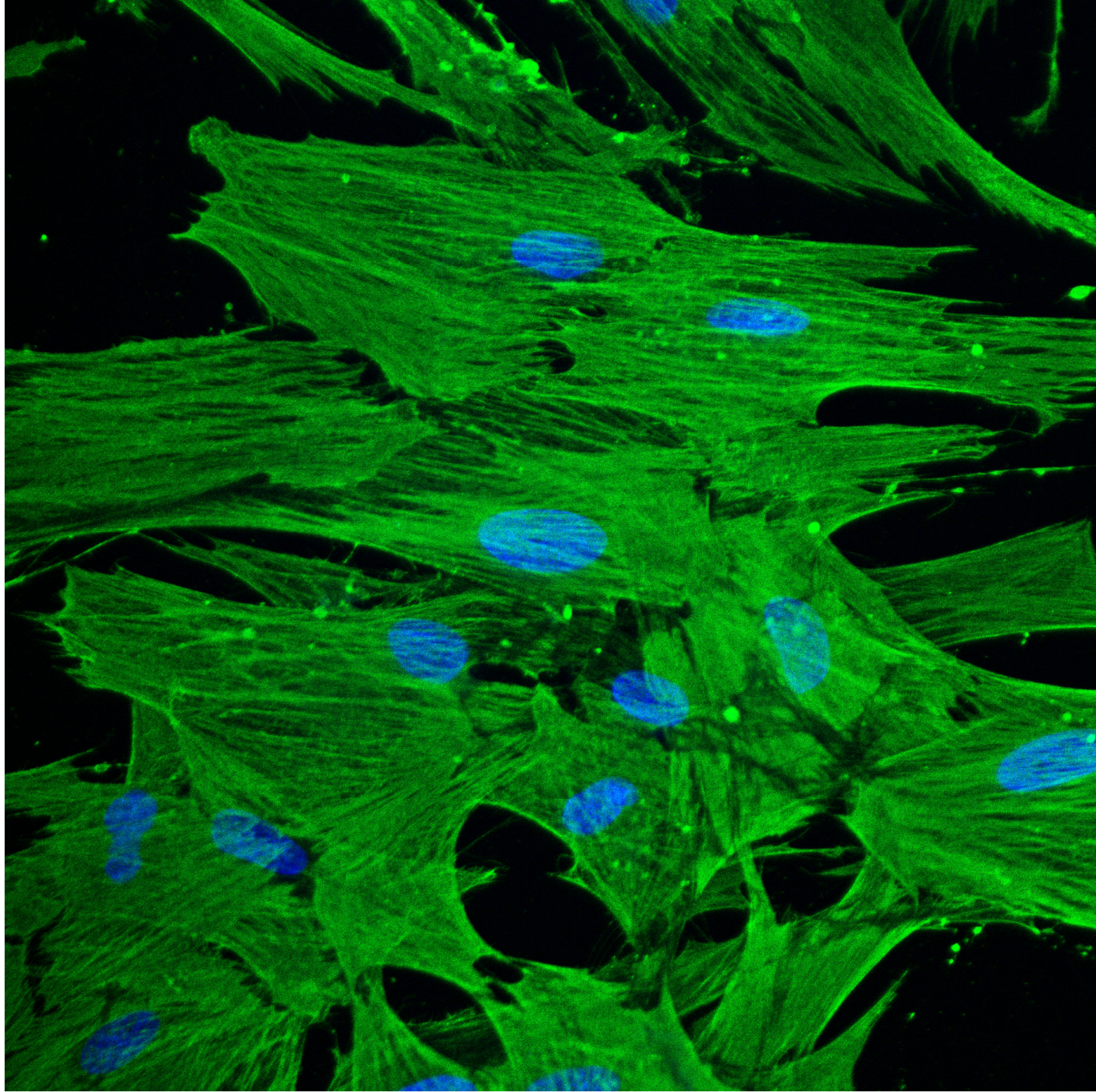

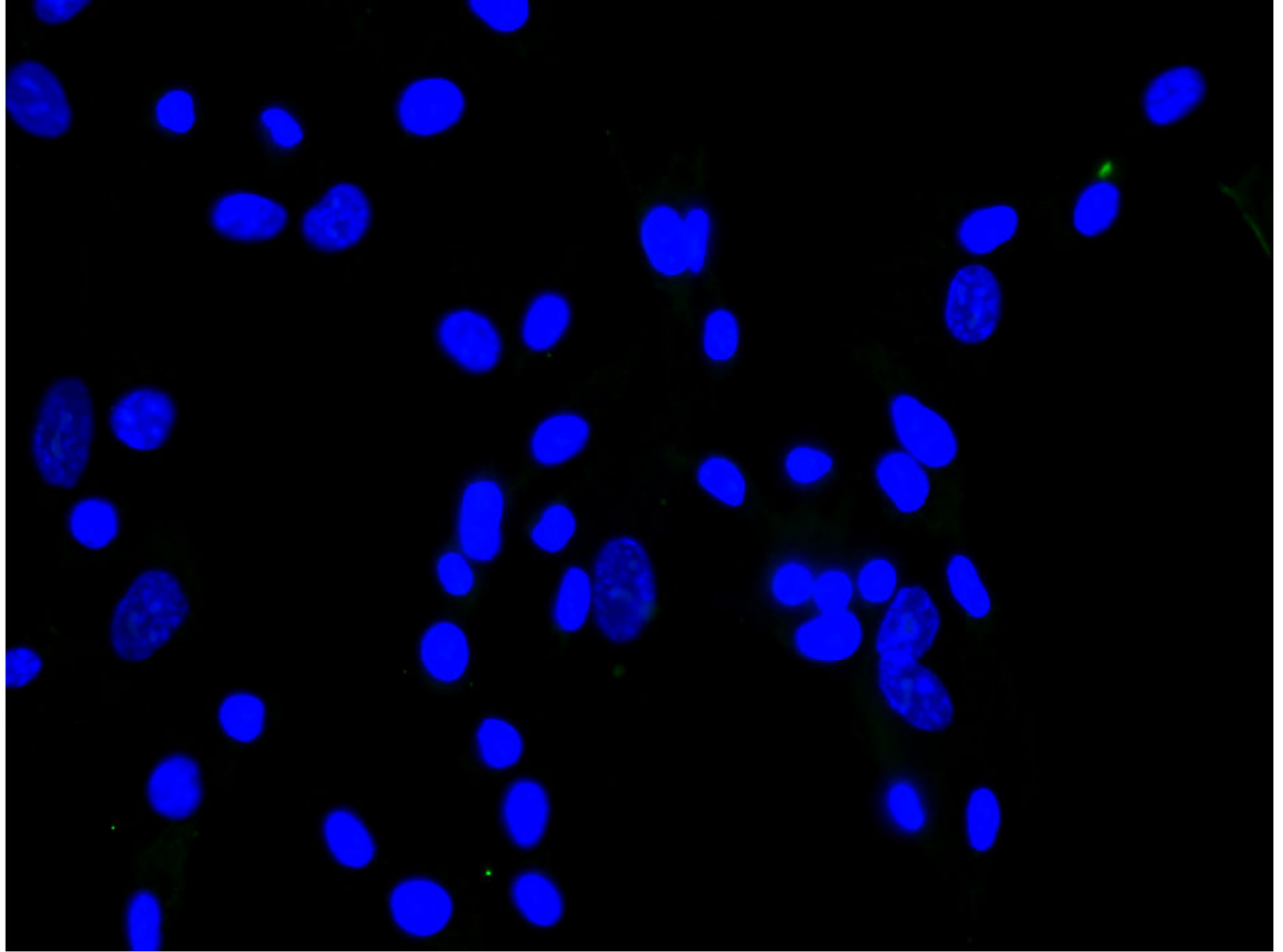

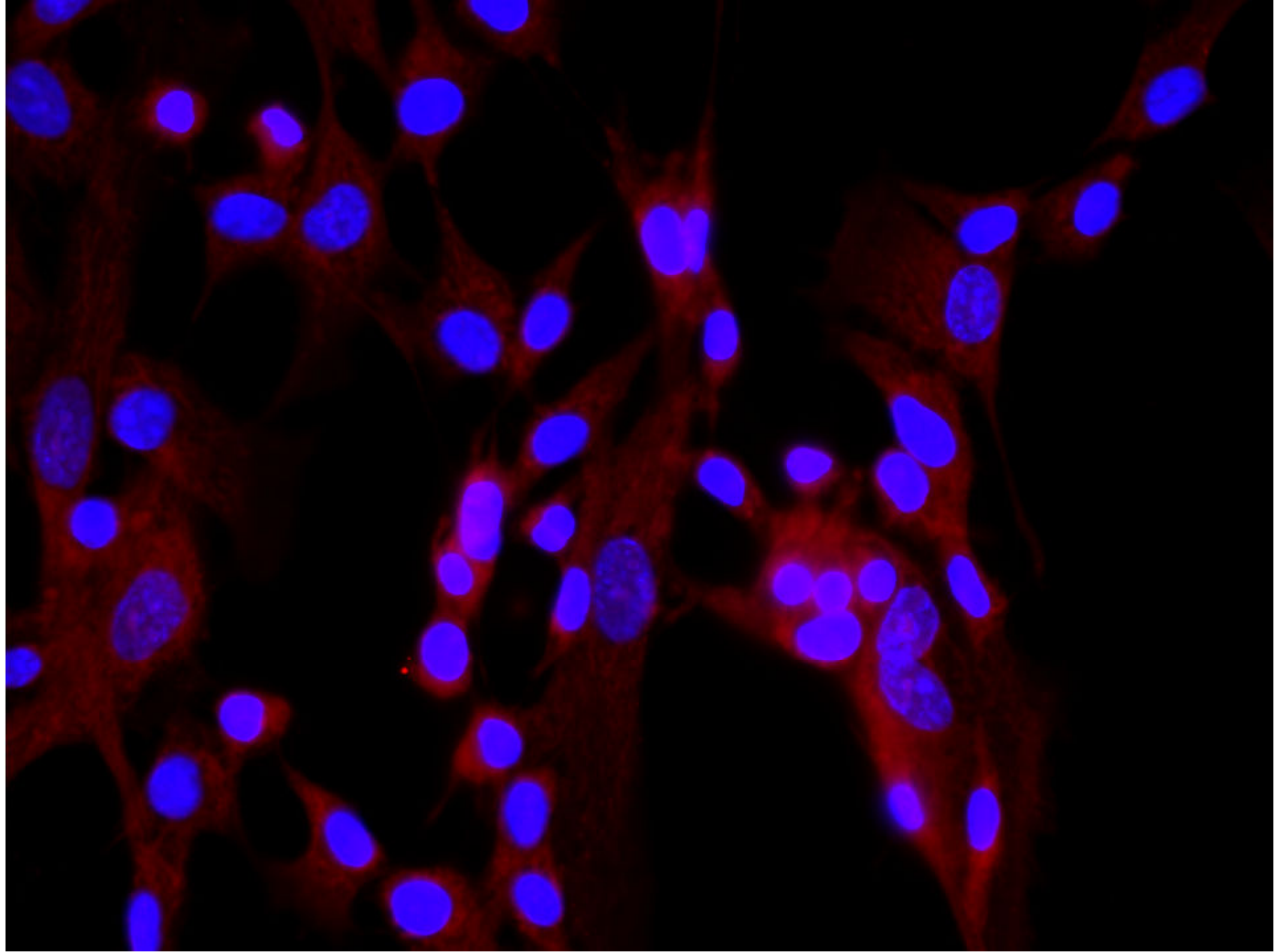

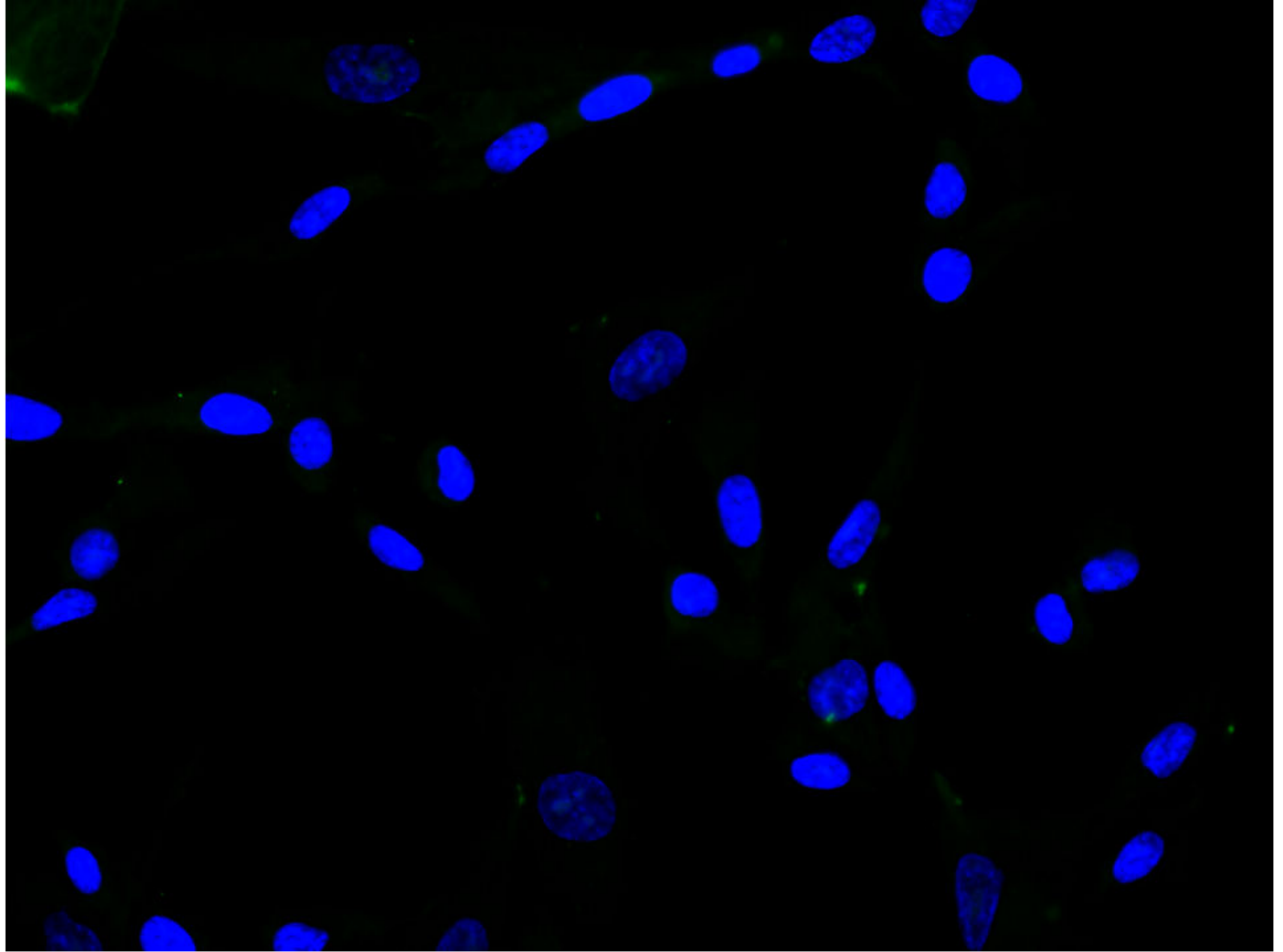

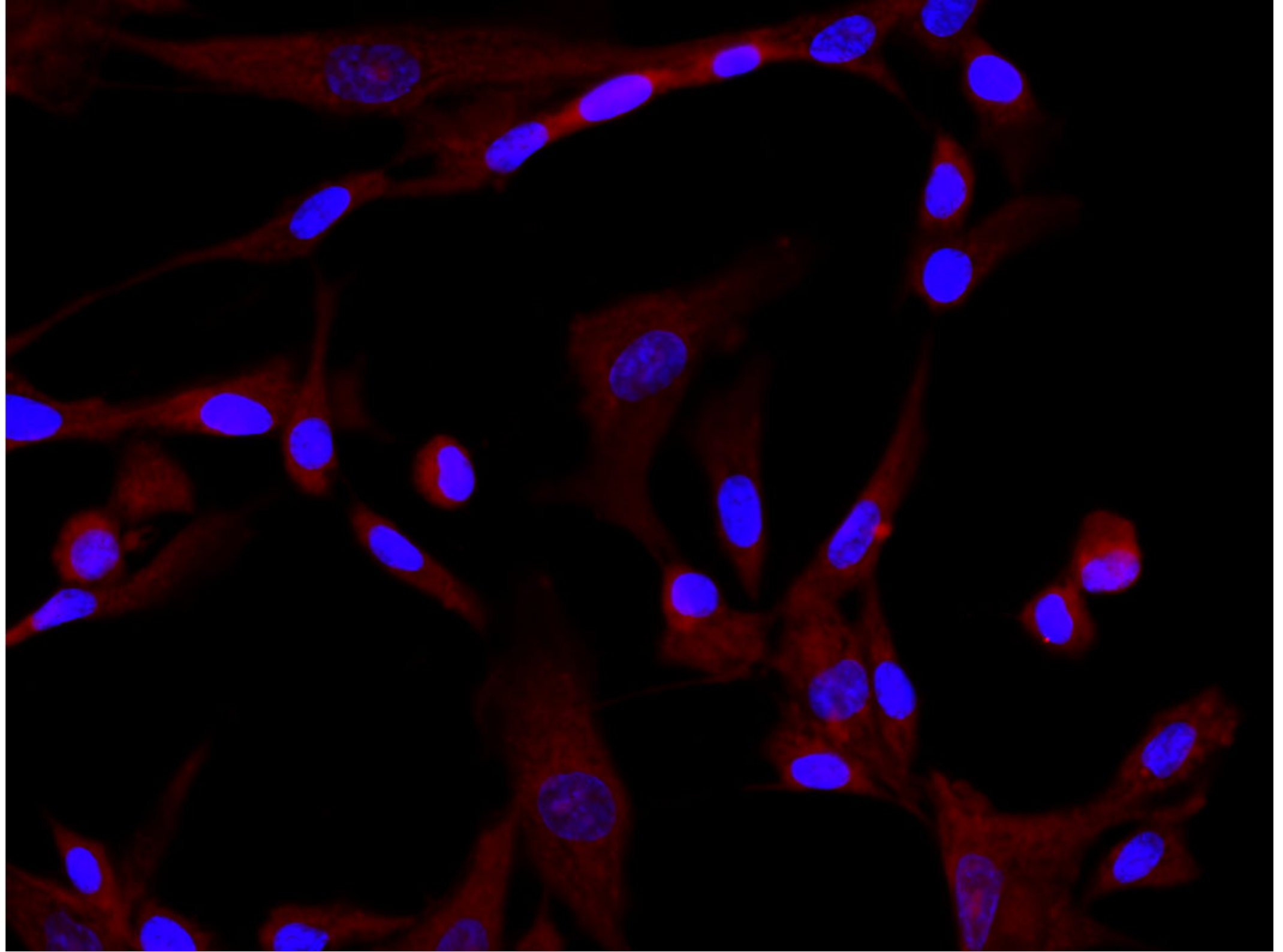

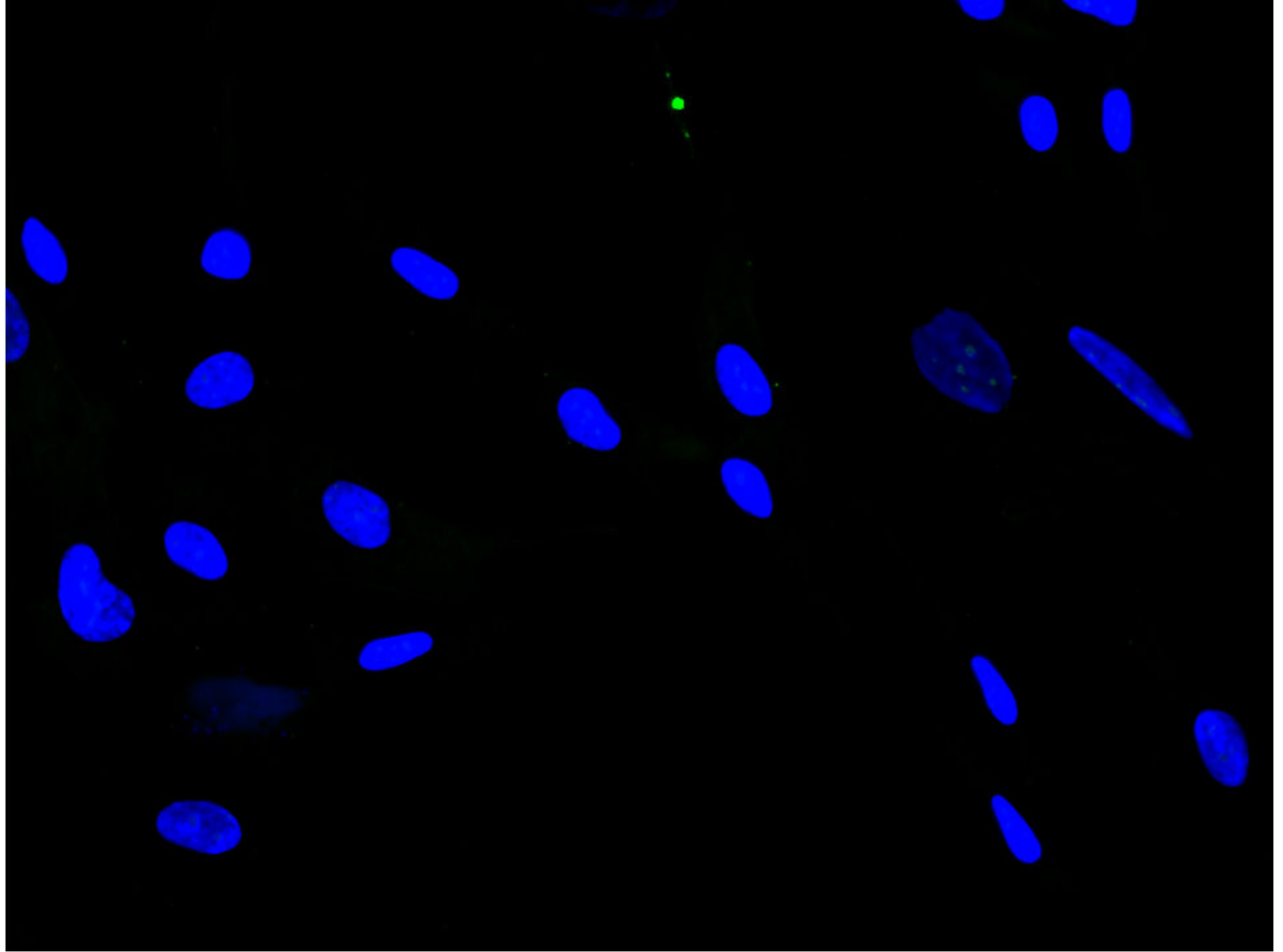

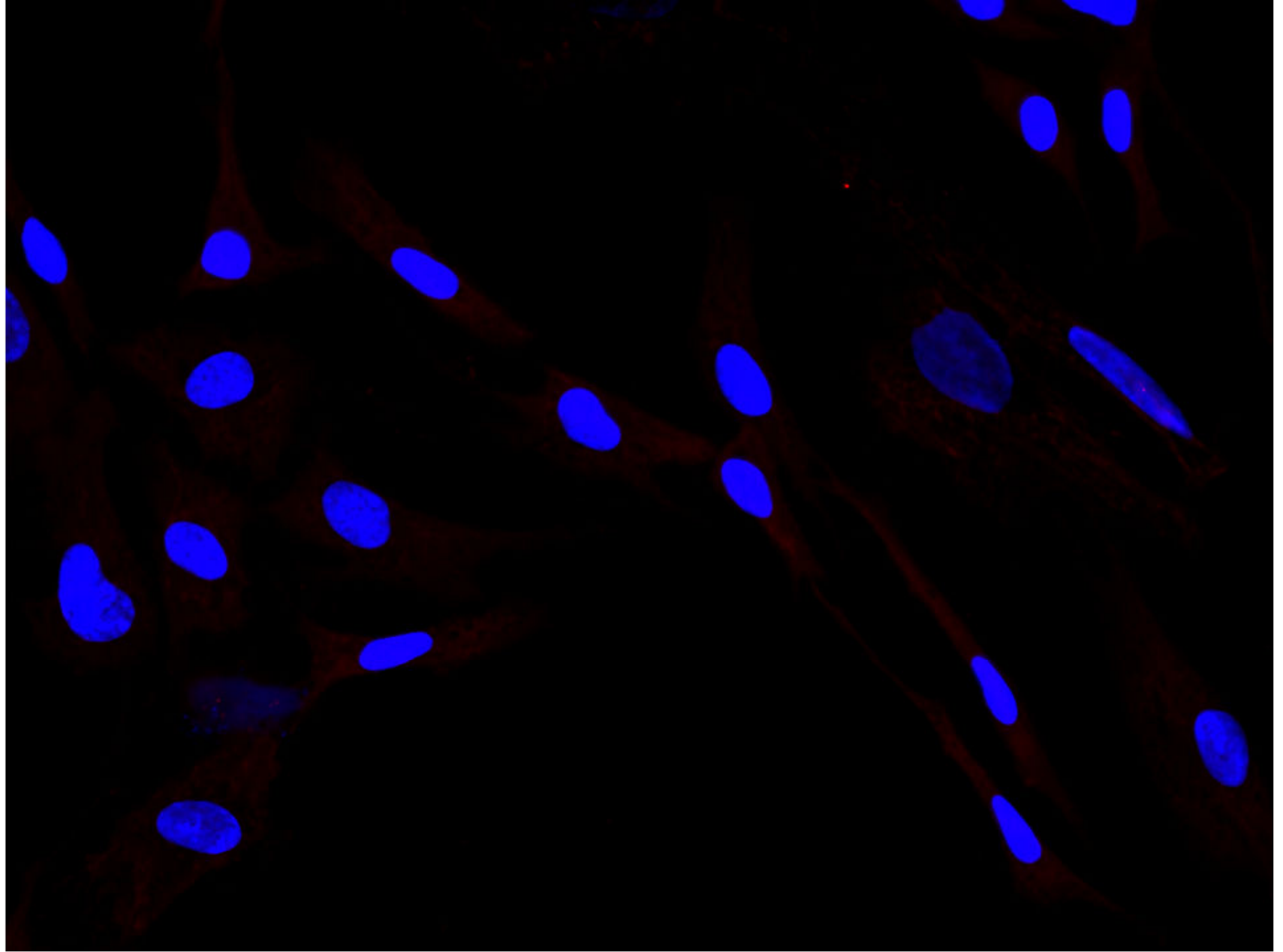

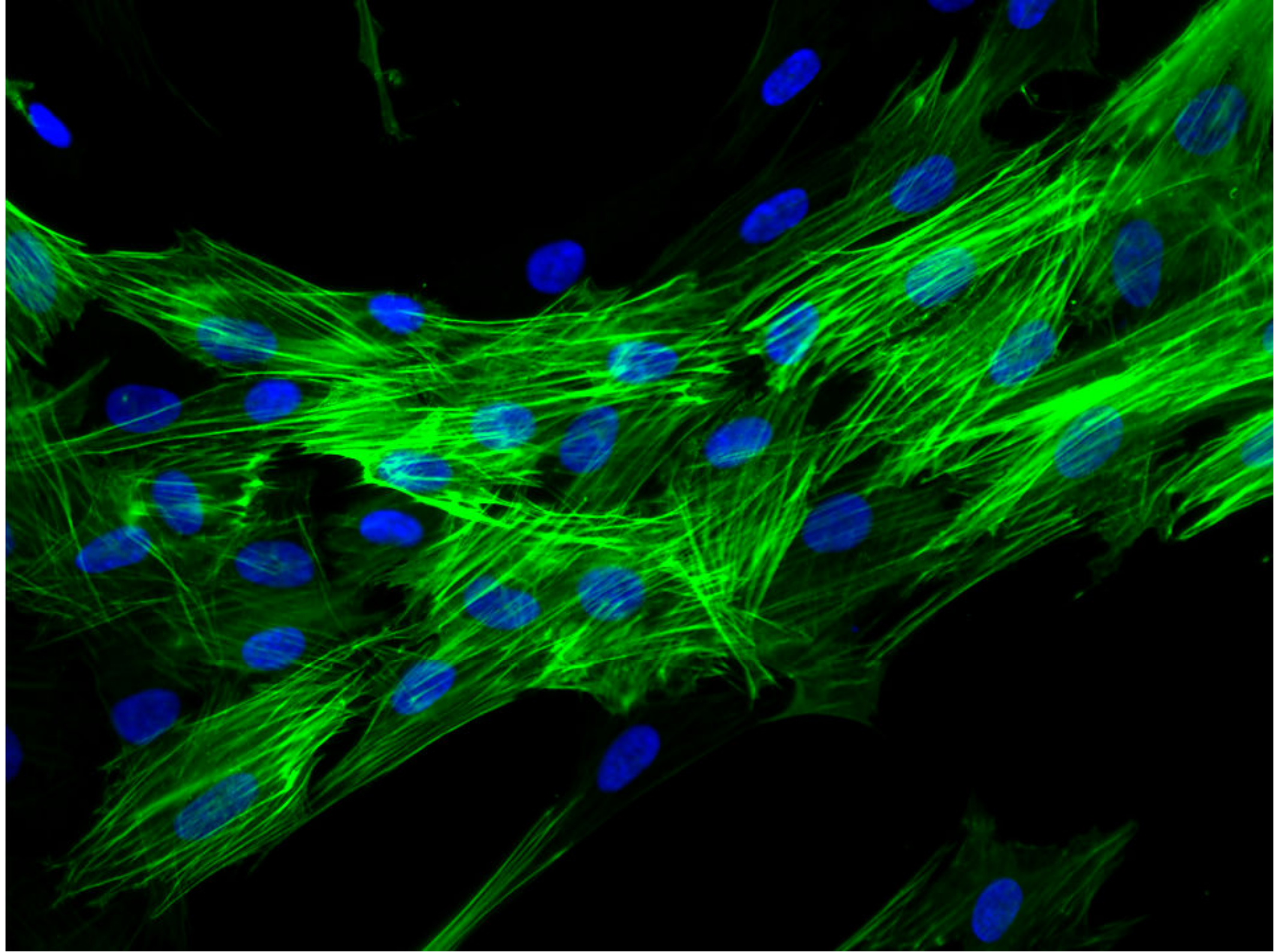

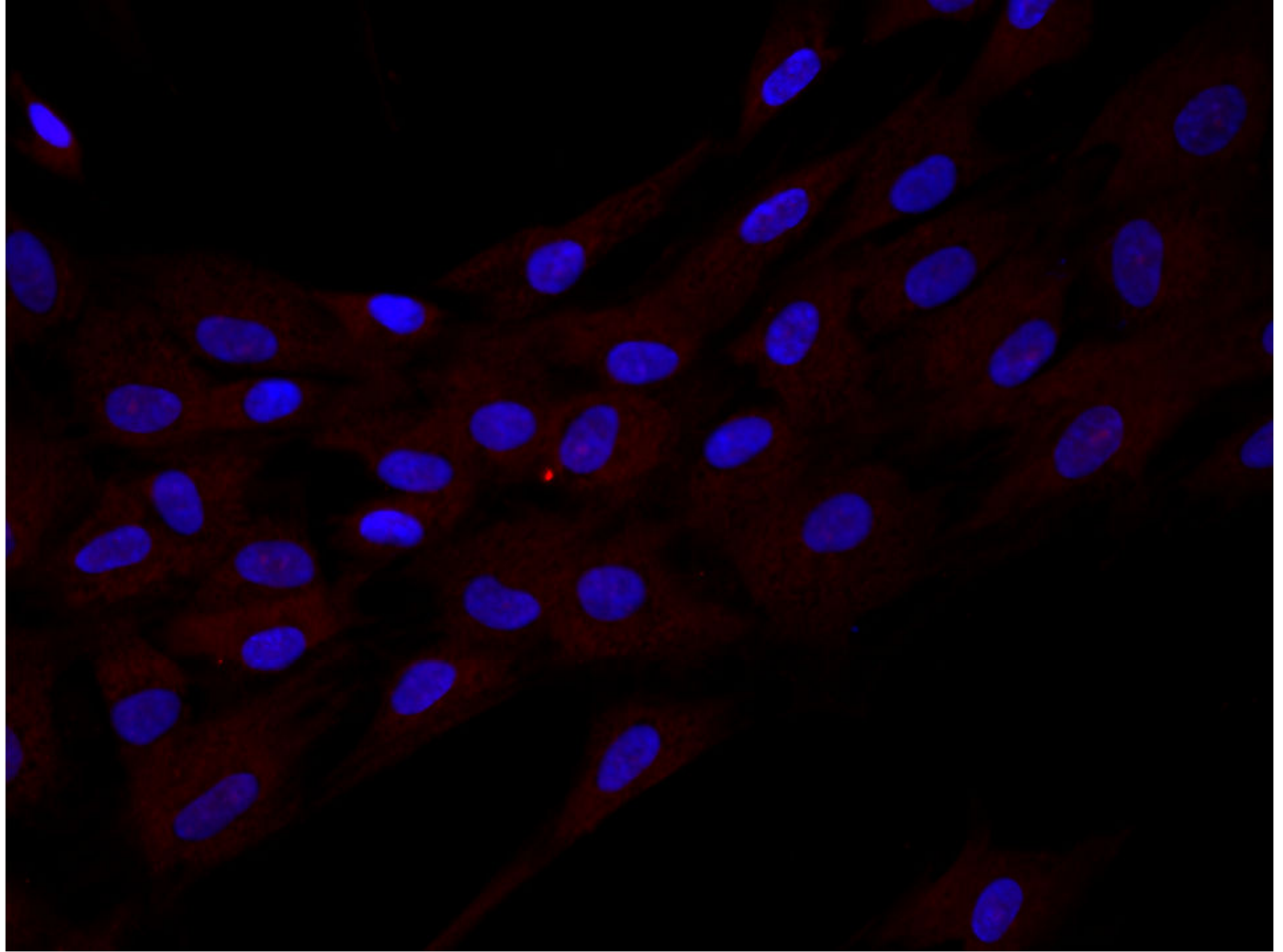

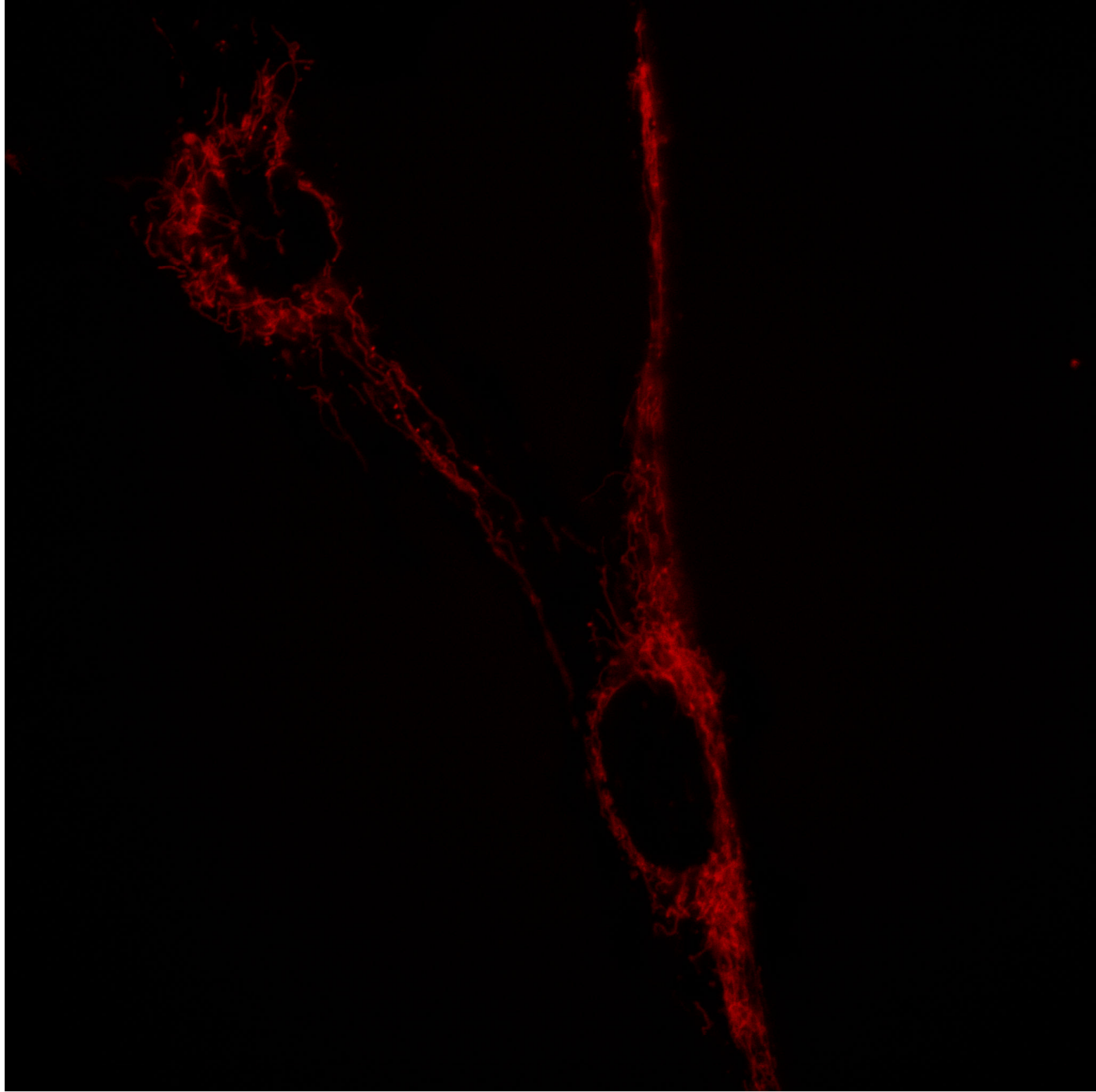

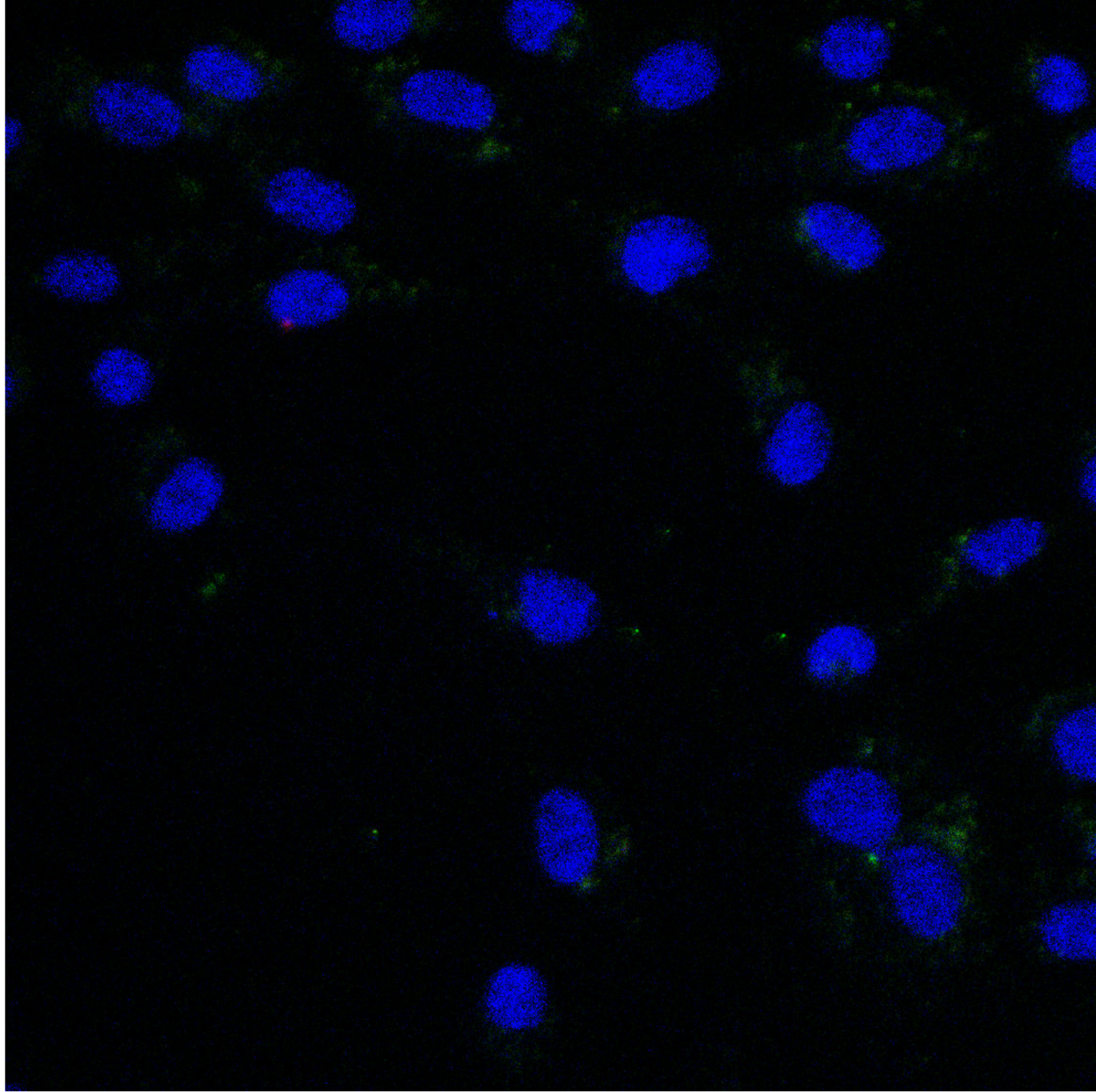

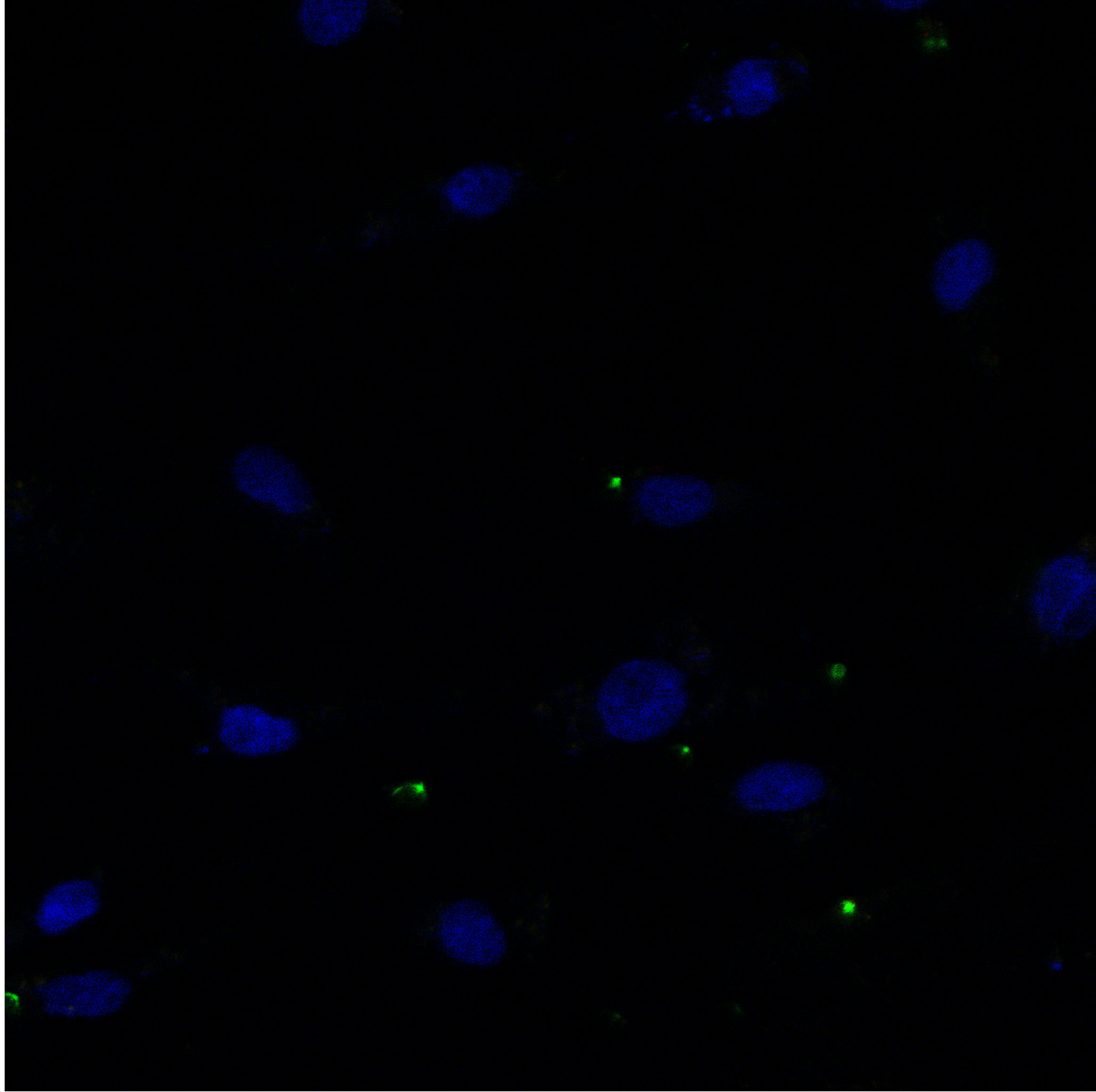

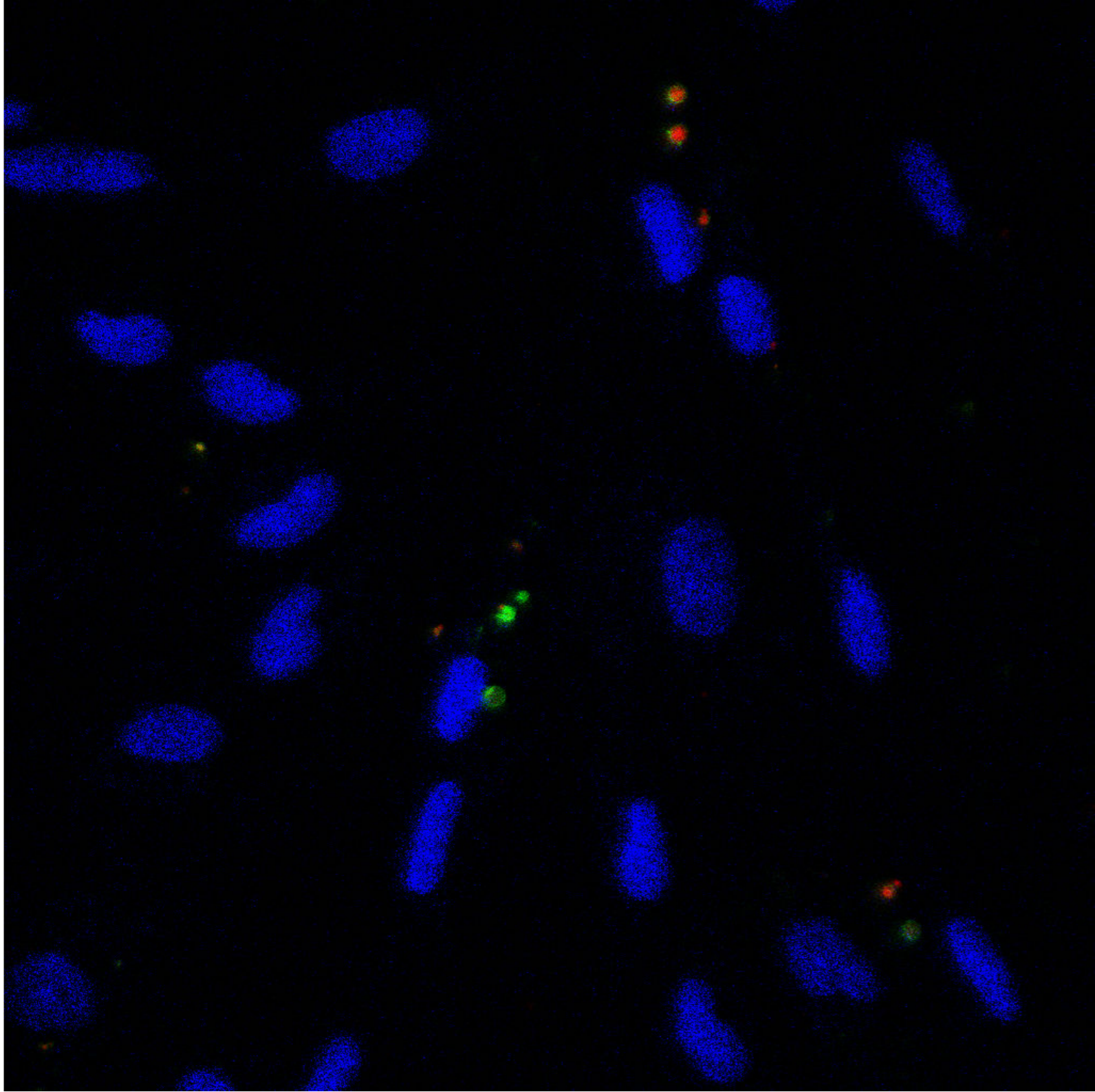

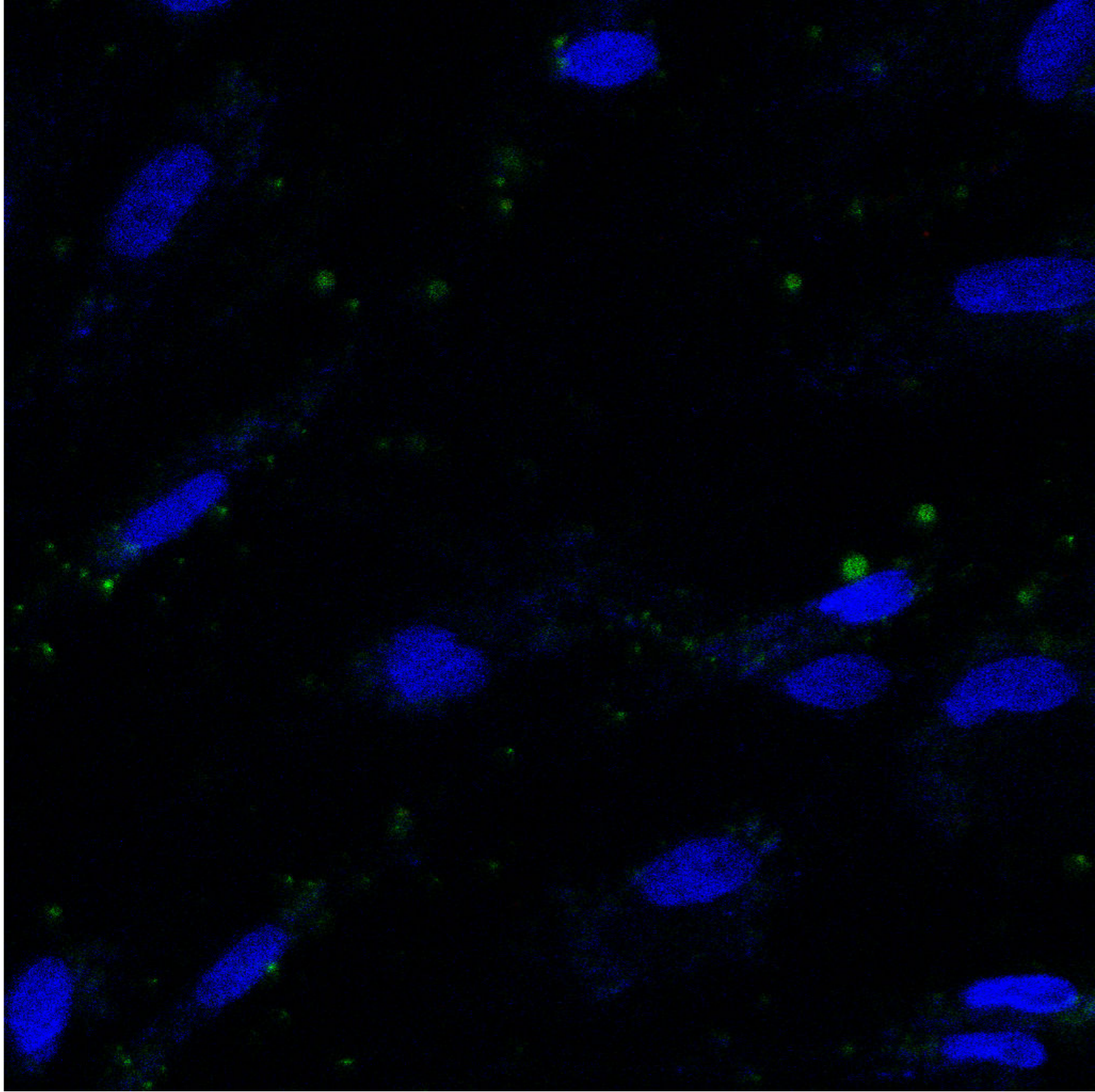

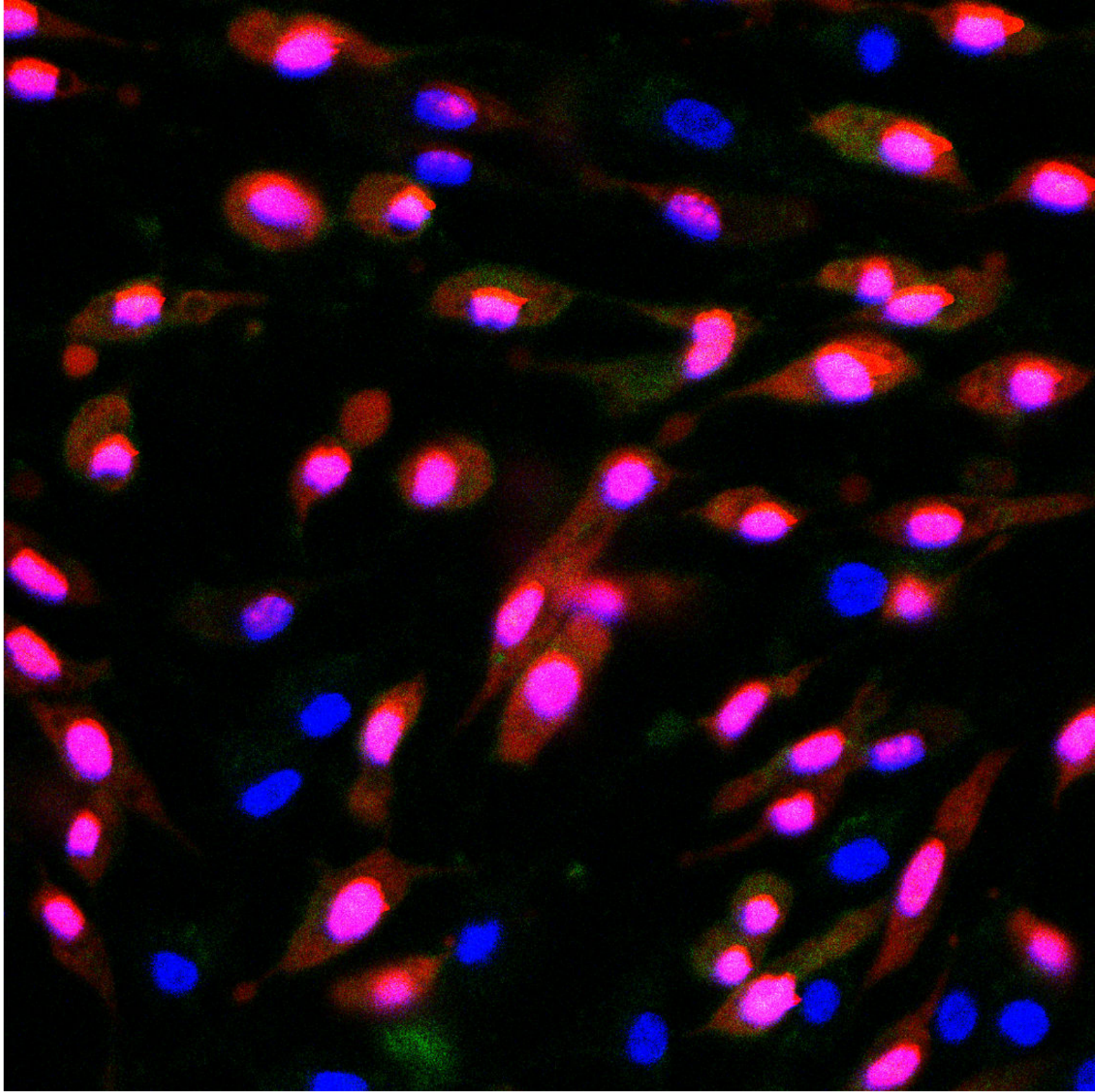

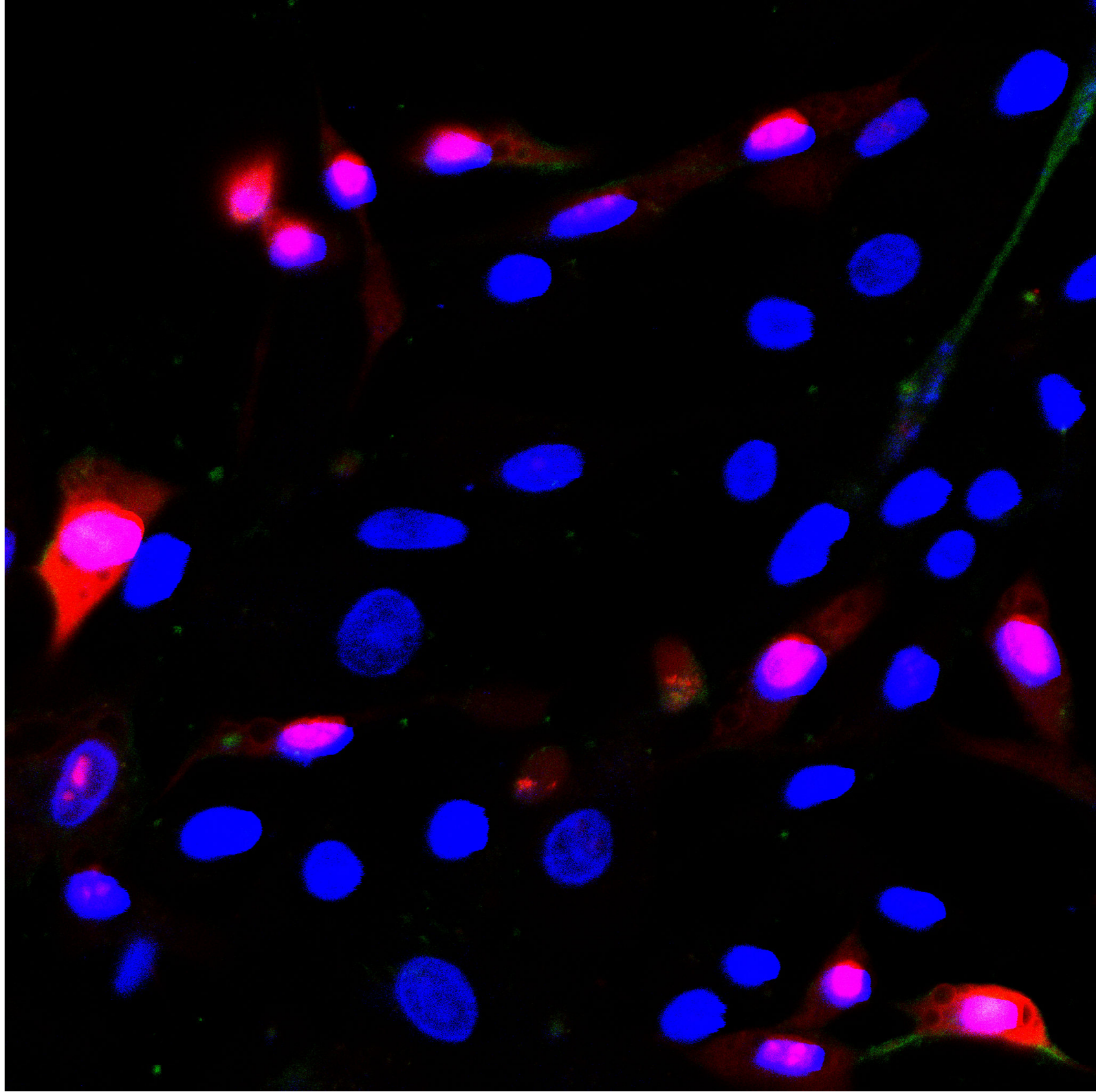

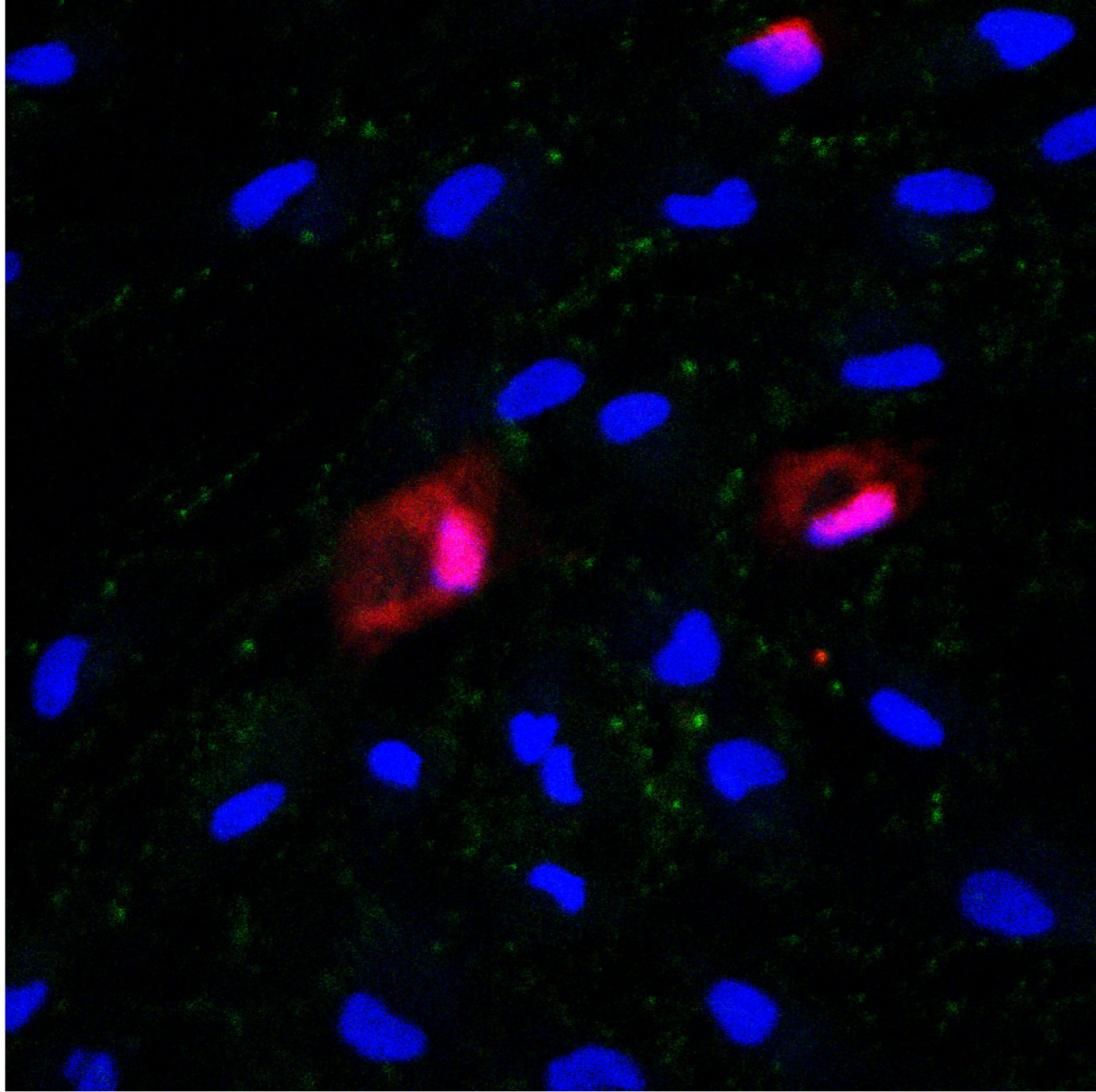

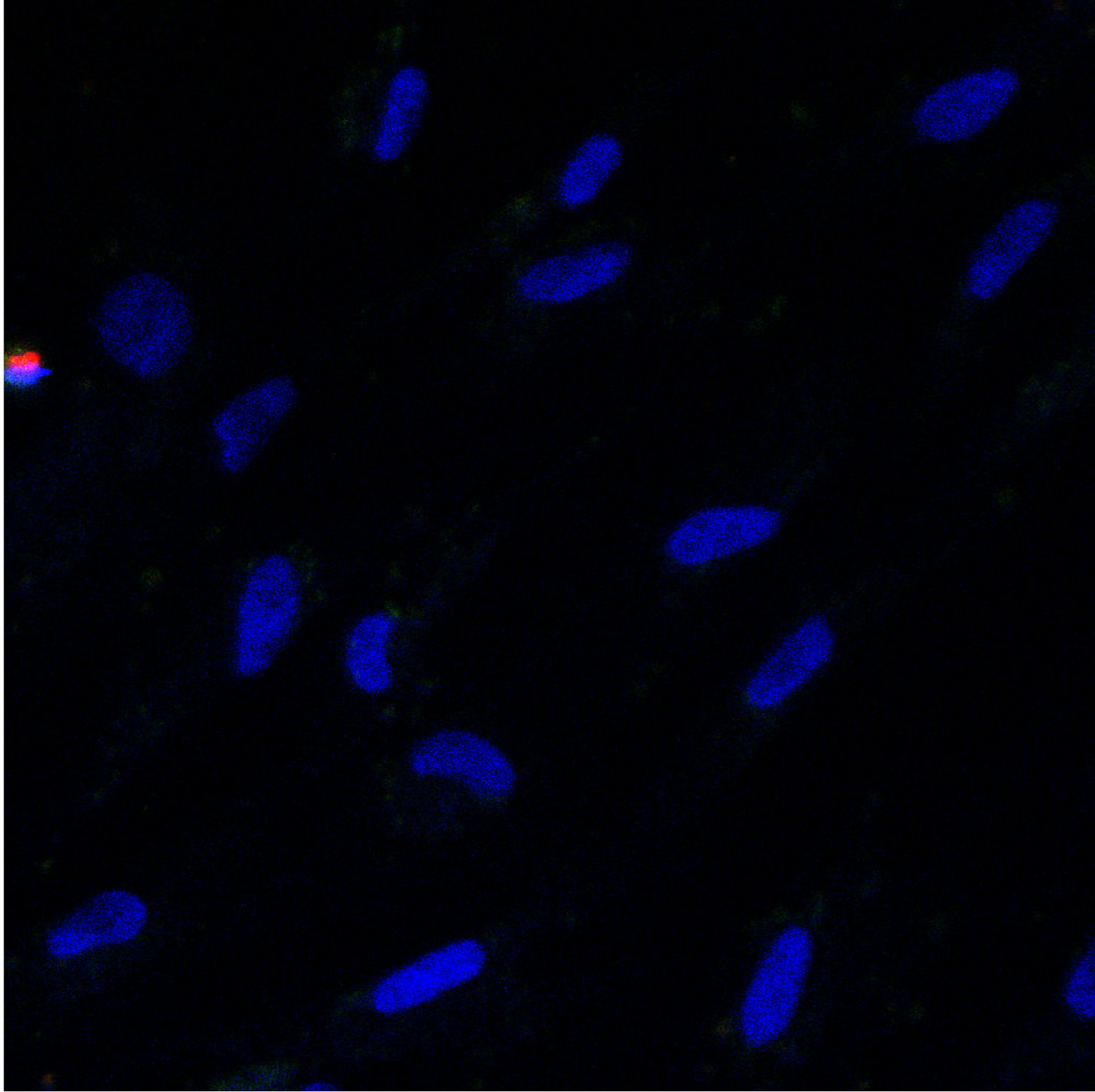

Supplement: Supplementary file 3 — Original Data File [file 41419_2022_5186_MOESM3_ESM.pdf]
